# *In vivo* RNAi screen and validation reveals Ngp, Hba-a1, and S100a8 as novel inhibitory targets on T lymphocytes in liver cancer

**DOI:** 10.3389/fimmu.2025.1549229

**Published:** 2025-04-25

**Authors:** Inga Hochnadel, Lisa Hoenicke, Nataliia Petriv, Huizhen Suo, Lothar Groebe, Chantal Olijnik, Nina Bondarenko, Juan C. Alfonso, Michael Jarek, Ruibing Shi, Andreas Jeron, Kai Timrott, Tatjana Hirsch, Nils Jedicke, Dunja Bruder, Frank Klawonn, Ralf Lichtinghagen, Robert Geffers, Henrike Lenzen, Michael P. Manns, Tetyana Yevsa

**Affiliations:** ^1^ Department of Gastroenterology, Hepatology, Infectious Diseases and Endocrinology, Hannover Medical School (MHH), Hannover, Germany; ^2^ Experimental Immunology, Helmholtz Centre for Infection Research (HZI), Braunschweig, Germany; ^3^ Department of Pathological Anatomy, Forensic Medicine and Pathological Physiology, Dnipro State Medical University, Dnipro, Ukraine; ^4^ Department of Systems Immunology, Technical University Braunschweig and HZI, Braunschweig, Germany; ^5^ Genome Analytics, HZI, Braunschweig, Germany; ^6^ Biostatistics Research Group, HZI, Braunschweig, Germany; ^7^ Immune Regulation Group, HZI, Braunschweig, Germany; ^8^ Infection Immunology Group, Institute of Medical Microbiology and Hospital Hygiene, Otto-von-Guericke University Magdeburg, Magdeburg, Germany; ^9^ Department of General, Visceral and Transplant Surgery, MHH, Hannover, Germany; ^10^ Munich Biomarker Research Center, Institute of Laboratory Medicine, German Heart Center, Technical University of Munich, Munich, Germany; ^11^ Department of Computer Science, Ostfalia University, Wolfenbüttel, Germany; ^12^ Department of Clinical Chemistry, MHH, Hannover, Germany; ^13^ Department of Gastroenterology, Hepatology, Interventional Endoscopy and Diabetology, Academic Teaching Hospital Braunschweig, Braunschweig, Germany

**Keywords:** RNA interference screen, T lymphocytes, hepatocellular carcinoma, immunotherapy, immune checkpoint inhibitors

## Abstract

**Background:**

Hepatocellular carcinoma (HCC) represents the third deadliest cancer worldwide with limited treatment options. Immune checkpoint inhibitors (ICIs) have revolutionized HCC therapy, but immune suppression within the tumor microenvironment remains a major challenge. Therefore, in this study, we aimed to define novel ICI molecules arising on T cells during aggressive HCC development.

**Methods:**

Using autochthonous HCC models, we performed microarray analyses followed by *in vivo* RNA interference screen and identified several new ICI molecules on CD4 and CD8 T lymphocytes in HCC-bearing mice. Short hairpin RNA (shRNA)-mediated knockdown of the ICI molecules was performed to validate their functional role in T cell activity and survival of HCC-bearing mice. Finally, we searched for the presence of the defined ICI molecules in HCC patients.

**Results:**

We identified neutrophilic granule protein (*Ngp*), hemoglobin subunit alpha-1 (*Hba-a1*), and S100 calcium-binding protein a8 (*S100a8*) as novel inhibitory molecules of T cells in HCC. The specific shRNA-based knockdown of these inhibitory targets was safe, led to a downregulation of classical ICI molecules (PD-1, PD-L1, 4-1BBL, CD160), and kept liver parameters under control in murine HCC. Besides, we detected upregulation of *S100A8* and *S100A9* in blood and liver tissues in HCC patients, supporting their clinical relevance.

**Conclusion:**

The obtained results pave the way for the use of the newly defined ICI molecules *Ngp*, *Hba-a1*, and *S100a8* as novel immunotherapeutic targets in further preclinical and clinical studies in HCC patients.

## 1 Introduction

Hepatocellular carcinoma (HCC) is a highly lethal cancer that represents the third most common cause of cancer-related deaths worldwide, with about 830,000 patients dying from the disease annually (1, 2). The primary risk factors for HCC include cirrhosis and chronic infection with hepatitis B or C viruses (3). HCC incidence continues to rise globally, and most HCC cases are estimated to occur in Asia (72%) followed by Europe (10%), Africa (7.8%), and least cases in Oceanic (0.5%) (4). Current treatment options for HCC, like surgical liver resection, liver transplantation, and locoregional therapies, including radiofrequency ablation (5) and transarterial chemoembolization, are limited to very early stages of the malignant disease and cannot prevent recurrence (6). In addition, in the majority of patients (>80%), HCC is diagnosed in unresectable tumor stages, thereby limiting the treatment options to systemic therapies (7). Sorafenib, a multikinase kinase inhibitor, used to be a standard therapy for HCC since 2007 (8). Immunotherapy recently replaced sorafenib, as the first-line therapy in unresectable HCC (9), as described below.

Considering the permanently growing incidence of HCC and the limited efficacy of current therapies, there is an urgent need for new innovative treatment strategies. Since HCC is modulating the tumor microenvironment (TME) (10) to evade the immune system, immunotherapy represents an attractive alternative to target and re-activate immune cells, which became dysfunctional due to suppression by HCC. Importantly, since HCC has been shown to be immunogenic and several HCC-specific tumor-associated antigens that are targeted by T cells have been identified (11–13), T cell-based immunotherapy is considered as a promising treatment. It has been shown that T lymphocytes are highly infiltrating HCC, which is also correlating with better survival prognosis (14–16). Nevertheless, tumor-infiltrating T lymphocytes or tumor-specific T lymphocytes in close proximity to tumor, are found to be exhausted and display an over-expression of several immune checkpoint inhibitors (ICIs) in which programmed cell death protein 1 (PD-1), cytotoxic T-lymphocyte-associated protein-4 (CTLA-4), T-cell immunoglobulin and mucin-domain containing-3 (TIM-3), V-domain Ig suppressor of T cell activation (VISTA) and lymphocyte activating 3 (LAG3) are frequently studied (17, 18). In line with above mentioned, recently approved therapies for unresectable HCC comprising atezolizumab and bevacizumab, inhibitors of PD-L1 and vascular endothelial growth factor (VEGF), showed better prognosis in HCC patients and were approved as the first-line therapy for unresectable HCC (9). In addition, many ongoing clinical trials are evaluating antibodies targeting specific ICIs as a single-agent therapy. However, combination strategies have been shown to be more effective in treating this complex malignant disease (18–20).

The discovery of ICIs has revolutionized cancer treatment, but it still needs to be further investigated, and new inhibitory targets need to be explored. Therefore, we performed microarray analysis on CD4^+^ and CD8^+^ T lymphocytes isolated from HCC-bearing mice to identify further immune inhibitory molecules associated with HCC development. Additionally, we approved our findings by performing an RNA interference (RNAi) screen *in vivo*. Among several upregulated genes, we found neutrophilic granule protein (*Ngp)*, hemoglobin subunit beta-1 *(Hbb-b1)*, hemoglobin subunit alpha-1 *(Hba-a1)*, S100 calcium-binding protein a8 (*S100a8*), and others highly expressed on T lymphocytes in HCC-bearing mice. We performed *in vivo* validation experiments and investigated the functional role of *Ngp*, *Hbb-b1, Hba-a1*, and *S100a8* on T lymphocytes during HCC development. Using a short hairpin RNA (shRNA)-based specific knockdown of these targets in donor-derived T lymphocytes, we tested the impact of adoptive T cell transfer therapy on survival and ICIs repertoire in HCC-bearing animals and thereby identified the most promising targets. Furthermore, we confirmed our data obtained in a preclinical mouse model in samples obtained from HCC patients.

## 2 Materials and methods

The section “Materials and Methods” can be found in **Supplementary Materials.**

## 3 Results

### 3.1 HCC development and isolation of T lymphocytes for microarray analysis

In the first part of our study, we aimed to identify to date unknown inhibitory molecules on CD4 and CD8 T lymphocytes, which are upregulated during HCC development using microarray analysis (see “Experimental setup” in **Supplementary Figure S1**).

#### 3.1.1 HCC Model

To induce HCC development, we delivered transposons expressing two oncogenes, *NRAS^G12V^* and *c-Myc*, together with a Sleeping Beauty transposase (*SB13*) into C57BL/6-Foxp3^tm1Flv/^J mice expressing red fluorescent protein (RFP) regulatory T cells (Tregs) using the hydrodynamic tail vein injection (HDI) technique (**Supplementary Figure S1A**). Control tumor-free mice received either *NRAS^G12V^* or *c-Myc* with *SB13* (**Supplementary Figure S1A**). After 5-8 weeks post-HDI, mice with overexpression of both oncogenes (*NRAS^G12V^* and *c-Myc*) developed tumors and were sampled together with the corresponding tumor-free controls (*NRAS^G12V^* or *c-Myc*) (**Supplementary Figure S2A**).

#### 3.1.2 Isolation of CD4 and CD8 memory T cells

Several organs (liver, liver-draining lymph nodes (later designated as: relevant lymph nodes (relLN)), not liver-draining lymph nodes (later designated as: irrelevant lymph nodes (irrelLN)), and spleen) were isolated and single-cell suspensions thereof were prepared (**Supplementary Figure S1B**). Cell suspensions were stained using the established protocols (21–24) and sorted for memory CD4 and CD8 T cells (**Supplementary Figure S1B**): CD3^+^ NK1.1^-^ CD4^+^ CD8^-^ Foxp3^-^ CD44^+^ (designated as CD4^+^ CD44^+^ T cells) and CD3^+^ NK1.1^-^ CD4^-^ CD8^+^ CD44^+^ T cell populations (designated as CD8^+^ CD44^+^ T cells), respectively. **Supplementary Figure S2B** demonstrates a gating strategy used at sorting to define both populations of memory CD4 and CD8 T cells.

#### 3.1.3 RNA isolation and microarray analysis

In the next step, total RNA from CD4^+^ CD44^+^ and CD8^+^ CD44^+^ T cells was isolated and processed for microarray analysis (**Supplementary Figure S1C**).

We performed in total two independent experiments giving rise to two independent replicates for each organ (liver, relLN, irrelLN, spleen) and cell type (CD4^+^ CD44^+^ and CD8^+^ CD44^+^ T cells).

### 3.2 Microarray analysis reveals 72 upregulated genes in CD4^+^ CD44^+^ and CD8^+^ CD44^+^ T lymphocytes during HCC development

To identify genes that are upregulated in T lymphocytes during HCC development, we conducted a microarray analysis comparing CD4^+^ CD44^+^ and CD8^+^ CD44^+^ T cells isolated from HCC-bearing (genotype HCC: *NRAS^G12V^*/*c-Myc*) mice with those isolated from HCC-free control animals (genotype C1: *c-Myc*; genotype C2: *NRAS^G12V^*, **Supplementary Figure S1A**, **Supplementary Figure S2A**). To identify target genes for validation studies, the log2 values in T lymphocytes originating from HCC-bearing mice were analyzed. Importantly, log2 values obtained in HCC-free controls were subtracted from log2 values of the tumor-bearing group. This allowed us to define genes that were consistently upregulated (enriched) in the TME of HCC-bearing animals. The term “enriched genes” in this context refers to those genes that were significantly upregulated in T cells from HCC-bearing mice compared to HCC-free controls.

**Figure 1:**
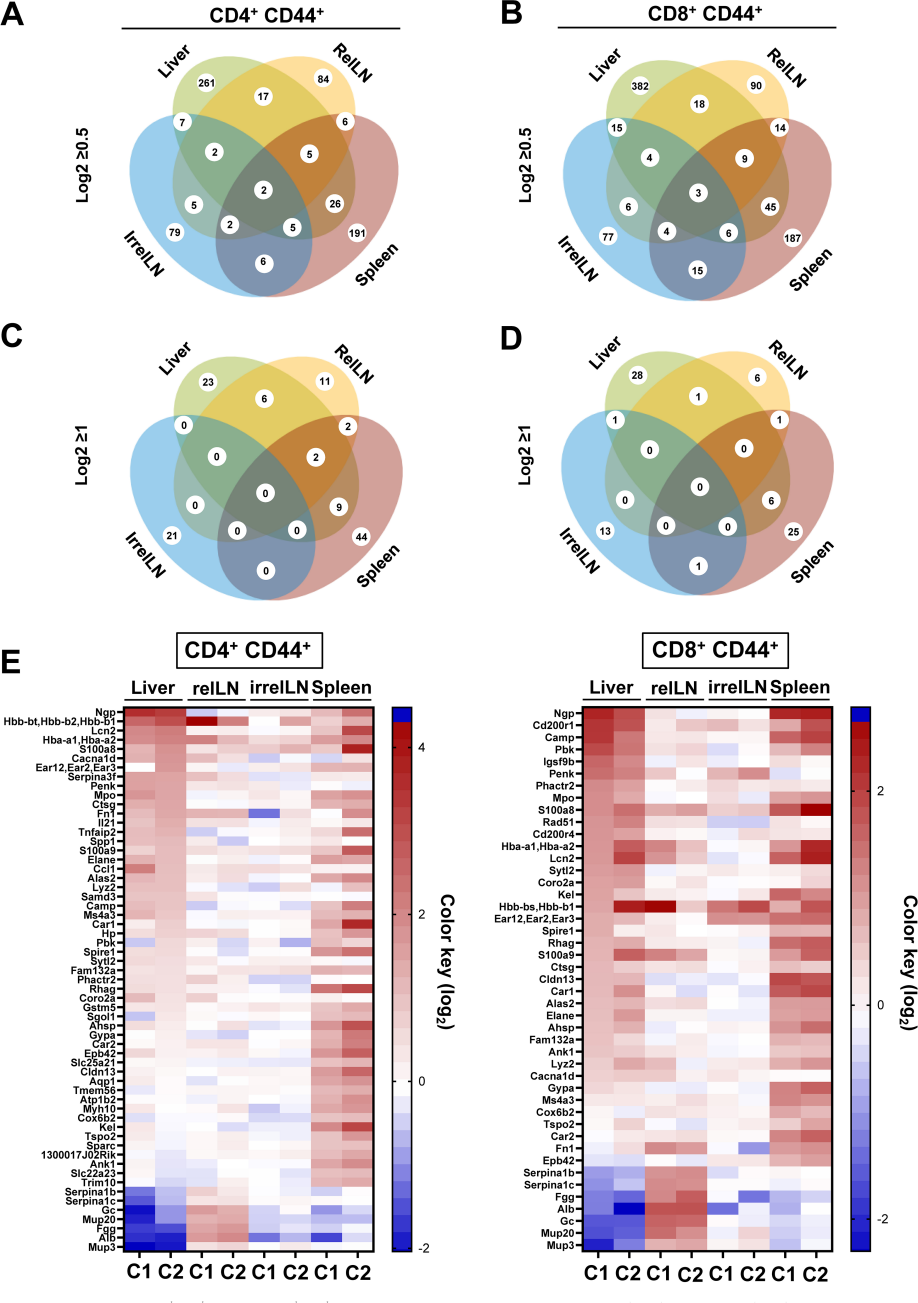
Microarray analysis on CD4^+^ CD44^+^ and CD8^+^ CD44^+^ T cells isolated from the liver, relLN, spleen, and irrelLN of HCC-bearing and HCC-free control mice. **(A-D)** Venn diagrams depicting the distribution of upregulated genes in **(A)** CD4^+^ CD44^+^ T cells with log2>0.5, **(B)** CD8^+^ CD44^+^ T cells with log2>0.5, **(C)** CD4^+^ CD44^+^ T cells with log2≥1, **(D)** CD8^+^ CD44^+^ T cells with log2>0.5. **(E)** Heatmap showing highly upregulated genes in CD4^+^ CD44^+^ and CD8^+^ CD44^+^ T cells isolated from liver, relLN, irrelN, and spleen of HCC-bearing mice, compared to HCC-free controls (C1: *c-Myc*/C2: *NRAS^G12V^*). Genes selected based on the microarray analysis were included as targets in the shRNA library for the *in vivo* RNAi screen. Data represents a pool of two independent experiments, with n=6 for each replicate (6 HCC-bearing mice vs. 6 HCC-free mice). HCC, hepatocellular carcinoma; relLN, relevant lymph nodes; irrelLN, irrelevant lymph nodes.

In CD4^+^ CD44^+^ T cells, we identified a total of 615 upregulated genes with log2≥0.5, distributed among the liver (261 genes), relLN (84 genes), spleen (191 genes), and irrelLN (79 genes) (**Figure 1A**). Among these, 17 genes were commonly upregulated in both liver and relLN, 26 genes in liver and spleen, 7 genes in HCC liver and irrelLN, and 5 genes in HCC liver, relLN, and spleen (**Figure 1A**). Intersections among spleen, relLN, and irrelLN ranged from 2 to 6 genes (**Figure 1A**).

Similarly, in CD8^+^ CD44^+^ T cells, we found 736 upregulated genes, with the liver showing the highest number (382 genes), followed by relLNs (90 genes), irrelLNs (77 genes), and spleen (187 genes) (**Figure 1B**). In comparison to CD4^+^ CD44^+^ T cells, CD8^+^ CD44^+^ T cells showed 121 additional genes that originated from the liver. Gene intersections among organs showed 18 genes shared between liver and relLN, 45 between liver and spleen, and 15 between liver and irrelLN (**Figure 1B**). Notably, 9 genes were identified in the intersection of liver, spleen, and relLN in CD8^+^ CD44^+^ T cells in comparison to CD4^+^ CD44^+^ T cells.

To narrow down our analysis, we next focused on genes with log2≥1, selecting 99 highly upregulated genes in CD4^+^ CD44^+^ T cells and 72 genes in CD8^+^ CD44^+^ T cells (**Figure 1C** for CD4^+^ CD44^+^ T cells and **Figure 1D** for CD8^+^ CD44^+^ T cells). These genes were distributed across different tissues: 23 genes in liver, 11 genes in relLN, 44 genes in spleen, 21 genes in irrelLN in CD4^+^ CD44^+^ T cells (**Figure 1C**). In CD8^+^ CD44^+^ T cells, 6 genes were shared from liver and spleen, 1 gene between liver and relLN, and 1 gene between liver and irrelLN (**Figure 1D**). No genes met the log2≥1 threshold across liver, relLN, and spleen in CD8 T cells (**Figure 1D**).

Finally, to visualize and prioritize the most biologically relevant targets, we used a heatmap highlighting 72 of the most upregulated (enriched) genes in HCC liver, relLN, and spleen (**Figure 1E**). These genes were selected for the *in vivo* RNAi screen to uncover new targets on CD4 and CD8 T cells in HCC.

### 3.3 *In vivo* RNAi screen and identification of key players in T cell inhibition during HCC development

To identify key players in T cell inhibition, we performed the RNAi screen targeting 72 of the most upregulated genes identified in the microarray analysis.

#### 3.3.1 RNAi screen setup

**Figure 2:**
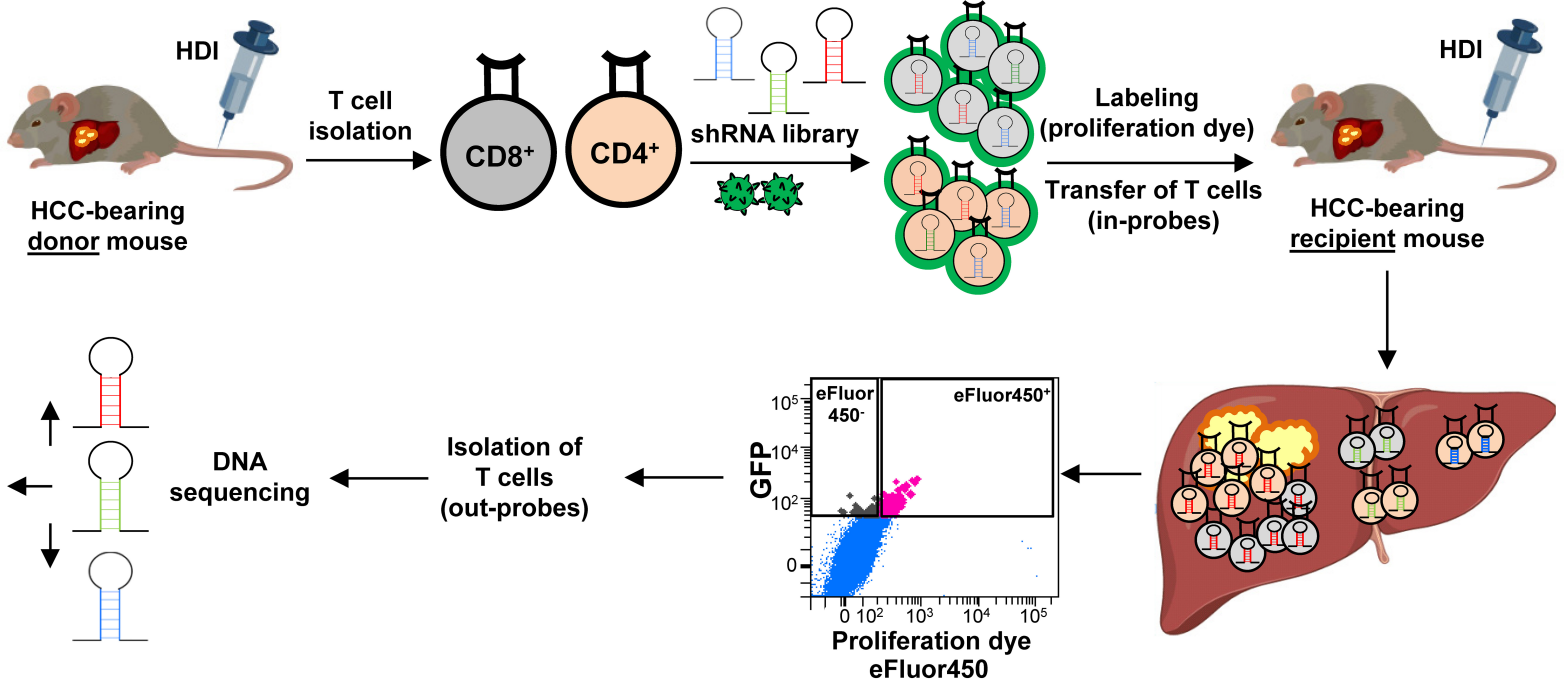
Experimental setup of the *in vivo* RNAi screen. CD4 and CD8 T cells were isolated from HCC-bearing mice (*NRAS^G12V^/c-Myc* genotype) and transduced with the shRNA library using a GFP-expressing lentivirus (pGIPZ). Successfully transduced GFP^+^ CD4 and CD8 T cells were sorted and labeled with a proliferation dye eFluor450 (in-probes). Thereafter, the cells were adoptively transferred into HCC-bearing recipient mice. Five days post-transfer, recipient mice were sacrificed, and the liver along with other organs were collected. Adoptively transferred T cells were isolated from the explanted organs, sorted for eFluor450^+^ and for eFluor450^-^ populations (out-probes), and subsequently subjected to DNA sequencing for shRNA representation/abundance analysis. HCC, hepatocellular carcinoma; HDI, hydrodynamic tail vein injection; GFP, green fluorescent protein; shRNA, short hairpin RNA.

We first induced HCC development using the HDI technique and isolated CD4 and CD8 T cells from HCC-bearing donor mice (**Figure 2**). These T cells were stimulated and transduced with a pooled shRNA library which contained 4-5 shRNAs per selected target gene. An shRNA targeting *Renilla* (shRen), a non-coding gene in mice, served as a negative control. The shRNA constructs were cloned into a third-generation pGIPZ lentiviral vector system expressing green fluorescent protein (GFP) allowing for a tracking of the transduction efficiency, as previously described (25). The lentiviral packaging was performed using a system consisting of pMD2.G, pMDLg/pRRE, and pRSV-Rev vectors, expressing envelope and transport proteins (VSV-G, Gag/Pol, Rev) (**Figure 2**). CD4 and CD8 T cells were transduced with the virus harboring the shRNA library. Following viral transduction, GFP^+^ CD4 and CD8 T cells were sorted (a gating strategy is shown in **Supplementary Figure S3**) and labeled with a proliferation dye (eFluor450). Thereafter, the transduced GFP^+^ eFluor450^+^ T cells were adoptively transferred into HCC-bearing recipient mice (**Figure 2**). For the adoptive transfer, GFP^+^ eFluor450^+^ CD4 and GFP^+^ eFluor450^+^ CD8 T cells were pooled and approximately 2x10^5^ cells were transferred intravenously (*i.v*.) (**Figure 2**). Five days post-transfer, the recipient mice were sacrificed, and liver, spleen, relLN, irrelLN, and blood were collected for further analysis. T cells were isolated and sorted according to CD3^+^ CD4^+^ GFP^+^ eFluor450^-/^CD3^+^ CD8^+^ GFP^+^ eFluor450^-^ and CD3^+^ CD4^+^ GFP^+^ eFluor450^+/^CD3^+^ CD8^+^ GFP^+^ eFluor450^+^ profiles (a gating strategy is shown in **Supplementary Figure S4**). Sorted CD4 and CD8 T cell populations were subjected to DNA isolation followed by Illumina sequencing to determine the shRNA representation/abundance (**Figure 2**). We searched for shRNA enrichment (log2-fold changes) detected in isolated T cells of HCC-bearing recipient mice (out-probes) compared to T cells prior to the adoptive transfer (original pool, in-probes). Enriched shRNAs (which we used to refer to overrepresented in out-probes, compared to the original pool (in-probes)) mean, that these shRNAs target inhibitory genes whose knockdown is beneficial for the proliferation of the corresponding T cell.

#### 3.3.2 RNAi screen data analysis

**Figure 3:**
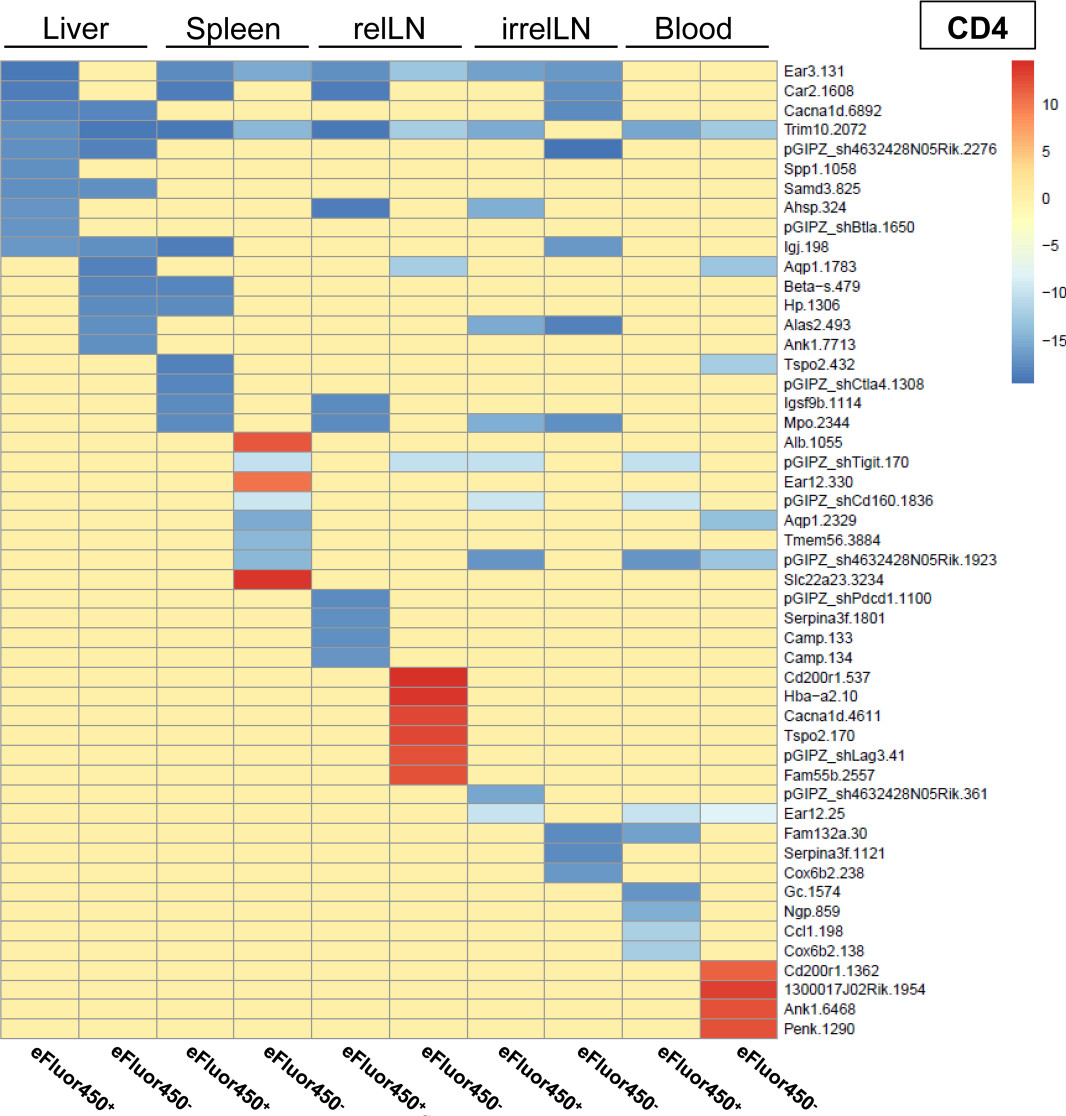
*In vivo* RNAi screen revealed several enriched shRNAs in CD4 T cells during HCC development. RNAi screen analysis was performed on eFluor450^-^ and eFluor450^+^ CD4 T cells isolated five days post-adoptive transfer from liver, spleen, relLN, irrelLN, and blood (out-probes) of HCC-bearing mice (*NRAS^G12V^/c-Myc* genotype). The CD4 T cells in out-probes were compared to the CD4 T cells before the adoptive transfer (in-probes). ShRNA enrichment (log2 fold-changes) detected in isolated CD4 T cells of HCC-bearing recipient mice (out-probes) compared to CD4 T cells before the adoptive transfer (in-probes) are presented in a heatmap. Upregulated shRNAs (>0) are marked in red and downregulated shRNAs (<0) are marked in blue. Data represent a pooled analysis from four recipient mice. relLN, relevant lymph nodes; irrelLN, irrelevant lymph nodes.

**Figure 4:**
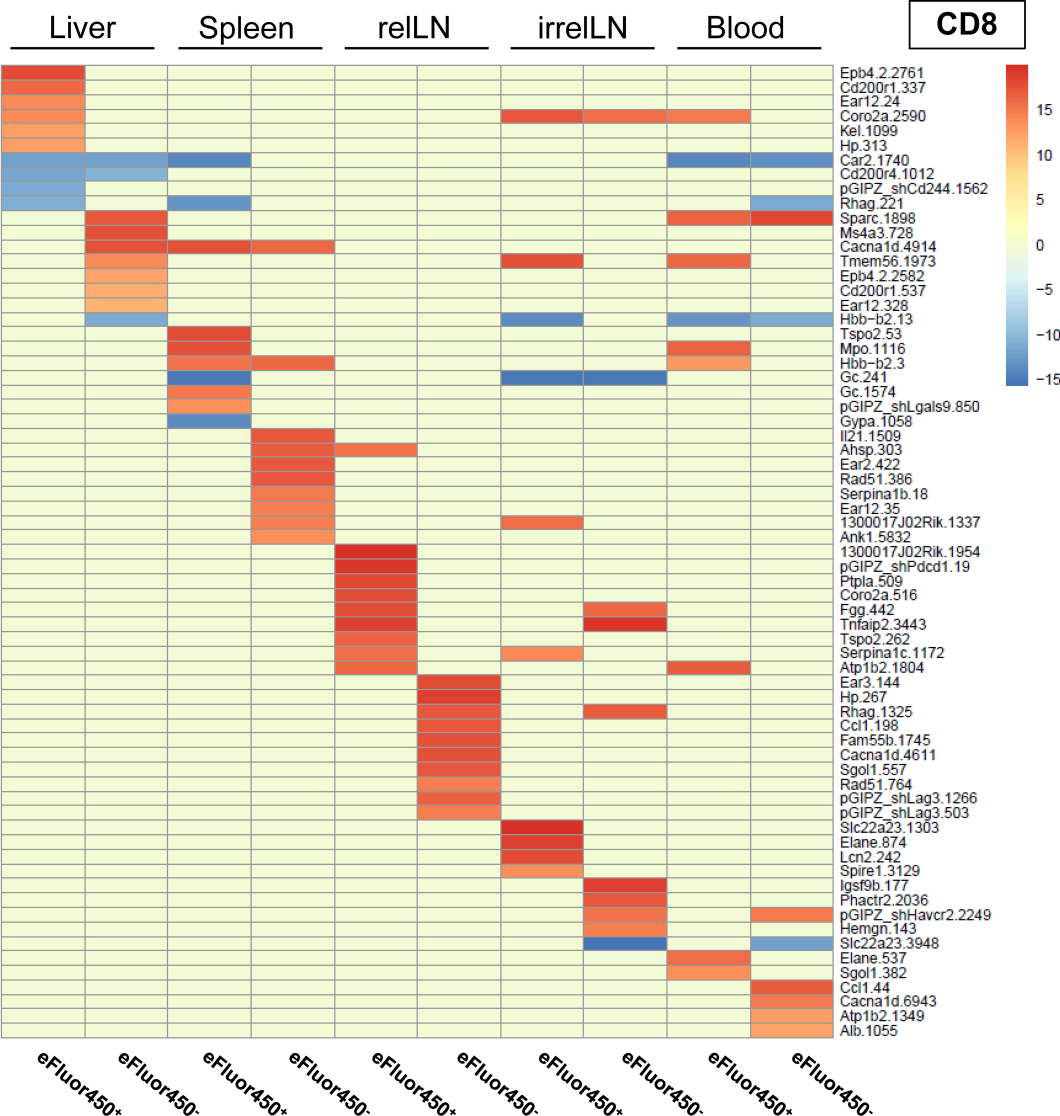
*In vivo* RNAi screen revealed several enriched shRNAs in CD8 T cells during HCC development. RNAi screen analysis was performed on eFluor450^-^ and eFluor450^+^ CD8 T cells isolated five days post-adoptive transfer from liver, spleen, relLN, irrelLN, and blood (out-probes) of HCC-bearing mice (*NRAS^G12V^/c-Myc* genotype). The CD8 T cells in out-probes were compared to the CD8 T cells before the adoptive transfer (in-probes). ShRNA enrichment (log2-fold changes) detected in isolated CD8 T cells of HCC-bearing recipient mice (out-probes) compared to CD8 T cells before the adoptive transfer (in-probes) are depicted in a heatmap with upregulated (>0, marked in red) and downregulated shRNAs (<0, marked in blue). Data represent a pooled analysis from four recipient mice. relLN, relevant lymph nodes; irrelLN, irrelevant lymph nodes.

To ensure reproducibility, four individual mice received shRNA-transduced CD4 and CD8 T cells. The sequencing results were pooled, and the 20 most enriched shRNAs targeting inhibitory genes for both CD4 and CD8 T cell subsets were identified (**Figures 3** and **4**). Data from individual mice are presented in **Supplementary Figure S5-12**.

In general, only a limited number of enriched shRNAs were identified in CD4 T cells, primarily originating from spleen (3 shRNAs), relLN (6 shRNAs), and blood (4 shRNAs) (**Figure 3**). Interestingly, these shRNAs were found in eFluor450^-^ CD4 T cells (**Figure 3**). In contrast to the pooled data on CD4 T cells (**Figure 3**), individual mouse analysis revealed a presence of many more enriched shRNAs on CD4 T cells in each of the explanted organs. Those shRNAs were detected in both eFluor450^-^ and eFluor450^+^ CD4 T cells (**Supplementary Figure S5**, **Supplementary Figure S7**, **Supplementary Figure S9**, **Supplementary Figure S11**).

In contrast to pooled data from CD4 T cells, a shRNA enrichment was observed in all analyzed organs in CD8 T cells (**Figure 4**), especially in CD8 T cells isolated from relLN with different shRNAs enriched in eFluor450^-^ and eFluor450^+^ CD8 T cells (**Figure 4**). Enriched shRNAs in CD8 T cells were also present in all organs of the individual mice showing a constant strong enrichment in relLN (**Supplementary Figure S6**, **Supplementary Figure S8**, **Supplementary Figure S10**, **Supplementary Figure S12**).

Importantly, among the enriched shRNAs in the pooled analysis of both, CD4 and CD8 T cell populations, several shRNAs were present and targeted prominent genes, such as: CD200 receptor 1 (*CD200r1*), calcium channel, voltage-dependent, L type, alpha 1D subunit (*Cacnad1*), and translocator protein (*Tspo*), which were found in T cells isolated from liver and/or relLN, the local organs of HCC development (**Figures 3**, **4**).

When comparing the RNAi screen data with the microarray results (comparing enriched genes from the microarray with enriched shRNAs targeting the same genes), we found that several genes previously identified as highly upregulated, including *Ngp, Hbb-b1, Hba-a1*, and *S100a8*, were consistently detected in individual mice in the RNAi screen via enriched shRNAs targeting these genes (**Figure 1** and **Supplementary Figure S5-12**, respectively). Based on these findings, we selected these four target genes for *in vivo* validation studies to further investigate their role as potential inhibitory regulators of T cell function in HCC.

### 3.4 Validation of potential targets *in vitro* using a specific shRNA-based knockdown of endogenous mRNA expression

To perform validation studies, we first defined the most potent shRNAs to efficiently knockdown the selected target genes (*Ngp*, *Hbb-b1, Hba-a1, S100a8*). The knockdown efficiency of each shRNA was tested in T cells isolated from HCC-bearing mice. To achieve this, we first induced HCC (genotype: *NRAS^G12V^/c-Myc*). Upon HCC development, mice were sacrificed, and CD4 and CD8 T cells were sorted from the liver, relLN, irrelLN, and spleen. T cells were then stimulated *in vitro* for three days with anti-CD3, anti-CD28, and IL-2. In parallel, HEK293T packaging cells, which were used as lentivirus-producer cells, were transfected with pGIPZ lentiviral vector system expressing a shRNA of interest. ShRen served as a control. Freshly produced lentiviral particles expressing a shRNA of interest and a GFP reporter were harvested after two days. CD4 and CD8 T cells were transduced with GFP-expressing lentiviral particles and on day three post-transduction, alive and GFP^+^ were isolated using cell sorting. The gating strategy used for sorting is depicted in **Supplementary Figure S3A, B**. Isolated GFP^+^ CD4 and GFP^+^ CD8 T cells were subjected to RNA isolation. The efficiency of shRNA-mediated knockdown was assessed using quantitative polymerase chain reaction (qPCR). Two representative *in vitro* knockdown examples are shown in **Supplementary Figure S13A-B**. The qPCR results showed that in comparison to control shRen, shNgp.140, and shNgp.452 demonstrated the highest knockdown in CD4 and CD8 T cells (**Supplementary Figure S13A**). Similarly, the most potent shRNA was defined in CD4, and CD8 T cells for *Hba-a1* (**Supplementary Figure S13B**), for *Hbb-b1* (data not shown), and *S100a8* (data not shown).

Based on the obtained data, the most efficient shRNA was selected for each of the target genes *Ngp* (shNgp.140), *Hbb-b1* (shHbb-b1.541), Hba-a1 (shHba-a1.122), and S100a8 (shS100a8), accordingly. The selected shRNAs were further tested in *in vivo* validation experiments, as described in the next section.

### 3.5 Experimental design for the *in vivo* validation of shRNA-mediated knockdown

In the next step of our study, we performed *in vivo* validation experiments to investigate the efficacy of shRNA-mediated knockdown of the selected target genes (*Ngp, Hbb-b1, Hba-a1, S100a8*) on CD4 and CD8 T cells function using several therapeutic regimes and different readouts. The experimental layout is described in **Supplementary Figure S13C**.

#### 3.5.1 T cell isolation, transduction, and sorting

CD4 and CD8 T cells were first isolated from HCC-bearing donor mice, and transduced with shRNA-expressing lentiviral particles, followed by sorting of GFP^+^ transduced T cells, as described previously (**Figure 2**).

#### 3.5.2 Adoptive transfer and therapeutic intervention

To evaluate the therapeutic effect of shRNA-mediated knockdown of a target gene, we used C57BL/6J recipient mice harboring HCCs (genotype: *NRAS^G12V^/c-Myc*, **Supplementary Figure S13C**). According to previous survival studies (data not shown), we defined the optimal time for the therapeutic intervention (adoptive transfer of modified shRNA-transduced CD4 and CD8 T cells) at two weeks post-HDI (**Supplementary Figure S13C**). Importantly, to achieve a comparable HCC stage in recipient animals, we selected those age- and gender-matched HCC-harboring animals which showed similar values in the classical diagnostic parameters for HCC and other liver diseases used in the clinic (26): aspartate aminotransferase (AST), alanine aminotransferase (ALT) and lactate dehydrogenase (LDH).

The therapy, comprising adoptively transferred T cells with shRNA-mediated knockdown of a target gene was applied once or twice in a weekly interval. For the adoptive transfer, transduced and previously pooled CD4 and CD8 T cells (approximately 2x10^5^ cells each) expressing shRNA of interest were administered to mice *i.v.* (**Supplementary Figure S13C**). Mice receiving T cells transduced with shRen served as a control (**Supplementary Figure S13C**).

To evaluate the impact of shRNA-mediated knockdown on the HCC progression, we systematically monitored several parameters in recipient mice: survival, body weight changes, liver inflammation using biochemical parameters, and expression of ICIs on CD4 and CD8 T cells.

### 3.6 *In vivo* knockdown of Ngp on CD4 and CD8 T cells significantly prolonged the survival of HCC-bearing mice while decreasing liver biochemical parameters

**Figure 5:**
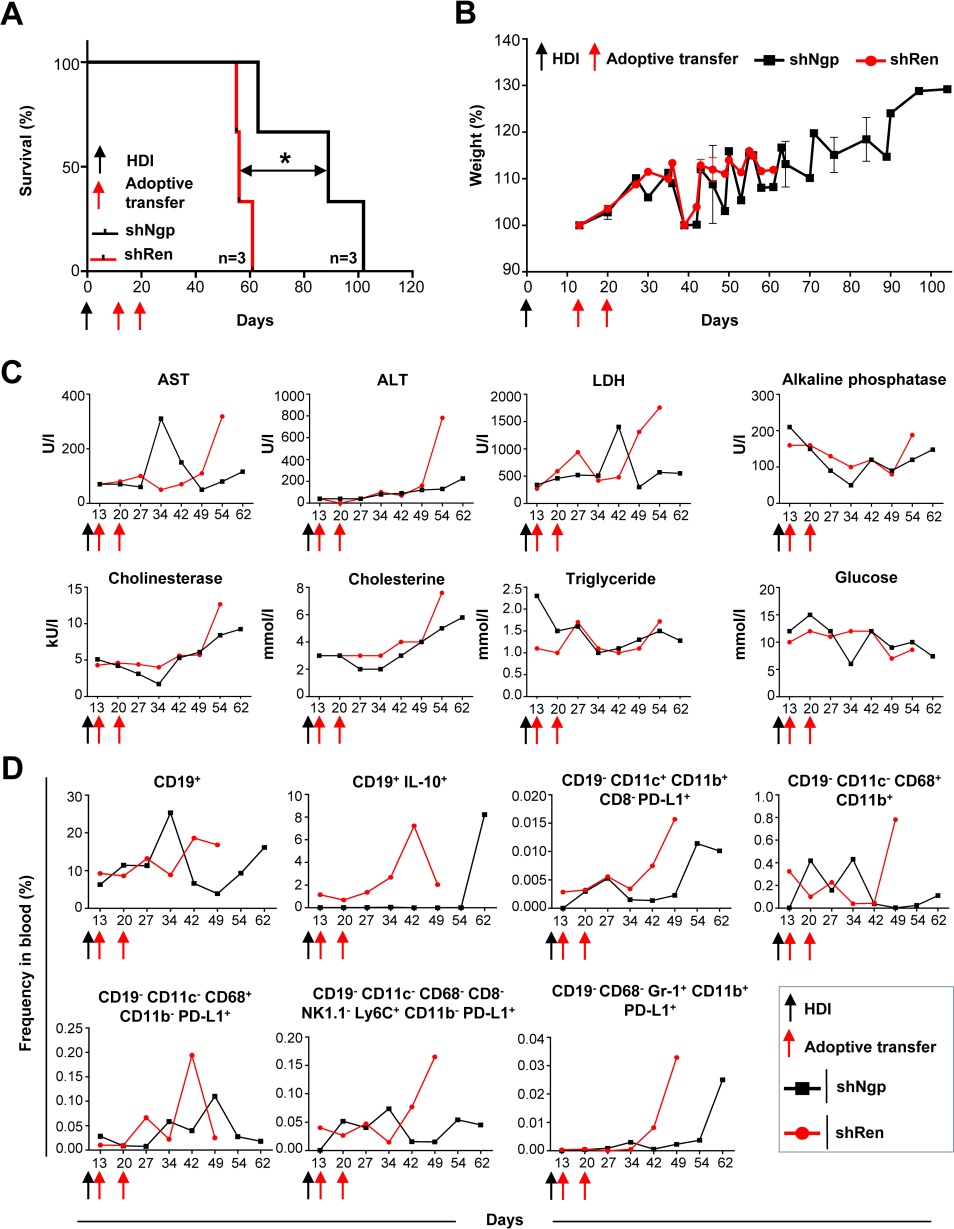
*Ngp* knockdown prolonged the survival of HCC-bearing recipient mice by decreasing biochemical parameters, CD19^+^ IL-10^+^ B cells, PD-L1-expressing monocytes, macrophages, and DCs in blood. **(A)** Kaplan-Meier survival curve (n=3 in each group). **(B)** Weight development (n=3 in each group). **(C)** Biochemical parameters in plasma (depicted are the data obtained in individual mice, as representative examples of the group). **(D)** Flow cytometry data on innate immune cells and B lymphocytes in blood (depicted are the data obtained in individual mice, as representative examples of the group). HDI, hydrodynamic tail vein injection; AST, alanine aminotransferase; ALT, aspartate aminotransferase; LDH, lactate dehydrogenase.

First, we aimed to validate *Ngp* as a potent T cell inhibitor, by using shNgp.140 (designated as shNgp) for *in vivo* knockdown. Prior to the adoptive transfer of shNgp-transduced T cells into C57BL/6J mice harboring HCC (genotype: *NRAS^G12V^/c-Myc*), we assessed the levels of the diagnostic parameters AST, ALT, and LDH in plasma of HCC-bearing recipient mice (genotype: *NRAS^G12V^/c-Myc*) (**Supplementary Figure S13D**) and selected comparable individuals as described above. On day 13 and 20 post-HDI, shNgp-transduced CD4 and CD8 T cells or shRen-transduced controls were adoptively transferred to the recipients. ShNgp-mediated knockdown in CD4 and CD8 T cells significantly prolonged the survival of HCC-bearing mice by 41 days compared to the shRen group (**Figure 5A**). We neither observed any impact on the body weight upon T cell transfer in both recipient groups (**Figure 5B**), nor did we detect any differences in liver tumor burden between shRen and shNgp groups at sampling, when reaching the termination criteria due to HCC development (**Supplementary Figure S13E**).

Over the course of the survival study, several biochemical parameters in the plasma of recipient mice were monitored to exclude toxic effects on liver metabolism caused by T cell therapy (**Figure 5C**, 2 representative mice are depicted). On day 34 post-HDI, we observed a six-fold increase of AST in the shNgp group which decreased by day 42 post-HDI (**Figure 5C**). At all other time points tested, including sampling, the AST level was lower in the shNgp group than in the shRen control group (**Figure 5C**). The ALT level showed to be constantly low in both groups, shNgp, and shRen, until day 54 post-HDI, when a dramatic increase was detected in the shRen group, which correlated with the advanced HCC development (**Figure 5C**). The kinetic of LDH was similar to AST (**Figure 5C**). Except for a threefold increase in shNgp group on day 42 post-HDI, LDH levels were lower in the shNgp recipient throughout the entire duration of the experiment, compared to the shRen control group (**Figure 5C**). Additional metabolic parameters including alkaline phosphatase, cholinesterase, cholesterine, triglyceride, and glucose also tended to be lower in the shNgp group compared to the shRen control (**Figure 5C**).

In summary, our results demonstrated a significant extension of survival in HCC-bearing mice, along with improvements in liver biochemical markers, suggesting a beneficial impact of targeting *Ngp* in T cell therapy.

### 3.7 *In vivo* knockdown of *Ngp* resulted in the reduction of PD-L1^+^ dendritic cells (DCs), macrophages, and IL-10^+^ B cells in HCC-bearing mice

To assess the efficacy of *Ngp* knockdown on immune cell dynamics, we collected blood samples from the retro-orbital plexus of recipient mice and checked for potential changes in the frequency of innate immune cells and B cells over time (**Figure 5D**, gating strategy in **Supplementary Figure S14**).

The frequency of CD19^+^ B cells remained similar between the shNgp and shRen groups until day 27 post-HDI (**Figure 5D**). However, in the shNgp group, CD19^+^ B cells dramatically increased on day 34, followed by a decline on day 42 post-HDI (**Figure 5D**). In contrast, in the shRen group, CD19^+^ B cells continued increasing on days 42 and 49 post-HDI, correlating with the aggressive HCC progression and recipien´s death (**Figure 5D**). Furthermore, shNgp-mediated knockdown led to a decreased level of immunosuppressive CD19^+^ IL-10^+^ B cells in comparison to the shRen control group (**Figure 5D**).

The *Ngp* knockdown also influenced DCs and macrophages. The proportion of PD-L1-expressing DCs (CD19^-^ CD11c^+^ CD11b^+^ CD8^-^ PD-L1^+^) was consistently lower in the shNgp group compaired to shRen group (**Figure 5D**). The macrophage (CD19^-^ CD11c^-^ CD68^+^ CD11b^+^) population showed fluctuations between the shNgp and shRen groups in the first 34 days post-HDI with a dramatic increase in shRen group and a moderate decrease in shNgp group when HCC developed and animals had to be sampled (**Figure 5D**). The counts for macrophages expressing PD-L1^+^ (CD19^-^ CD11c^-^ CD68^+^ CD11b^-^ PD-L1^+^) were, in general, lower in the shNgp group (**Figure 5D**). Similar counts of monocytes (CD19^-^ CD11c^-^ CD68^-^ CD8^-^ NK1.1^-^ Ly6C^+^ CD11b^-^ PD-L1^+^) were observed in both groups until day 27 post-HDI with a dramatic increase from day 42 post-HDI in the shRen and a decrease in the shNgp group (**Figure 5D**). The frequency of neutrophils (CD19^-^ CD68^-^ Gr-1^+^ CD11b^+^ PD-L1^+^) was comparable low and started to increase due to HCC development on day 42 and 62 in the shRen and shNgp groups, respectively (**Figure 5D**).

In summary, *Ngp* knockdown reduced immunosuppressive PD-L1^+^ DCs, macrophages, and IL-10^+^ B cells in HCC-bearing mice.

### 3.8 *In vivo* knockdown of *Ngp* controlled the expression of classical ICI molecules and reduced Tregs in HCC-bearing mice

**Figure 6:**
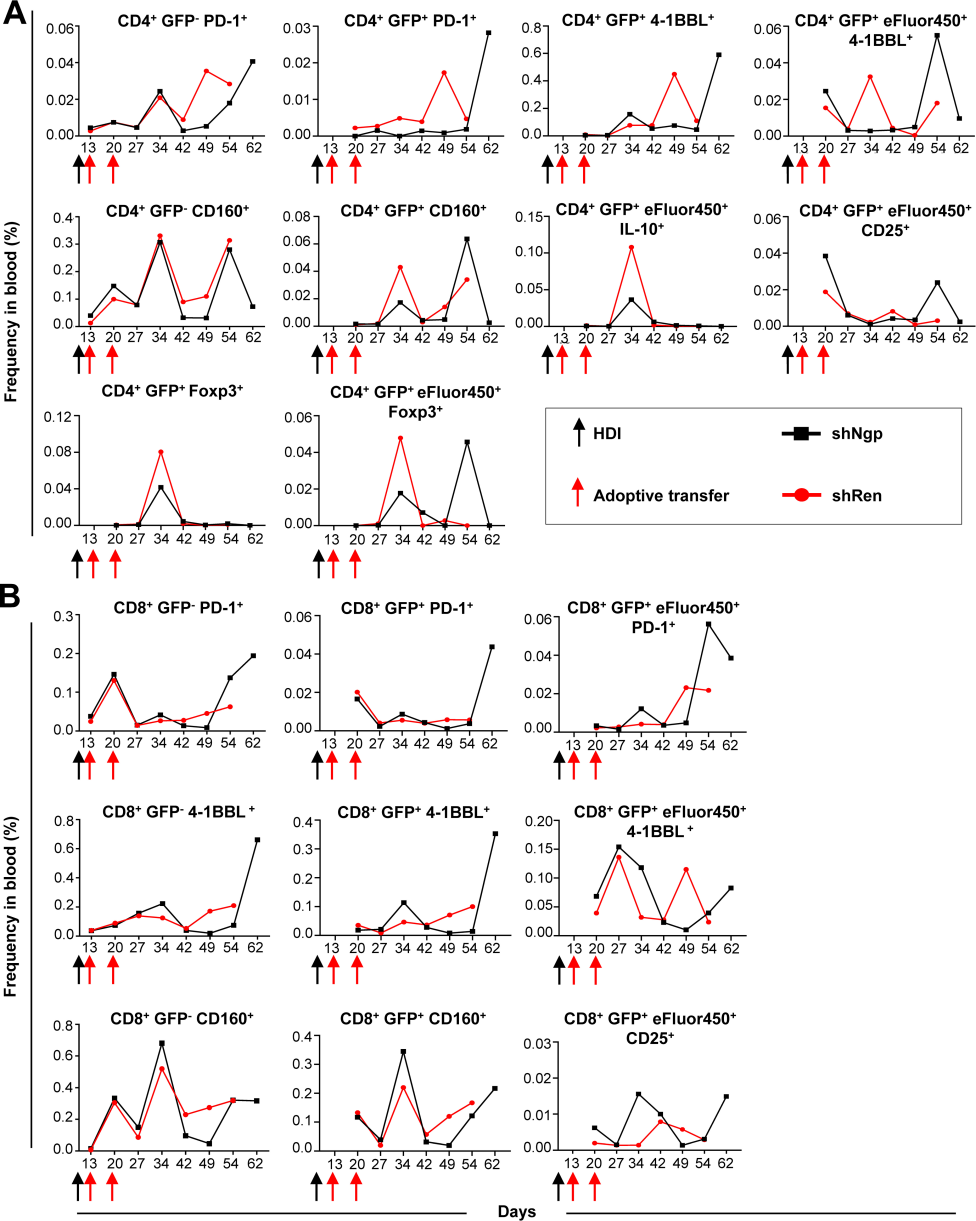
*Ngp* knockdown decreased the expression of PD-1, 4-1BBL, and CD160 ICIs on endogenous and exogenous CD4 T cells and increased CD25 expression on CD4 and CD8 T cells in blood. **(A, B)** Flow cytometry kinetic data of endogenous and exogenous **(A)** CD4 and **(B)** CD8 T cells in the blood of shNgp and shRen recipient mice, monitoring the expression of activation and inhibition markers upon adoptive transfer (depicted are the data obtained in individual mice, as representative examples of the group). HDI, hydrodynamic tail vein injection; GFP, green fluorescent protein.

To assess the impact of *Ngp* knockdown on T cell activation and classical ICIs repertoire expression, we analyzed both endogenous (GFP^-^) and adoptively transferred (exogenous, GFP^+^) CD4 and CD8 T cells while tracking their proliferation using eFluor450 labeling (**Figure 6**, **Supplementary Figure S15**).

We found that the PD-1 expression on endogenous GFP^-^ CD4 T cells was similar between both groups until day 34 post-HDI followed by a strong increase from day 49 post-HDI in the shRen group and day 64 post-HDI in the shNgp group (**Figure 6A**). Surprisingly, exogenous GFP^+^ CD4 T cells in the shNgp group showed a controlled, low level of PD-1 expression until the day of sampling, where a dramatic PD-1 peak was detected (**Figure 6A**).

Contrary results were observed between both groups regarding the expression of 4-1BBL on exogenous CD4 T cells (CD4^+^ GFP^+^ 4-1BBL^+^) (**Figure 6A**). While the 4-1BBL expression on exogenous CD4 T cells in the shRen group dramatically dropped on the day of sampling, a strong increase of 4-1BBL was found on exogenous CD4 T cells in the shNgp group at the time of HCC development (**Figure 6A**). The latter effect was observed *vice versa* on CD4^+^ GFP^+^ eFluor450^+^ 4-1BBL^+^ cells (**Figure 6A**).

We tested a further ICI molecule CD160 in our analysis: the treatment with shNgp and shRen had only a minor effect on CD160 expression level between both groups as shown by comparable curve pattern and frequency counts (**Figure 6A**). The expression of CD160 on exogenous CD4 T cells (CD4^+^ GFP^+^ CD160^+^) was more controlled in the shNgp group (**Figure 6A**).

Further analysis showed that exogenous CD4 T cells in the shNgp group showed lower IL-10 expression (CD4^+^ GFP^+^ eFluor450^+^ IL-10^+^) and a higher CD25 expression (CD4^+^ GFP^+^ eFluor450^+^ CD25^+^) compared to the shRen group (**Figure 6A**).

In addition, Tregs counts (CD4^+^ GFP^+^ Foxp3^+^/CD4^+^ GFP^+^ eFluor450^+^ Foxp3^+^) were lower in the shNgp group compared to the shRen group, indicating reduced immunosuppression (**Figure 6A**).

Similar to the PD-1 expression on endogenous CD4 T cells, CD8^+^ PD1^+^ T cells in both treatment groups showed until day 42 post-HDI comparable counts but were in contrast to data in CD4 T cells dramatically increasing in the shNgp treatment group 54 days post-HDI (**Figure 6B**). The frequency of exogenous CD8^+^ GFP^+^ PD-1^+^ cells in both groups was similar until day 54 post-HDI: here a strong increase of PD-1 in the shNgp group was detected and was similar to the finding on CD4^+^ GFP^+^ PD-1^+^ (**Figure 6B** and 6A, respectively). Also, a stronger expression of PD-1 in the CD8^+^ GFP^+^ eFluor450^+^ population was observed in the shNgp compared to the shRen group upon HCC development (**Figure 6B**). The expression pattern of 4-1BBL on endogenous CD8 T cells (CD8^+^ 4-1BBL^+^) resembled that on exogenous CD8 T cells (CD8^+^ GFP^+^ 4-1BBL^+^) showing both a strong increase of 4-1BBL on the day of sampling in the shNgp group but not in the shRen recipient (**Figure 6B**). Similar observations were made for CD160 on endogenous CD8 T cells (CD8^+^ GFP^-^ CD160^+^) and on exogenous CD8 T cells (CD8^+^ GFP^+^ CD160^+^) (**Figure 6B**).

Similar to CD4 T cells, the expression of CD25 on exogenous CD8 T cells (CD8^+^ GFP^+^ eFluor450^+^ CD25^+^) was higher in the shNgp group than in the shRen recipient (**Figure 6B**).

In summary, the *in vivo* knockdown of *Ngp* in CD4 and CD8 T cells controlled classical ICI molecules, including PD-1, 4-1BBL, and CD160, reduced Tregs and increased CD25 expression on the transduced T cells, suggesting an enhanced T cell activation and reduced immunosuppression in HCC-bearing mice.

### 3.9 *In vivo* knockdown of *Hbb-b1* moderately prolonged the survival of HCC-bearing mice

**Figure 7:**
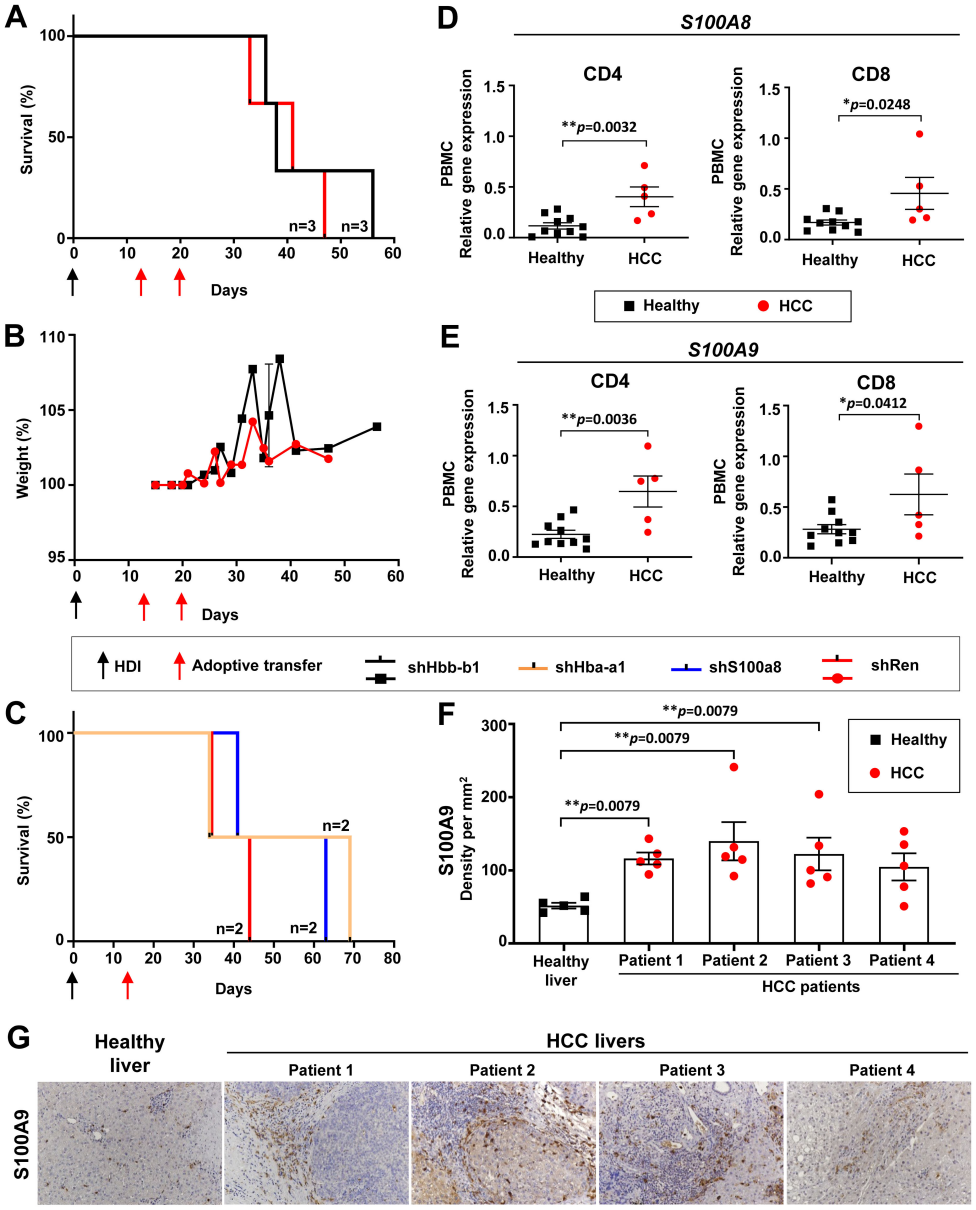
*Hba-a1* and *S100a8* knockdown prolonged the survival of HCC-bearing recipient mice and *S100A8* & *S100A9* were found upregulated in HCC patients. **(A)** Kaplan-Meier survival curve of mice that received two transfers of shHbb-b1-transduced CD4 and CD8 T cells or control (shRen) (n=3 in each group). **(B)** Weight development of mice that received two transfers of shHbb-b1-transduced CD4 and CD8 T cells or control (shRen) (n=3 in each group). **(C)** Kaplan-Meier survival curve of mice that received one transfer of shHba-a1- or shS100a8-transduced CD4 and CD8 T cells or control (shRen) (n=2 in each group). **(D, E)** CD4 T cells and CD8 T cells were isolated from PBMCs and liver tissues of HCC patients and healthy donors and analyzed for the expression of **(D)** *S100A8* and **(E)** *S100A9* using qPCR. **(G)** Representative images of IHC using anti-S100A9 staining in human HCCs (n=4) and healthy donor (n=1). Scale bar, 100 µm. **(F)** Cellular density of S100A9 positive cells in human HCCs (n=4) and healthy donor (n=1). HCC, hepatocellular carcinoma; HDI, hydrodynamic tail vein injection; *S100A8*, S100 calcium-binding protein A8; *S100A9*, S100 calcium-binding protein A9.

In a further validation study, we investigated the effect of *Hbb-b1* knockdown on HCC development (**Figures 7A, B**). Recipient groups were gender- and age-matched and selected according to similar AST, ALT, and LDH levels, as described in previous sections (data not shown). After the induction of HCC development in these mice, T cells were transferred on days 13 and 20 post-HDI (**Figure 7A**).

Upon *Hbb-b1* knockdown, we detected a moderate survival benefit of 11 days (**Figure 7A**). Upon T cell transfer, no toxicity was observed, as confirmed by a stable body weight in both experimental groups (**Figure 7B**). This was in line with previous experiments using shNgp (see section above and **Figure 5B**). No influence on the tumor burden was observed between the experimental and control group (data not shown).

In summary, *Hbb-b1* knockdown resulted in a moderate survival extension in HCC-bearing mice, with no observed toxicity or impact on tumor burden.

### 3.10 *In vivo* knockdown of *Hba-a1* and *S100a8* prolonged the survival of HCC-bearing mice, decreased liver biochemical parameters, PD-L1-expressing immune cells, and kept under control Tregs and several classical ICIs on T cells

We further tested an *in vivo* knockdown of *Hba-ba1* and *S100a8* using this time only a single transfer of T cells on day 13 post-HDI (**Figure 7C**). A single dose of shS100a8- and shHba-a1-transduced T cells led to a survival benefit of 19 and 25 days, respectively, in comparison to the shRen control group (**Figure 7C**). Next, we monitored the biochemical parameters in these mice. On day 27 post-HDI, we observed a decreased level of AST in the shS100a8- and shHba-a1 groups in comparison to shRen control mice, whereas ALT levels varied among the groups (**Supplementary Figure S16A**). The LDH level was highest in the shRen control group, moderately increased in the sh1008a8, and constantly low in the shHba-a1 group (**Supplementary Figure S16A**). The level of alkaline phosphatase was constantly low in the shS100a8- and shHba-a1 groups in comparison to the shRen control with a peak of this parameter on day 27 post-HDI (**Supplementary Figure S16A**). The levels of cholinesterase and cholesterine were similar among the groups, however, both parameters massively increased in mice with *S100a8* knockdown on day 68 post-HDI upon HCC development (**Supplementary Figure S16A**). The kinetic of triglyceride and glucose did not change in comparison to the shRen group (**Supplementary Figure S16A**).

Besides the survival study, we also monitored immune cells in the blood of recipient mice (**Supplementary Figure S16B**). Similar to the Ngp data, we observed lower numbers of CD19^+^ and CD19^+^ IL-10^+^ B cells in shHba-a1 and shS100a8 recipient mice, in comparison to the shRen control group (**Supplementary Figure S16B**). Furthermore, the *Hba-a1* and *S100a8* knockdown led to a constant lower level of PD-L1-expressing DCs and macrophages (CD19^-^ CD11c^+^ CD11b^+^ CD8^-^ PD-L1^+^ and CD19^-^ CD11c^-^ CD68^+^ CD11b^-^ PD-L1^+^, respectively) (**Supplementary Figure S16B**). The frequency of CD11b^+^ macrophages (CD19^-^ CD11c^-^ CD68^+^ CD11b^+^) was in general increased in the shHba-a1 and shS100a8 groups, similar to the shNgp group (**Supplementary Figure S16B** and **Figure 5D**). Similarly to the Ngp data, the counts for macrophages expressing PD-L1^+^ (CD19^-^ CD11c^-^ CD68^+^ CD11b^-^ PD-L1^+^) were in general lower in the shHba-a1 and shS100a8 groups (**Supplementary Figure S16B** and **Figure 5D**). A comparable pattern was observed in the monocyte population (CD19^-^ CD11c^-^ CD68^-^ CD8^-^ NK1.1^-^ Ly6C^+^ CD11b^-^ PD-L1^+^) in all groups (**Supplementary Figure S16B**). Despite a peak in the frequency of PD-L1^+^ neutrophils (CD19^-^ CD68^-^ Gr-1^+^ CD11b^+^ PD-L1^+^) on day 41 post-HDI, the shS100a8 group kept those cells in controlled lower levels in comparison to shRen and shHba-a1 groups (**Supplementary Figure S16B**).

Further analysis of CD4 T cells showed the constantly low PD-1 expression in endogenous CD4^+^ PD-1^+^ and exogenous GFP^+^ CD4^+^ PD-1^+^ T cells in the shHba-a1 and shS100a8 groups, in comparison to shRen control (**Supplementary Figure S17A**). As observed in the Ngp data, we detected an increase of exogenous CD4^+^ GFP^+^ 4-1BBL^+^ and CD4^+^ GFP^+^ eFluor450^+^ 4-1BBL^+^ T cells in shS100a8 and shRen groups (**Supplementary Figure S17A**). Interestingly, the *in vivo* knockdown of *Hba-a1* kept at the stable level not only PD-1 but also 4-1BBL expression despite the HCC development (**Supplementary Figure S17A**). Further analysis of CD4 T cells showed similar fluctuations in the expression of the CD160 molecule as detected in Ngp data set. Still, the expression of CD160 on endogenous CD4^+^ CD160^+^ as well as exogenous CD4 T cells (CD4^+^ GFP^+^ CD160^+^) was more controlled in shHba-a1 and shS100a8 groups when compared to the shRen control (**Supplementary Figure S17A**).

Exogenous CD4 T cells in the shHba-a1 and shS100a8 groups also showed a lower IL-10 (CD4^+^ GFP^+^ eFluor450^+^ IL-10^+^) and CD25 (CD4^+^ GFP^+^ eFluor450^+^ CD25^+^) expression compared to the shRen control group (**Supplementary Figure S17A**). Importantly, Tregs counts (CD4^+^ GFP^+^ Foxp3^+^/CD4^+^ GFP^+^ eFluor450^+^ Foxp3^+^) remained low and well controlled in the shHba-a1 and shS100a8 groups, starting from day 44 until HCC development, in contrast to the shRen control group (**Supplementary Figure S17A**).

We further analyzed CD8 T cells and detected that the shS100a8 group showed a similar pattern of control of PD-1 and 4-1BBL expression, as detected in the shNgp group, whereas the shHba-a1 group fully controlled the expression of both molecules on CD8 T cells (**Supplementary Figure S17B** and **Supplementary Figure S8B**). Similar to CD4 T cells, the expression of CD160 on endogenous CD8 T cells (CD8^+^ CD160^+^) and on exogenous CD8 T cells (CD8^+^ GFP^+^ CD160^+^) was more controlled in shHba-a1 and shS100a8 groups in comparison to shRen control (**Supplementary Figure S17B**). Interestingly, CD25 expression in the shHba-a1 and shS100a8 groups was not as pronounced as in the shNgp group (**Supplementary Figure S17B** and **Figure 6B**).

In summary, the *in vivo* knockdown of *Hba-a1* and *S100a8* prolonged the survival of HCC-bearing mice already after one T cell transfer, thereby decreasing liver biochemical parameters, CD19^+^ IL-10^+^ B cells, PD-L1-expressing DCs, and macrophages as well as Tregs and inhibitory markers, like PD-1, CD160 and 4-1BBL, on T cells. *In vivo* knockdown of *Hba-a1* was especially efficient while leading to a constantly low expression of inhibitory markers on both, CD4 and CD8 T cells.

### 3.11 T cell therapy and *in vivo* knockdown of *Ngp*, *Hbb-b1, Hba-a1*, and *S100a8* was safe as confirmed by histopathological analyses of different murine organs

To exclude organ pathology upon T cell therapy, we isolated different organs from all mice in validation studies. Importantly, we could not detect any significant histopathological changes in any of tested groups after T cell transfer (**Supplementary Figure S18**). The brain, heart, lungs, kidneys, pancreas, spleen preserved their histoarchitecture and cellular structure as confirmed by an experienced pathologist (**Supplementary Figure S18**). As expected, microscopical examination of liver tissues revealed the presence of multiple neoplastic nodules composed of pleomorphic cells in all experimental groups, consistent with HCC development (**Supplementary Figure S18**). However, no additional pathological alterations were detected in liver tissues beyond those associated with tumor progression, further supporting the safety of the administered T cell therapy and gene knockdown approaches.

### 3.12 Upregulation of neutrophil-associated proteins and significant increase in expression of *S100A8* and *S100A9* genes in CD4 and CD8 T cells in PBMCs of HCC patients

To extrapolate the data obtained in mice to humans, we analyzed patients-derived material for the expression of the newly identified target genes. For these purposes, CD4 and CD8 T lymphocytes were isolated and sorted from HCC tumor tissue, HCC-free tissue areas, and healthy liver tissue, as well as from the peripheral blood mononuclear cells (PBMCs) of HCC patients and healthy donors. qPCR analysis was performed to define the expression of target genes in human CD4 and CD8 T cells (**Figures 7D, E**, **Supplementary Figure S19**). As *Ngp* encodes a mouse-specific neutrophil-associated protein (27) not present in humans, we analyzed cathelicidin (*CAMP*), the closest orthologue of mouse *Ngp* (28). Surprisingly, we could not confirm an upregulation of CAMP on PBMCs isolated from HCC patients in comparison to healthy controls (**Supplementary Figure S19A**).

Next, we investigated the expression of *HBB*, the human orthologue to mouse-specific *Hbb-b1*, in the PBMCs of HCC patients. We could not identify any differences in *HBB* expression between PBMCs from HCC patients and healthy donors (**Supplementary Figure S19B**). We compared further prominent and highly upregulated neutrophil-associated genes, *lipocalin2* (*Lcn2*), *S100a8*, and *S100a9*, identified as potential new targets in the microarray data (27). Interestingly, we detected lower expression levels of *LCN2* in CD4 and CD8 T cells in PBMCs from HCC patients (**Supplementary Figure S19C**). However, in T cells isolated from HCC liver tissues (HCC-free and HCC tissue areas), the opposite effect (not significant) was observed as *LCN2* expression was increased compared to healthy controls (**Supplementary Figure S19D**).

Finally, we analyzed the expression of *S100A8* and *S100A9* and found a significant increase of both genes in CD4 and CD8 T cells in PBMCs of HCC patients compared to healthy controls (**Figures 7D–F**).

Importantly, too low numbers of CD4 and CD8 T cells were obtained from resected HCC and healthy liver tissues, and thus, RNA thereof was a limiting factor in these experiments. Due to the limited amount of material, we could not perform further qPCR analysis on T lymphocytes isolated from liver tissue for other targets. We, therefore, continued checking *S100A8/S100A9* molecule in human paraffin liver sections (see next section).

### 3.13 Immunohistochemistry analysis revealed increased S100A9-expressing immune cell infiltration in human HCC

IHC analysis of selected HCC patients revealed a strong infiltration of tumor stroma and parenchyma with S100A9-expressing immune cells in comparison to healthy control (**Figures 7F, G**), as defined by an experienced pathologist. The cellular density of S100A9-expressing immune cells in the HCC tumor core in four tested HCC patients was significantly increased compared to healthy control samples (**Figure 7F**).

## 4 Discussion

In the present study, we aimed to identify new inhibitory targets, novel ICIs, on T lymphocytes that are triggered by the HCC TME and are subsequently leading to the inactivation of T cell function in patients with HCC.

We first worked in mice and performed a microarray analysis on T lymphocytes isolated from autochthonous HCC murine models, which highly reflect human disease (21–23, 25, 29, 30). Based on our results, we identified several upregulated genes (72 genes) in memory (CD44^+^) (31) CD4 and CD8 T lymphocytes in HCC-bearing mice, which were further validated using the shRNA library and the RNAi screen.

The RNAi screen study was performed in four individually treated mice and for the analysis we used pooled data from all four mice and data obtained from individual animals were additionally considered. Surprisingly, only a few shRNAs enrichments were found on CD4 T cells (liver, relLN, blood), whereas shRNAs in CD8 T cells were found enriched in all the analyzed organs. Among the obtained data, we found that shRNAs were targeting prominent target genes such as: *CD200r1, Tspo*, and *Cacna1d*, which were detected in the liver and relLN of HCC-bearing mice. In humans, *CD200R1* is mainly expressed on myeloid-derived and lymphoid-derived immune competent cells (32) and the interaction with its ligand *CD200R1* was reported to promote relapse of rectal cancer (33), to be involved in HCC progression (34) and high expression of *CD200R1* was associated with poor prognosis in non-small cell lung cancer (35). In humans, *TSPO* was reported to be involved in the regulation of cellular proliferation, apoptosis, and mitochondrial functions (36). Further, *TSPO* was found up-regulated in colorectal and breast cancer, where it promotes the malignancy of aberrant cells (37, 38). *CACNA1D* belongs to the family of Voltage-gated calcium channels, which play a role in cellular functions including mitogenesis, proliferation, differentiation, apoptosis, and metastasis (39). High expression of *CACNA1D* correlated with various types of cancer (40).

For the validation studies, we focused on *Ngp*, *Hbb-b1*, *Hba-a1*, and *S100a8*, which were highly upregulated in the microarray analysis and T cells, harboring shRNAs which targeted these genes, were present at higher frequencies in RNAi screen data of individual mice.

The closest human orthologue to *Ngp* is the anti-microbial peptide *CAMP* (28). Neutrophils contain several abundant anti-microbial proteins and some of those proteins are shared between mice and humans, such as LCN2, cathepsin G (CTSG), myeloperoxidase, and S100A8/A9 (27, 41). Importantly, *Lcn2, S100a8, and S100a9* were also found to be highly upregulated in HCC liver, relLN, and spleen in the microarray and screen studies (individual mouse analysis). Interestingly, no expression of *Ngp* on T lymphocytes could be detected to date and it seems that our data demonstrated the involvement of *Ngp* (its upregulation) in T cells in murine HCC for the first time. However, the overexpression of *Ngp*´s orthologues in human CD4 and CD8 T cells in HCC remains to be investigated using patient-derived HCC tissues, as discussed below.

The *Hbb-b1* gene is one out of four subunits of hemoglobin, a protein in red blood cells, which is mediating the oxygen transport (42). The human orthologue *HBB* (42, 43), mainly present in erythrocytes, is also expressed in macrophages, epithelial cells, neurons, and hepatocytes (44). In liver cancer, low oxygen levels lead to hypoxic conditions, which in turn augment an increased availability of hemoglobin and other factors to provide tumor angiogenesis (42, 45). To the best of our knowledge, we are the first to show upregulation of *Hbb-b1* in T cells in HCC. However, its expression in the human HCC context remains to be elucidated, as discussed below.

The *Hba-a1* gene, which encodes the alpha 1 subunit of hemoglobin, is crucial for the oxygen transport (46). Hba-a1 is located in the myelin sheath and is expressed in different structures, including blood vessels, early conceptus, the hematopoietic system, liver, and visceral pericardium (47). Its human orthologue *HBA1* (48) is associated with different hemoglobin disorders (Heinz body anemia, alpha thalassemia, familial erythrocytosis 7, and hemoglobin H disease) (49). Interestingly, the knockdown of *Hba-a1* was more efficient and led to a better survival in our validation studies than knockdown of *Hbb-b1*. However, this is reported for the first time and requires follow-up studies with a deeper analysis of data with an increased group size and a direct comparison of both molecules.

S100A8 and S100A9 are two closely related proteins that belong to the S100 family of calcium-binding proteins (50). Both S100A8 and S100A9 play important roles in various cellular processes, including inflammation and immune response (51, 52). S100A8 and S100A9 are frequently expressed in neutrophils, macrophages, monocytes, and other immune cells (51, 53, 54). Among them, calprotectin is abundantly expressed in neutrophils, accounting for approximately 50% of cytoplasmic proteins (55, 56). In an inflammatory environment, S100A8 and S100A9 can be expressed in activated keratinocytes, epithelial cells, and osteoclasts (50, 52, 57). Most S100 family members, such as S100A8 and S100A9, have already been reported to be involved in liver cancer (50). Extracellular S100A9 enhanced the activation of the mitogen-activated protein kinase (MAPK) signaling pathway via combination with the receptor advanced glycation end-product (58, 59). Besides, both S100A8 and S100A9 were previously reported to be associated with HCC by promoting cell proliferation (60, 61).

In our study, the knockdown of *Ngp* in T cells resulted in a significant prolongation of survival of HCC-bearing mice upon two therapeutic T cell transfers (41 days). Whereas *Hbb-b1* (10 days) was not as effective. Furthermore, a single therapeutic transfer of shHba-a1 and shS100a8-transduced T cells showed a prolongation of survival (26 and 20 days, respectively).

Importantly, the adoptive T cell therapy comprising CD4 and CD8 T cells with either *Ngp, Hbb-b1*, *Hba-a1* or *S100a8* knockdown was well tolerated and showed no toxicity, weight loss or any other side effects. Currently, many clinical trials on HCC are investigating the safety of T cell receptor and chimeric antigen receptor T cell (CAR-T) therapy. Two studies reported an objective response (62, 63). Until today, four approved CAR-T cell therapies for the treatment of hematologic cancer are available and two further CAR-T cell therapies for multiple myeloma are on the way to approval as standard use (62, 63). Key limitation factors for the approval of T cell therapies in solid tumors are the accessibility of T cells in the complex tumor structure, high heterogeneity and the immunosuppressive TME, inducing up-regulation of ICIs and subsequent dysfunction of T cells. A limited expansion and persistence of transferred CAR-T cells were also reported and are considered as critical parameters to prevent from tumor recurrence (62, 63). In our study, a survival benefit upon *Ngp*, *Hba-a1*, and *S1008* knockdown in T cells in HCC was achieved and was accomplished by a reduction of PD-1 on CD4 T cells in the blood. This highlights the efficacy of T cell therapy to influence PD-1 expression without additional intervention, such as anti-PD-1 blocking antibodies, like e.g. Nivolumab which are approved by the U.S. Food and Drug Administration and are currently used for HCC treatment (64). Another benefit of *Ngp*, *Hba-a1*, and *S1008* knockdown was the reduction of Foxp3 T cells (Tregs), which are associated with poor survival prognosis in HCC patients (65). Interestingly, we observed similar patterns among shNgp and shS1008 groups regarding the control of expression of PD-1, 4-1BBL, and CD160 inhibitory molecules with a final increase of those on T cells upon HCC development. In contrast, in the shHba-a1 group, the expression of all three inhibitory molecules was kept mostly at constantly low levels. However, its efficacy needs to be further studied in follow-up studies.

The knockdown of *Ngp*, *Hba-a1*, and *S1008* also positively impacted innate immune cell populations by reducing PD-L1-expressing monocytes, DCs, and macrophages. In our previous studies, B cells were shown to be increased in HCC, suggesting a tumor-promoting role (24, 66). In line with this, we found IL-10^+^-expressing B cells to be decreased in groups treated with shNgp, shHba-a1, and shS1008-transduced T cells.

Another beneficial effect of T cell therapy could be observed on biochemical parameters in plasma such as AST, ALT, LDH, ALP, GDH, cholesterine, triglyceride, and glucose. These parameters belong to a standard clinical biochemical analysis of plasma which is used to detect hepatotoxicity in patients (67). In our study, the biochemical parameters AST, ALT, and LDH were kept at lower levels upon T cell therapy with the knockdown of *Ngp*, *Hba-a1*, and *S1008* in comparison to the control (shRen).

To extrapolate our observations into human/clinic, we identified human orthologues for the defined murine inhibitory target genes. Thereby, we could partially confirm our microarray data by showing upregulation of *S100A8* and *S100A9* in T cells and also in human samples using qPCR - both *S100A8* and *S100A9* were found significantly upregulated on CD4 and CD8 T cells isolated from blood PBMCs of HCC patients. Importantly, S100A9-positive cells were also upregulated in human HCC tissues derived from four patients.

In contrast to our expectation, *LCN2*, *CAMP*, and *HBB*, did not confirm our data obtained in mice and were not found upregulated in blood PBMCs isolated from HCC patients. Although *LCN2*, *CAMP*, and *HBB* are orthologues of mouse *Lcn2, Ngp*, and *Hbb-b1*, it was reported that there are divergences in the expression and the splicing of genes between human and mouse which might explain the observed discrepancies (68, 69).

It is also important to mention that T cells in our mouse study were isolated from tissues and predominantly compared to human T cells isolated from PBMCs. Different cell localization contains different composition of activation/inhibitory receptors in the surrounding compartment which shapes the phenotype of immune cells (70). In studies of Tada et al. on patients with advanced gastric cancer and VEGF-blocking antibody treatment, tumor-infiltrating lymphocytes (TILs) and PBMCs were compared regarding their expression of ICIs (PD-1, LAG3, CTLA-4, ICOS) and demonstrated a higher presence of ICIs on TILs than on PBMCs, highlighting the influence of T cell localization on the T cell phenotype (71). Performing single-cell sequencing of TILs from different tumors revealed that even within the same tumor different subtypes of T cells are present which differ from T cells in normal tissue (72). Also, it was mentioned that TILs found in one type of cancer, differ from those found in another type of cancer (72), which might be also influenced by the stage of the disease (72, 73). Therefore, to establish tools for phenotyping T cells as prognostic markers, sufficient characterizations are necessary and for treatments with immunomodulatory agents, the stages of disease and the T cell source have to be considered. Importantly and in line with the above mentioned, upregulation of *LCN2* which was not detected in PBMCs, although not significant, was shown in our study in human HCC tissues in CD4 and CD8 T cells. Therefore, it remains to be elucidated using patients-derived HCC tissues, whether *Ngp*, *Hbb-b1*, *Hba-a1*, and *S1008* orthologues are upregulated on T cells during HCC development. Also, further prominent genes identified in the screen (*CD200r1, Tspo, Cacna1d*) need to be validated in human material. While planning validation experiments, it is highly important to perform kinetic studies and to follow-up the changes in counts as well as ICIs phenotype in adoptively transferred as well as endogenous T cells. Our kinetic studies showed that T cell therapy influences also endogenous CD4 and CD8 T cells and that the expression pattern on transferred (exogenous) T cells mostly resembled the expression pattern of endogenous CD4 and CD8 T cells.

We performed validation studies for only four (*Ngp, Hbb-b1, Hba-a1, S100a8*) genes. Further studies on genes identified in the screen need to be performed and confirmed in human material, including a thorough mechanistic characterization. Also, CD4 and CD8 T cells should be applied *in vivo* separately to unravel the potential of shRNA for each T cell type individually. Further, a combination therapy, comprising i) “therapeutic” T cells with ii) antibodies targeting classical ICIs such as α-PD-1 and/or cancer vaccines based e.g. on attenuated *Listeria monocytogenes* as recently developed in our study (66), could be further considered, especially at advanced stages of this highly aggressive malignant liver disease.

## 5 Conclusions

In conclusion, in our study, we defined new inhibitory markers (ICI molecules) arising on memory T cells during aggressive HCC development. Employing a murine model that closely mimics human disease, we identified a repertoire of upregulated genes in CD4 and CD8 T lymphocytes, unveiling potential targets for therapeutic intervention.

The adoptive T cell therapy was safe and targeting *Ngp*, *Hba-a1*, and *S100a8* genes demonstrated a substantial survival benefit in aggressive murine HCC models. Moreover, this therapeutic approach exhibited efficacy in modulating immune checkpoints, such as PD-1, 4-1BBL, and CD160 on endogenous and exogenous (transferred) CD4 and CD8 T cells.

Beyond survival outcomes, our T cell therapy exerted positive effects on innate immune cell populations while reducing PD-L1 molecules on DCs, monocytes, and macrophages, decreasing IL-10^+^ B cells and controlling liver biochemical parameters, altogether offering a comprehensive perspective on its potential clinical applications. In addition, we identified the presence of at least one target (*S100A8/S100A9*) in human samples using qPCR analysis in PBMCs and via IHC on HCC liver tissues. The obtained results pave the way for the use of the defined molecules as important immunotherapeutic targets in further preclinical and clinical studies in HCC patients.

## Data Availability

The data presented in the study are deposited in the Gene Expression Omnibus (GEO) repository, under the link GEO Accession viewer, the accession number GSE144811.

## References

[1] Sung H Ferlay J Siegel RL Laversanne M Soerjomataram I Jemal A. Global cancer statistics 2020: globocan estimates of incidence and mortality worldwide for 36 cancers in 185 countries. CA: Cancer J Clin. (2021) 71:209–49. doi: 10.3322/caac.21660 33538338

[2] World Health Organization. Cancer (2021). Available online at: https://www.who.int/news-room/fact-sheets/detail/cancer (Accessed March 3, 2021). https://www.who.Int/News-Room/Fact-Sheets/Detail/Cancer.

[3] Llovet JM Kelley RK Villanueva A Singal AG Pikarsky E Roayaie S. Hepatocellular carcinoma. Nat Rev Dis Primers. (2021) 7:6. doi: 10.1038/s41572-020-00240-3 33479224 PMC13537433

[4] Singal AG Lampertico P Nahon P. Epidemiology and surveillance for hepatocellular carcinoma: new trends. J Hepatol. (2020) 72:250–61. doi: 10.1016/j.jhep.2019.08.025 PMC6986771 31954490

[5] Schwartz H Blacher E Amer M Livneh N Abramovitz L Klein A. Incipient melanoma brain metastases instigate astrogliosis and neuroinflammation. Cancer Res. (2016) 76:4359–71. doi: 10.1158/0008-5472.CAN-16-0485 27261506

[6] Kumari R Sahu MK Tripathy A Uthansingh K Behera M. Hepatocellular carcinoma treatment: hurdles, advances and prospects. Hepat Oncol. (2018) 5:HEP08. doi: 10.2217/hep-2018-0002 31293776 PMC6613045

[7] Celsa C Giuffrida P Stornello C Grova M Spatola F Rizzo GEM. Systemic therapies for hepatocellular carcinoma: the present and the future. Recenti Prog Med. (2021) 112:110–6. doi: 10.1701/3559.35371 33624623

[8] Llovet JM Ricci S Mazzaferro V Hilgard P Gane E Blanc JF. Sorafenib in advanced hepatocellular carcinoma. N Engl J Med. (2008) 359:378–90. doi: 10.1056/NEJMoa0708857 18650514

[9] Finn RS Qin S Ikeda M Galle PR Ducreux M Kim TY. Atezolizumab plus bevacizumab in unresectabl e hepatocellular carcinoma. New Engl J Med. (2020) 382:1894–905. doi: 10.1056/NEJMoa1915745 32402160

[10] Cassetta L Bruderek K Skrzeczynska-Moncznik J Osiecka O Hu X Rundgren IM. Differential expansion of circulating human mdsc subsets in patients with cancer, infection and inflammation. J Immunother Cancer. (2020) 8:e001223. doi: 10.1136/jitc-2020-001223 32907925 PMC7481096

[11] Greten TF Manns MP Korangy F. Immunotherapy of hcc. Rev Recent Clin Trials. (2008) 3:31–9. doi: 10.2174/157488708783330549 18474013

[12] Korangy F Hochst B Manns MP Greten TF. Immunotherapy of hepatocellular carcinoma. Expert Rev Gastroenterol Hepatol. (2010) 4:345–53. doi: 10.1586/egh.10.18 20528121

[13] Breous E Thimme R. Potential of immunotherapy for hepatocellular carcinoma. J Hepatol. (2011) 54:830–4. doi: 10.1016/j.jhep.2010.10.013 21145836

[14] Ding W Xu X Qian Y Xue W Wang Y Du J. Prognostic value of tumor-infiltrating lymphocytes in hepatocellular carcinoma: A meta-analysis. Med (Baltimore). (2018) 97:e13301. doi: 10.1097/MD.0000000000013301 PMC6320107 30557978

[15] Wada Y Nakashima O Kutami R Yamamoto O Kojiro M. Clinicopathological study on hepatocellular carcinoma with lymphocytic infiltration. Hepatology. (1998) 27:407–14. doi: 10.1002/hep.510270214 9462638

[16] Chew V Tow C Teo M Wong HL Chan J Gehring A. Inflammatory tumour microenvironment is associated with superior survival in hepatocellular carcinoma patients. J Hepatol. (2010) 52:370–9. doi: 10.1016/j.jhep.2009.07.013 19720422

[17] Marin-Acevedo JA Dholaria B Soyano AE Knutson KL Chumsri S Lou Y. Next generation of immune checkpoint therapy in cancer: new developments and challenges. J Hematol Oncol. (2018) 11:39. doi: 10.1186/s13045-018-0582-8 29544515 PMC5856308

[18] Qin W Cao Z-Y Liu S-Y Xu X-D. Recent advances regarding tumor microenvironment and immunotherapy in hepatocellular carcinoma. Hepatoma Res. (2020) 6:24. doi: 10.20517/2394-5079.2020.04

[19] Mohr R Jost-Brinkmann F Ozdirik B Lambrecht J Hammerich L Loosen SH. Lessons from immune checkpoint inhibitor trials in hepatocellular carcinoma. Front Immunol. (2021) 12:652172. doi: 10.3389/fimmu.2021.652172 33859646 PMC8042255

[20] Federico P Petrillo A Giordano P Bosso D Fabbrocini A Ottaviano M. Immune checkpoint inhibitors in hepatocellular carcinoma: current status and novel perspectives. Cancers (Basel). (2020) 12:3025. doi: 10.3390/cancers12103025 33080958 PMC7603151

[21] Kang TW Yevsa T Woller N Hoenicke L Wuestefeld T Dauch D. Senescence surveillance of pre-malignant hepatocytes limits liver cancer development. Nature. (2011) 479:547–51. doi: 10.1038/nature10599 22080947

[22] Eggert T Wolter K Ji J Ma C Yevsa T Klotz S. Distinct functions of senescence-associated immune responses in liver tumor surveillance and tumor progression. Cancer Cell. (2016) 30:533–47. doi: 10.1016/j.ccell.2016.09.003 PMC7789819 27728804

[23] Petriv N Neubert L Vatashchuk M Timrott K Suo H Hochnadel I. Increase of alpha-dicarbonyls in liver and receptor for advanced glycation end products on immune cells are linked to nonalcoholic fatty liver disease and liver cancer. Oncoimmunology. (2021) 10:1874159. doi: 10.1080/2162402X.2021.1874159 33628620 PMC7889131

[24] Petriv N Suo H Hochnadel I Timrott K Bondarenko N Neubert L. Essential roles of B-cell subsets in the progression of MASLD and HCC. JHEP Rep. (2024) 11:101189. doi: 10.1016/j.jhepr.2024.101189 PMC11602976 39611128

[25] Dauch D Rudalska R Cossa G Nault JC Kang TW Wuestefeld T. A myc-aurora kinase a protein complex represents an actionable drug target in P53-altered liver cancer. Nat Med. (2016) 22:744–53. doi: 10.1038/nm.4107 27213815

[26] Anstee QM Reeves HL Kotsiliti E Govaere O Heikenwalder M. From nash to hcc: current concepts and future challenges. Nat Rev Gastroenterol Hepatol. (2019) 16:411–28. doi: 10.1038/s41575-019-0145-7 31028350

[27] Cassatella MA Ostberg NK Tamassia N Soehnlein O. Biological roles of neutrophil-derived granule proteins and cytokines. Trends Immunol. (2019) 40:648–64. doi: 10.1016/j.it.2019.05.003 31155315

[28] Hong J Qu P Wuest TR Huang H Huang C Lin PC. Neutrophilic granule protein is a novel murine lps antagonist. Immune network. (2019) 19:e34. doi: 10.4110/in.2019.19.e34 31720045 PMC6829075

[29] Rudalska R Dauch D Longerich T McJunkin K Wuestefeld T Kang TW. *In vivo* rnai screening identifies a mechanism of sorafenib resistance in liver cancer. Nat Med. (2014) 20:1138–46. doi: 10.1038/nm.3679 PMC4587571 25216638

[30] Seehawer M Heinzmann F D’Artista L Harbig J Roux PF Hoenicke L. Necroptosis microenvironment directs lineage commitment in liver cancer. Nature. (2018) 562:69–75. doi: 10.1038/s41586-018-0519-y 30209397 PMC8111790

[31] Schumann J Stanko K Schliesser U Appelt C Sawitzki B. Differences in cd44 surface expression levels and function discriminates il-17 and ifn-gamma producing helper T cells. PloS One. (2015) 10:e0132479. doi: 10.1371/journal.pone.0132479 26172046 PMC4501817

[32] Sun H Xu J Huang M Huang Q Sun R Xiao W. Cd200r, a co-inhibitory receptor on immune cells, predicts the prognosis of human hepatocellular carcinoma. Immunol Lett. (2016) 178:105–13. doi: 10.1016/j.imlet.2016.08.009 27562325

[33] Bisgin A Meng WJ Adell G Sun XF. Interaction of cd200 overexpression on tumor cells with cd200r1 overexpression on stromal cells: an escape from the host immune response in rectal cancer patients. J Oncol. (2019) 2019:5689464. doi: 10.1155/2019/5689464 30800162 PMC6360612

[34] Huang S Pan Y Zhang Q Sun W. Role of cd200/cd200r signaling pathway in regulation of cd4+T cell subsets during thermal ablation of hepatocellular carcinoma. Med Sci Monit. (2019) 25:1718–28. doi: 10.12659/MSM.913094 PMC6415591 30838977

[35] Yoshimura K Suzuki Y Inoue Y Tsuchiya K Karayama M Iwashita Y. Cd200 and cd200r1 are differentially expressed and have differential prognostic roles in non-small cell lung cancer. Oncoimmunology. (2020) 9:1746554. doi: 10.1080/2162402X.2020.1746554 32395395 PMC7204521

[36] Bhoola NH Mbita Z Hull R Dlamini Z. Translocator protein (Tspo) as a potential biomarker in human cancers. Int J Mol Sci. (2018) 19:2176. doi: 10.3390/ijms19082176 30044440 PMC6121633

[37] Jia JB Ling X Xing M Ludwig JM Bai M Kim HS. Novel tspo-targeted doxorubicin prodrug for colorectal carcinoma cells. Anticancer Res. (2020) 40:5371–8. doi: 10.21873/anticanres.14545 32988856

[38] Wu X Gallo KA. The 18-kda translocator protein (Tspo) disrupts mammary epithelial morphogenesis and promotes breast cancer cell migration. PloS One. (2013) 8:e71258. doi: 10.1371/journal.pone.0071258 23967175 PMC3743866

[39] Phan NN Wang CY Chen CF Sun Z Lai MD Lin YC. Voltage-gated calcium channels: novel targets for cancer therapy. Oncol Lett. (2017) 14:2059–74. doi: 10.3892/ol.2017.6457 PMC5530219 28781648

[40] Wang CY Lai MD Phan NN Sun Z Lin YC. Meta-analysis of public microarray datasets reveals voltage-gated calcium gene signatures in clinical cancer patients. PloS One. (2015) 10:e0125766. doi: 10.1371/journal.pone.0125766 26147197 PMC4493072

[41] Rorvig S Ostergaard O Heegaard NH Borregaard N. Proteome profiling of human neutrophil granule subsets, secretory vesicles, and cell membrane: correlation with transcriptome profiling of neutrophil precursors. J Leukoc Biol. (2013) 94:711–21. doi: 10.1189/jlb.1212619 23650620

[42] Lin D Wu J. Hypoxia inducible factor in hepatocellular carcinoma: A therapeutic target. World J Gastroenterol. (2015) 21:12171–8. doi: 10.3748/wjg.v21.i42.12171 PMC4641134 26576101

[43] Zeng S Lei S Qu C Wang Y Teng S Huang P. Crispr/cas-based gene editing in therapeutic strategies for beta-thalassemia. Hum Genet. (2023) 142:1677–703. doi: 10.1007/s00439-023-02610-9 37878144

[44] Saha D Patgaonkar M Shroff A Ayyar K Bashir T Reddy KV. Hemoglobin expression in nonerythroid cells: novel or ubiquitous? Int J Inflammation. (2014) 2014:803237. doi: 10.1155/2014/803237 PMC4241286 25431740

[45] Lin C-A Chang L-L Zhu H He Q-J Yang B. Hypoxic microenvironment and hepatocellular carcinoma treatment. Hepatoma Res. (2018) 4:26. doi: 10.20517/2394-5079.2018.27

[46] Stankiewicz AM Goscik J Swiergiel AH Majewska A Wieczorek M Juszczak GR. Social stress increases expression of hemoglobin genes in mouse prefrontal cortex. BMC Neurosci. (2014) 15:130. doi: 10.1186/s12868-014-0130-6 25472829 PMC4269175

[47] Kambe J Miyata S Li C Yamamoto Y Nagaoka K. Xanthine-induced deficits in hippocampal behavior and abnormal expression of hemoglobin genes. Behav Brain Res. (2023) 449:114476. doi: 10.1016/j.bbr.2023.114476 37148916

[48] Brown HM Anastasi MR Frank LA Kind KL Richani D Robker RL. Hemoglobin: A gas transport molecule that is hormonally regulated in the ovarian follicle in mice and humans. Biol Reprod. (2015) 92:26. doi: 10.1095/biolreprod.114.124594 25395682

[49] Patrinos GP Kollia P Papadakis MN. Molecular diagnosis of inherited disorders: lessons from hemoglobinopathies. Hum Mutat. (2005) 26:399–412. doi: 10.1002/humu.20225 16138310

[50] Chen Y Ouyang Y Li Z Wang X Ma J. S100a8 and S100a9 in cancer. Biochim Biophys Acta Rev Cancer. (2023) 1878:188891. doi: 10.1016/j.bbcan.2023.188891 37001615

[51] Wang S Song R Wang Z Jing Z Wang S Ma J. S100a8/A9 in inflammation. Front Immunol. (2018) 9:1298. doi: 10.3389/fimmu.2018.01298 29942307 PMC6004386

[52] Gebhardt C Nemeth J Angel P Hess J. S100a8 and S100a9 in inflammation and cancer. Biochem Pharmacol. (2006) 72:1622–31. doi: 10.1016/j.bcp.2006.05.017 16846592

[53] Xia C Braunstein Z Toomey AC Zhong J Rao X. S100 proteins as an important regulator of macrophage inflammation. Front Immunol. (2017) 8:1908. doi: 10.3389/fimmu.2017.01908 29379499 PMC5770888

[54] Ryckman C Vandal K Rouleau P Talbot M Tessier PA. Proinflammatory activities of S100: proteins S100a8, S100a9, and S100a8/A9 induce neutrophil chemotaxis and adhesion. J Immunol. (2003) 170:3233–42. doi: 10.4049/jimmunol.170.6.3233 12626582

[55] Shabani F Farasat A Mahdavi M Gheibi N. Calprotectin (S100a8/S100a9): A key protein between inflammation and cancer. Inflammation research: Off J Eur Histamine Res Soc [et al]. (2018) 67:801–12. doi: 10.1007/s00011-018-1173-4 30083975

[56] Jukic A Bakiri L Wagner EF Tilg H Adolph TE. Calprotectin: from biomarker to biological function. Gut. (2021) 70:1978–88. doi: 10.1136/gutjnl-2021-324855 PMC8458070 34145045

[57] Xia P Ji X Yan L Lian S Chen Z Luo Y. Roles of S100a8, S100a9 and S100a12 in infection, inflammation and immunity. Immunology. (2023) 171:365–76. doi: 10.1111/imm.13722 38013255

[58] Turovskaya O Foell D Sinha P Vogl T Newlin R Nayak J. Rage, carboxylated glycans and S100a8/A9 play essential roles in colitis-associated carcinogenesis. Carcinogenesis. (2008) 29:2035–43. doi: 10.1093/carcin/bgn188 PMC2556970 18689872

[59] Zhang C Yao R Chen J Zou Q Zeng L. S100 family members: potential therapeutic target in patients with hepatocellular carcinoma: A strobe study. Medicine. (2021) 100:e24135. doi: 10.1097/MD.0000000000024135 33546025 PMC7837992

[60] Liu K Zhang Y Zhang C Zhang Q Li J Xiao F. Methylation of S100a8 is a promising diagnosis and prognostic marker in hepatocellular carcinoma. Oncotarget. (2016) 7:56798–810. doi: 10.18632/oncotarget.10792 PMC5302953 27462864

[61] De Ponti A Wiechert L Schneller D Pusterla T Longerich T Hogg N. A pro-tumorigenic function of S100a8/A9 in carcinogen-induced hepatocellular carcinoma. Cancer Lett. (2015) 369:396–404. doi: 10.1016/j.canlet.2015.09.005 26404752

[62] Guedan S Madar A Casado-Medrano V Shaw C Wing A Liu F. Single residue in cd28-costimulated car-T cells limits long-term persistence and antitumor durability. J Clin Invest. (2020) 130:3087–97. doi: 10.1172/JCI133215 PMC7260017 32069268

[63] Rochigneux P Chanez B De Rauglaudre B Mitry E Chabannon C Gilabert M. Adoptive cell therapy in hepatocellular carcinoma: biological rationale and first results in early phase clinical trials. Cancers. (2021) 13:271. doi: 10.3390/cancers13020271 33450845 PMC7828372

[64] Twomey JD Zhang B. Cancer immunotherapy update: fda-approved checkpoint inhibitors and companion diagnostics. AAPS J. (2021) 23:39. doi: 10.1208/s12248-021-00574-0 33677681 PMC7937597

[65] Wang F Jing X Li G Wang T Yang B Zhu Z. Foxp3+ Regulatory T cells are associated with the natural history of chronic hepatitis B and poor prognosis of hepatocellular carcinoma. Liver Int. (2012) 32:644–55. doi: 10.1111/j.1478-3231.2011.02675.x 22118340

[66] Hochnadel I Hoenicke L Petriv N Neubert L Reinhard E Hirsch T. Safety and efficacy of prophylactic and therapeutic vaccine based on live-attenuated listeria monocytogenes in hepatobiliary cancers. Oncogene. (2022) 41:2039–53. doi: 10.1038/s41388-022-02222-z PMC8853207 35173308

[67] Meunier L Larrey D. Drug-induced liver injury: biomarkers, requirements, candidates, and validation. Front Pharmacol. (2019) 10:1482. doi: 10.3389/fphar.2019.01482 31920666 PMC6917655

[68] Schroder SK Gasterich N Weiskirchen S Weiskirchen R. Lipocalin 2 receptors: facts, fictions, and myths. Front Immunol. (2023) 14:1229885. doi: 10.3389/fimmu.2023.1229885 37638032 PMC10451079

[69] Grieshaber-Bouyer R Radtke FA Cunin P Stifano G Levescot A Vijaykumar B. The neutrotime transcriptional signature defines a single continuum of neutrophils across biological compartments. Nat Commun. (2021) 12:2856. doi: 10.1038/s41467-021-22973-9 34001893 PMC8129206

[70] Kim R Emi M Tanabe K. Cancer immunoediting from immune surveillance to immune escape. Immunology. (2007) 121:1–14. doi: 10.1111/j.1365-2567.2007.02587.x 17386080 PMC2265921

[71] Tada Y Togashi Y Kotani D Kuwata T Sato E Kawazoe A. Targeting vegfr2 with ramucirumab strongly impacts effector/activated regulatory T cells and cd8(+) T cells in the tumor microenvironment. J Immunother Cancer. (2018) 6:106. doi: 10.1186/s40425-018-0403-1 30314524 PMC6186121

[72] Zhang L Zhang Z. Recharacterizing tumor-infiltrating lymphocytes by single-cell rna sequencing. Cancer Immunol Res. (2019) 7:1040–6. doi: 10.1158/2326-6066.CIR-18-0658 31262773

[73] Brummel K Eerkens AL de Bruyn M Nijman HW. Tumour-infiltrating lymphocytes: from prognosis to treatment selection. Br J Cancer. (2023) 128:451–8. doi: 10.1038/s41416-022-02119-4 PMC9938191 36564565

