# Supplementary material for: In vivo RNAi screen and validation reveals Ngp, Hba-a1, and S100a8 as novel inhibitory targets on T lymphocytes in liver cancer
Source: Front Immunol. 2025 Apr 25;16:1549229. doi: 10.3389/fimmu.2025.1549229 (PMC12061932; doi:10.3389/fimmu.2025.1549229)
Supplement: Supplementary file 1 [file DataSheet1.pdf]

Supplementary Materials

In vivo RNAi screen and validation reveals Ngp, Hba-a1, and S100a8 as novel inhibitory targets on T lymphocytes in liver cancer

Inga Hochnadel[1§], Lisa Hoenicke[1§], Nataliia Petriv[1§], Huizhen Suo[1], Lothar Groebe[2], Chantal Olijnik[1], Nina Bondarenko[1,3,#], Juan C. Alfonso[4], Michael Jarek[5], Ruibing Shi[6], Andreas Jeron[7,8], Kai Timrott[9], Tatjana Hirsch[7], Nils Jedicke[1], Dunja Bruder[7,8], Frank Klawonn[6,10,11], Ralf Lichtinghagen[12], Robert Geffers[5], Henrike Lenzen[1,13], Michael P. Manns[1], Tetyana Yevsa[1]*

Table of content

Supplementary Abbreviations……………………………………………………………………..2
Supplementary Materials and Methods………………………..……..…………..…………..3
Supplementary Tables…………………………………………………………….…………..…9
Supplementary Figures……………………………………………..………………...……37
Supplementary References………………………………………………………………………56

Supplementary Abbreviations

AFP	Alpha-1-fetoprotein
ALP	Alkaline phosphatase
ALT	Alanine aminotransferase
AST	Aspartate aminotransferase
BMI	Body mass index
CTLA-4	Cytotoxic T-lymphocyte-associated protein
Ctsg	Cathepsin G
GFP	Green fluorescent protein
Hba-a1	Hemoglobin subunit alpha-1
HCC	Hepatocellular carcinoma
HDI	Hydrodynamic tail vein injection
H&E	Hematoxylin and eosin
IHC	Immunohistochemistry
irrelLN	Irrelevant lymph node
LAG3	Lymphocyte-activation gene 3
LCN2	Lypocaline 2
LDH	Lactate dehydrogenase
LN	Lymph node
MPO	Myeloperoxidase
Ngp	Neutrophil granule protein
PBMCs	Peripheral blood mononuclear cells
PD-1	Programmed cell death protein 1
PD-L1	Programmed cell death ligand 1
relLN	Relevant lymph node
Ren	Renilla
RFP	Red fluorescent protein
RNAi	RNA interference
qPCR	Quantitative polymerase chain reaction
SB13	Sleeping Beauty 13 transposase
SEM	Standard error of the mean
shRNA	Short hairpin RNA
TIM3	T cell immunoglobulin and mucin-domain containing 3
Tregs	Regulatory T cells

Supplementary Materials and Methods

Work with human material

The prospective and retrospective studies using human material (human blood and liver tissues obtained after surgeries from patients with hepatocellular carcinoma (HCC)) were conducted in accordance with the Helsinki Declaration and were approved by the Ethics Committee of the MHH (ethical codes: 8742_BO_K_2019). All HCC patients are listed in Tables S1 and S2. Eligible patients were ≥25 years of age (median range: HCC group – 66 years; healthy controls group – 42 years). Appropriate informed consent was received from all the patients. Human peripheral blood mononuclear cells (PBMCs) were isolated as previously described (1). Human liver tissue was digested as previously described (2-5). Obtained single cell suspension from PBMCs and liver tissue was stained with specific antibodies as described in the section “Leukocytes isolation, staining, and sorting**”** and subjected to one-step cell sorting on an Aria-Fusion (BD, Franklin Lakes, USA) sorter**.** RNA isolation and quantitative polymerase chain reaction (qPCR) were performed as described in the section “RNA isolation and qPCR”. All human antibodies and primers for qPCR are listed in Tables S5 and S7.

Immunohistochemistry (IHC)

IHC staining of selected cases (Table S2) was performed on 4 µm-thick formalin-fixed paraffin-embedded tissue sections after heat-induced antigen retrieval in a decloaking chamber (Water Bath 1008, GFL) using citrate buffer (10 mM sodium citrate and 0.05% Tween 20, pH 6.0). Incubation with primary antibodies anti-S100A9 (1:800, antibodies-online.com, Germany) was carried out in 5% bovine serum albumin (BSA, Carl Roth, USA) at room temperature for 1 h. Signal amplification and color development via diaminobenzidine were performed using Epredia™ UltraVision™ Quanto Detection System HRP DAB (horseradish peroxidase (HRP) activity based on the action of 3,3'-diaminobenzidine (DAB)) (Fisher Scientific, USA) and a counter-staining with Mayer’s Hematoxylin (Carl Roth, USA). Images were analysed using a Nikon Eclipse Ti2 inverted microscope (Nikon, Japan). Immune cells positive for the S100A9 were quantified by the experienced pathologist using at least five representative areas (100x) and shown as a cellular density (number of positive events per 1 mm^2).

Animal experiments

All mice used in this study, including age and gender of animals, are listed in Table S3. All animal experiments and procedures were performed in compliance with ethical regulations and the approval of the Lower Saxonian State Office for Consumer Protection and Food Safety (LAVES, Niedersächsisches Landesamt für Verbraucherschutz und Lebensmittelsicherheit; AZ 18/2808, 15/1766, 13/1342). C57BL/6J mice were bred in the facility of the MHH and HZI

(Germany). C57BL/6-Foxp3^{tm1Flv}/J mice were obtained from Jackson Laboratory. All *in vivo* experiments were initiated in mice at 5-8 weeks of age. All animals were maintained under specific pathogen-free conditions in accordance with the institutional guidelines of the MHH/HZI. Chow (standard diet) and water were provided to the animals *ad libitum*.

Vector design

Sleeping Beauty 13 (*SB13*) transposase, *NRAS^{G12V}*, *NRAS^{G12V}-IRES-Ova* (CaNIO), and *Myc-IRES-GFP* (CaMIG; GFP - green fluorescent protein) encoding transposon vectors have been described previously (2-4, 6, 7). CaMIN was generated in this study. To generate CaMIN, *NRAS^{G12V}* was amplified from the CaNIG vector and Bsp and SalI restriction sites were attached using primers NRAS Bsp fw (5'-gtcgccatcatgactgagtacaaac-3') and NRAS SalI rv (5'-tcgccaGtcgacttacatcaccacacatgg-3'). The product was inserted into the vector MLPbcshRen (Ren – Renilla) using NcoI and SalI restriction sites and thereafter amplified using IRES NotI fw (5'-catcaagcggccgcttaggtcagtttatgcaccagagtttc-3') and NRAS AgeI rv (5'-tcgccaaccggtttacatcaccacacatgg-3') primers. *c-Myc* was amplified from CaMIG vector (2-4, 6, 7) and MluI and NotI restriction sites were attached using Myc MluI fw (5'-ggaggaacgcgtagctgtttgaaggctggatttcctttgusing-3') and Myc NotI rv (5'-catcaagcggccgcttaggtcagtttatgcaccagagtttc-3') primers and thereafter inserted into an empty pCaggs transposon vector without gene of interest with a multiple cloning site (pCaggs-MCS) to generate CaM (8). *NRAS^{G12V}-IRES* was finally inserted via AgeI and NotI restriction sites into CaM to generate CaMIN. All vectors used in the study are listed in Table S4.

Induction of autochthonous HCC

To induce HCC (genotype *NRAS^{G12V}/c-Myc*) development, we stably delivered transposable elements encoding *NRAS^{G12V}* and *c-Myc* together with *SB13* into hepatocytes of C57BL/6-Foxp3^{tm1Flv}/J or C57BL/6J mice. Vectors (transposon and *SB13* transposase) for hydrodynamic tail vein injection (HDI) were prepared using QIAGEN EndoFree Maxi Kit (QIAGEN, Hilden, Germany). Transposon and transposase vectors were mixed in a 5:1 molar ratio, and HDI was performed to enable a transposon-mediated stable intrahepatic gene transfer and development of autochthonous liver cancers, as described previously (2-4, 6, 9). All reagents used in the study are listed in Table S4.

Leukocyte isolation, staining, and sorting

Leukocytes were isolated from blood, liver, liver-draining mediastinal and portal lymph nodes (LNs), spleen, and lung as previously described (2-4). Blood was collected from the retro-orbital plexus and mixed with 50 Units heparin (Ratiopharm GmbH, Ulm, Germany). All organs were processed according to established protocols (2-4). The obtained cells were stained with

different antibodies (all used antibodies are listed in Table S5). Data analyses were performed using FlowJo software (Becton Dickinson).

For cell sorting, cells were isolated from spleen, liver-draining LNs, not liver-draining LNs, and livers of C57BL/6-Foxp3^{tm1Flv}/J transgenic mice (10), in which Foxp3^{+} regulatory T cells (Tregs) can be tracked and thereby excluded using red fluorescent protein (RFP^{+}). The obtained cell suspensions were stained with anti-CD3 (clone 145-2C11, Biolegend, California, San Diego, USA), anti-CD4 (clone RM4–5, Biolegend, California, San Diego, USA), anti-CD8 (clone 53-6.7, Biolegend, California, San Diego, USA), anti-CD44 (clone IM7, Biolegend, California, San Diego, USA) and anti-NK1.1 (clone PK136, Biolegend, California, San Diego, USA) antibodies and subjected to one-step cell sorting on a MOFlo (DakoCytomation, Glostrup, Denmark) or a FACSAria (BD Biosciences, Franklin Lakes, New Jersey, USA) sorter. The sorted CD3^{+} NK1.1^{-} CD4^{+} CD8^{-} Foxp3^{-} CD44^{+} and CD3^{+} NK1.1^{-} CD4^{-} CD8^{+} CD44^{+} T lymphocytes were found to be of 98% purity. All antibodies used in the study are listed in Table S5.

Transcriptome analyses (Microarray)

T lymphocytes were isolated using sorting from livers, liver-draining LNs, and spleens of HCC-bearing C57BL/6-Foxp3^{tm1Flv}/J mice with stable intrahepatic overexpression of *NRAS^{G12V}* and *c-Myc* oncogenes, delivered via HDI. T lymphocytes from HCC-free mice, expressing only one oncogene (*c-Myc* or *NRAS^{G12V}*), served as controls, C1 and C2, respectively.

Total RNA from sorted CD3^{+} NK1.1^{-} CD4^{+} CD8^{-} Foxp3^{-} CD44^{+} Foxp3^{-} and CD3^{+} NK1.1^{-} CD8^{+} CD44^{+} T lymphocytes was isolated using the RNeasy kit (Qiagen, Hilden, Germany). The quality and integrity of the total RNA were controlled using an Agilent Technologies 2100 Bioanalyzer (Agilent Technologies, Waldbronn, Germany). Thereafter, 500 ng of total RNA was used for a Cy3-labelling reaction with the one-color Quick Amp Labeling Kit (Agilent Technologies, Waldbronn, Germany). The labeled copy RNA was hybridized to Agilent's mouse 4 x 44k microarrays for 16 h at 68°C. The latters were scanned using the Agilent DNA Microarray Scanner. Two replicates in total were analyzed. Expression values were calculated with the Feature Extraction v10.7.3.1 software package (Agilent Technologies, Waldbronn, Germany).

The obtained data were further analyzed using the R package "limma" (11). Raw data were log2 transformed and quantile normalized. The mean log2 value of both replicates was calculated and used for further analysis. The data presented in the study are deposited in the Gene Expression Omnibus repository, with the accession number GSE144811. All reagents, antibodies, and software used in the study are listed in Tables S4, 5, and 6.

Determination of enzyme activities in plasma

Blood was collected from the retro-orbital plexus and mixed with 50 units of heparin (Ratiopharm GmbH, Ulm, Germany). Biochemical parameters in the obtained plasma were defined as previously described (4).

RNA isolation and qPCR

RNA isolation was performed according to the RNeasy Plus Mini Kit (Qiagen, Hilden, Germany). The qPCR was performed using the SensiFAST™ SYBR® No-ROX One-Step Kit (Bioline, London, UK). 4 µl of isolated RNA were used in duplicates for every sample. Primer sequences were designed using the NCBI platform. All primers for qPCR are listed in Table S7.

Transfection of packaging cells (lentivirus production)

Transfection of HEK293T cells with third generation lentiviral vector system using Pgipz (6), pMD2.G (Addgene, Watertown, Massachusetts, USA), pMDLg/RRE (Addgene, Watertown, Massachusetts, USA) and pRSV-Rev (Addgene, Watertown, Massachusetts, USA) was performed according established protocols as described (12, 13). All DNA and reagents are listed in Table S4.

Transduction

HCC-bearing mice were sacrificed, and liver, liver-draining LNs, not liver-draining LNs, and spleen were isolated, and single-cell suspensions thereof were prepared and stained with anti-CD3, anti-CD4, and anti-CD8 specific antibodies as described in the section "Leukocytes isolation, staining and sorting" using established protocols (2-4). The stained cell suspension was subjected to cell sorting using Aria-II SORP, Aria-IIu, and Aria-Fusion (BD, Franklin Lakes, USA) sorters. Transduction of stimulated T cells was performed using established protocols as described elsewhere (14, 15). All reagents are listed in Table S4.

Short hairpin RNA (shRNA) cloning

shRNAs were ordered as 97-mer DNA oligos from Eurofins (Ebersberg, Germany) and cloned into pGIPZ vector as described previously (6, 16, 17). Cloned sequences were approved using a Mix2Seq kit (Eurofins, Ebersberg, Germany). All reagents and 97-mer DNA sequences are listed in Tables S4 and S8, respectively.

RNA interference (RNAi) screen

HCC development was induced in 5-6 week-old C57BL/6J mice using HDI, as described above (Induction of autochthonous HCC). Upon tumor development, mice were sacrificed and cell

suspensions were isolated from lymph nodes, spleen, and liver. Thereafter, CD4 and CD8 T cells were sorted using MOFlo (DakoCytomation, Glostrup, Denmark) or a FACSAria (BD Biosciences, Franklin Lakes, New Jersey, USA) sorter. Isolated T cells were stimulated with 1 µg/ml anti-CD3, 1 µg/ml anti-CD28, 6.25 ng/ml IL-2, and 0.1 % β-Mercaptoethanol for 72 h at 37°C. Stimulated CD4 and CD8 T cells were transduced with a lentivirus co-expressing the shRNA library and GFP, as described above (Transduction). 48 h post-transduction the successfully transduced GFP^+ CD4 and CD8 T cells were isolated using sorting and labeled with 10 µM of proliferation dye eFluor450 (Thermo Fisher, Waltham, Massachusetts, USA) and adoptively transferred (*i.v.*) in 200 µl of sterile PBS in C57BL/6J HCC-bearing recipient mice. 2×10^5 of CD4 and CD8 were adoptively transferred (in-probes) to each of recipient mice. In total, four recipient mice were used. Five days post-transfer, the recipient mice were sacrificed and the liver, spleen, liver-draining lymph nodes, other lymph nodes, and blood were isolated. Cell suspensions were prepared and stained with anti-CD3, anti-CD4, and anti-CD8 specific antibodies, as described above (Leukocytes isolation, staining and sorting). Adoptively transferred CD4 and CD8 T cells were tracked using the advantage of GFP expression after the transduction with the lentivirus co-expressing shRNA and GFP. The proliferation of cells was tracked using the advantage of proliferation dye eFluor450. T cells were sorted for $CD3^+$ $CD4^+$ GFP^+ $eFluor450^{+/-}$ and $CD3^+$ $CD8^+$ GFP^+ $eFluor450^{+/-}$ populations (out-probes). Thereafter, DNA was isolated from all sorted populations using the DNeasy Blood & Tissue Kit (Qiagen, Hilden, Germany). The obtained DNA was stored at -20°C until the preparation for Illumina sequencing. In total, four recipient mice were adoptively transferred with CD4 and CD8 T cells transduced with library pools.

Illumina sequencing

Illumina sequencing was performed as previously described (18). Briefly, the obtained DNA of previously sorted $CD3^+$ $CD4^+$ GFP^+ $eFluor450^{+/-}$ and $CD3^+$ $CD8^+$ GFP^+ $eFluor450^{+/-}$ T cells was adjusted to 19 ng/µl and 3 µl, corresponding to 57 ng DNA, were used for the 1st PCR reaction mix (20 amplification cycles) (18). The entire PCR product was precipitated with ethanol, as described (18). 1.5 µg of the precipitated DNA was used for the 2nd PCR reaction using NEB index primer (18). The entire product was loaded on a 3% agarose gel and purified using the QIAEX II Gel Extraction Kit (Qiagen, Hilden, Germany). Quality and integrity of total DNA were controlled using a 2100 Bioanalyzer System (Agilent Technologies, Waldbronn, Germany). The sequencing was performed with the NovaSeq6000 system and the NovaSeq 6000 S2 Reagent Kit (100 cycles, paired-end run) with an average of 2×10^7 reads per DNA sample (Illumina, San Diego, CA, USA). The matrix of counts was input to the R package EdgeR (v3.16) (19) in R (v4.2.0) for differential representation analysis. Statistical testing for changes in shRNAs abundance between in-probes groups and out-probes groups was carried out using

the exact test that allows shRNAs to be ranked by significance using the topTags function. All statistical tests were considered significant when $P < 0.05$. The Benjamini–Hochberg method was applied to correct P values for the impact of multiple testing, with false discovery rate-adjusted P values used for statistical analyses. Heatmap plots of the top-ranked shRNAs were generated using log2-fold-change values in all samples.

Histopathological analysis of murine organs

Several murine organs (liver, brain, heart, lung, kidney, pancreas, spleen) were collected for histopathological analysis. Tissue samples were fixed in 4% paraformaldehyde at room temperature for 24-48 hours, embedded in paraffin, and serially sectioned at 4 µm using a rotary microtome (Microm, Germany). Afterwards, tissue sections were stained with hematoxylin and eosin (H&E) (Sigma Aldrich, USA and Merck, Germany), and evaluated for the presence of histopathological changes by the experienced pathologist in a blinded fashion.

Statistics

To calculate the significance of survival analysis, the Mantel-Cox test was used. If not stated otherwise, the unpaired Student's *t*-test was used for all other statistical analyses to calculate significant differences among experimental and control groups in murine studies. The normality of the calculated variables was assessed by using the Kolmogorov-Smirnov test. To compare means, the nonparametric Mann–Whitney *U*-test was used for non-normally distributed data obtained from patients' samples. Statistical analysis of microarray data using the Bayes method is described in the section "Transcriptome analyses (microarray)". If not stated otherwise, data are shown as mean +/- standard error of the mean (SEM) with $P < 0.05$ considered statistically significant. Significance levels were denoted as: *$P < 0.05$, **$P < 0.01$, ***$P < 0.001$ and ****$P < 0.0001$.

Supplementary Tables

Table S1. Clinicopathologic characteristics of the selected patients were analysed using qPCR

	BMI	AFP	GGT (U/L)	Tumor Stage, TNM	Tumor Grade
Patient 1 (HCC)	21	2465	41	pT2	G2
Patient 2 (HCC)	25	2907	17	pT1b	G2
Patient 3 (HCC)	29	N/A	32	N/A	G2
Patient 4 (HCC)	36	3	67	pT1b	G2
Patient 5 (HCC)	31	346	251	pT4	G2
Patient 6 (Healthy liver tissue)	20	N/A	15	-	-
Patient 7 (Healthy liver tissue)	23	N/A	20	-	-
Patient (Healthy controls, donors of PBMCs, n=10)	N/A	N/A	N/A	-	-

BMI – body mass index; AFP – alpha-1-fetoprotein; GGT – gamma-glutamyltransferase; TNM – tumor, nodes, metastasis; N/A – not available.

Table S2. Clinicopathologic characteristics of the selected patients with HCC (n=4) and healthy liver (n=1) were analysed using IHC

	BMI	AFP	GGT (U/L)	Tumor Stage, TNM	Tumor Grade	Microvascular Invasion
Patient 1 (Healthy donor)	23	N/A	20	-	-	-
Patient 2 (HCC)	25	2907	17	pT1b	G2	-
Patient 3 (HCC)	31	346	251	pT4	G2	-
Patient 4 (HCC)	25	4.5	100	pT1b	G3	+
Patient 5 (HCC)	N/A	4.6	150	N/A	G2	-

BMI – body mass index; AFP – alpha-1-fetoprotein; GGT – gamma-glutamyltransferase; TNM – tumor, nodes, metastasis; N/A – not available.

Table S3. Murine strains used in the study

Name	Strain	Supplier	Gender	Age
Mouse	C57BL/6J	Charles River	Males and females	4-8 weeks at HDI
Mouse	C57BL/6-Foxp3^{tm1Flv}/J	Jackson Laboratory	Males and females	6-8 weeks at HDI

Table S4. Reagents

Reagent or Resource	Supplier / Citation	Identifier
SB13 vector		
CaMIG vector	(6, 16)	
CaN vector	(2-4, 6)	
pMD2.G	Addgene	Cat # 12259
pGIPZ	(6)	
pRSV-Rev	Addgene	Cat # 12253
pMDLg/pRRE	Addgene	Cat # 12251
QIAGEN EndoFree Maxi Kit	Qiagen	Cat # 12362
Heparin 5000	Ratiopharm	PZN-03029820
Chloroquine	Sigma Aldrich	Cat # C6628
Polybrene	Sigma Aldrich	Cat # H9268
Calcium chloride	Sigma Aldrich	Cat # C1016
Dulbecco's Modified Eagle Medium (DMEM)	Thermo Fisher	Cat # 31966021
Penicillin/Streptomycin	Gibco	Cat # 15140122
RLT buffer	Qiagen	Cat # 79216
β-Mercaptoethanol	Sigma Aldrich	Cat # M6250
Platinum Pfx polymerase	Invitrogen	Cat # 11708-021
XhoI	New England Biolabs	Cat # R0146
EcoRI	New England Biolabs	Cat # R0101
T4 DNA ligase	New England Biolabs	Cat # M0202
QIAprep Spin Miniprep Kit	Qiagen	Cat # 27104
Qiagen RNeasy Plus Mini Kit	Qiagen	Cat # 74134
Qiagen Plasmid Maxi Kit	Qiagen	Cat # 12362
DH5α	Invitrogen	Cat # 18265-017
Ampicillin	Sigma Aldrich	Cat # A9393

Mix2Seq	Eurofins	N/A
SensiFAST™ SYBR® No-ROX One-Step	Bioline	Cat # BIO-72005
Quick Amp Labeling Kit, one-color	Agilent	Cat # 5190-0442
Collagenase I	Roche	Cat # 11088866001
Collagenase IV	Sigma Aldrich	Cat # C5138
DNase	Goldbio.com	Cat # D-300-500
EDTA	Sigma Aldrich	Cat # E9884
Percoll	GE Healthcare	Cat # 17-0891-01
IL-2	Biolegend	Cat # 575408
NEBNext® Multiplex Oligos for Illumina® (Dual Index Primers Set 1)	NEB	Cat # E7600S
Q5 High-Fidelity DNA Polymerase	NEB	Cat # M0491L
eBioscience™ Cell Proliferation Dye eFluor™ 450	eBioscience	Cat # 65-0842-85
Bovine serum albumin fraction V	Carl Roth	Cat # 8076.4
Hematoxylin	Sigma Aldrich	Cat # 517-28-2
Eosin	Merck	1.115935-0100
Sodium citrate dihydrate	Fisher Scientific	Cat # 11945071
Epredia™ UltraVision™ Quanto Detection System HRP DAB	Fisher Scientific	Cat # 12673997

Table S5. Antibodies

Name	Supplier	Cat no.	Clone no.
**Murine Antibodies**			
Anti-CD16/32	Biolegend	101320	93
Anti-CD3	Biolegend	100222	17A2
Anti-PD-1	Biolegend	109105	RMP1-30
Anti-CD4	Biolegend	100430	GK1.5
Anti-CD8	Biolegend	100761	53-6.7
Anti-CD160	Biolegend	143003	7H1
Anti-LAG3	Biolegend	125219	C9B7W
Anti-4-1BBL	Biolegend	107103	TKS-1
Anti-PDL-1	Biolegend	124334	10F.9G2
Anti-CD44	Biolegend	103044	IM7

Anti-NK1.1	Biolegend	108731	PK136
Anti-4-1BB	Thermo Fisher	17-1371-82	17B5
Anti-CD25	Biolegend	154202	7D4/CD25
Anti-IFNɣ	Biolegend	505809	XMG1.2
Anti-CD40	Biolegend	124605	3/23
Anti-MHCII	Biolegend	107635	M5/114.15.2
Anti-CD68	Biolegend	137021	FA-11
Anti-CD80	Biolegend	104731	16-10A1
Anti-CD19	BD Biosciences	563157	1D3
Anti-CD11b	Biolegend	101243	M1/70
Anti-Ly6G	Biolegend	127607	1A8
Anti-Gr1	Biolegend	108403	RB6-8C5
Anti-IL10	Biolegend	505009	JES5-16E3
Anti-Ly6C	Biolegend	108421	RB6-8C5
Anti-CD11c	Biolegend	117323	N418
Anti-CD3 ultra leaf purified	Biolegend	100359	145-2C11
Anti-CD28 ultra leaf purified	Biolegend	102121	37.51
**Human antibodies**			
CD3	Biolegend	300318	HIT3a
CD4	Biolegend	357418	A161A1
CD8	BD	612754	HIT8a
CD19	Biolegend	302246	HIB19
CD56	Biolegend	362510	5.1H11
S100A9	antibodies-online.com	ABIN7012543	Polyclonal

Table S6. Software

Software name	Manufacturer	Version
FlowJo	Treestar Inc.	9.9.6
Graphpad prism	GraphPad Software, Inc.	5
R package limmaGUI	Smyth	
R package EdgeR	Smyth	v3.16

Table S7. DNA sequences

Name	Sequence	Supplier
5‘miR30 XhoI fw	5’-CAGAAGGCTCGAGAAGGTATATTG CTGTTGACAGTGAGCG-3’	Eurofins
3‘miR30 EcoRI rev	5’-CTAAAGTAGCCCCTTGAATTCCGAGG CAGTAGGCA-3’	Eurofins
Ngp	5’-AGACCCCTCTTCCGCCTGCTA-3’	Eurofins
Ngp	3’-CCTGTGCAATTTCTCTCCTCCCCAT-5’	Eurofins
Hbb-b1	5’-ATGGCCTGAATCACTTGGAC-3’	Eurofins
Hbb-b1	3’-ACGATCATATTGCCCAGGAG-5’	Eurofins
Hprt	5’-CAGGACTGAAAGACTTGCTCG-3’	Eurofins
Hprt	3’-GTTGACTGATCATTACAGTAGCTC-5’	Eurofins
LCN2	5’- TCACCTCCGTCCTGTTTAGG -3’	Eurofins
LCN2	3’- AGGTAACTCGTTAATCCAGGGTAA -5’	Eurofins
S100A8	5’-TGGGCATCATGTTGACCGAG-3’	Eurofins
S100A8	3’-CGTCTGCACCCTTTTTCCTGAT-5’	Eurofins
S100A9	5’- GTGCGAAAAGATCTGCAAAA -3’	Eurofins
S100A9	3’- TCAGCTGCTTGTCTGCATTT -5’	Eurofins
CAMP	5’- TCGGATGCTAACCTCTACCG -3’	Eurofins
CAMP	3’- ACAGGCTTTGGCGTGTCT -5’	Eurofins
HBB	5’- GCACGTGGATCCTGAGAACT -3’	Eurofins
HBB	3’- CACTGGTGGGGTGAATTCTT -5’	Eurofins
GAPDH	5’-GAAGGTGAAGGTCGGACTC-3’	Eurofins
GAPDH	3’-GAAGATGGTGATGGGATTTC-5’	Eurofins

Table S8. 97-mer DNA sequences for shRNA cloning procedure

Name	Sequence	Supplier
Pdcd1.1100	TGCTGTTGACAGTGAGCGATGAGAGCTCACTTCAGG TTTATAGTGAAGCCACAGATGTATAAACCTGAAGTGA GCTCTCACTGCCTACTGCCTCGGA	Eurofins
Pdcd1.19	TGCTGTTGACAGTGAGCGAGAAGAGGAGACTGCTAC TGAATAGTGAAGCCACAGATGTATTCAGTAGCAGTCT CCTCTTCCTGCCTACTGCCTCGGA	Eurofins
Pdcd1.370	TGCTGTTGACAGTGAGCGCCAGGCATGACTTCCACA TGAATAGTGAAGCCACAGATGTATTCATGTGGAAGT CATGCCTGTTGCCTACTGCCTCGGA	Eurofins
Pdcd1.630	TGCTGTTGACAGTGAGCGACCCTAGCTGTCTTCTGC TCAATAGTGAAGCCACAGATGTATTGAGCAGAAGAC AGCTAGGGCTGCCTACTGCCTCGGA	Eurofins
Pdcd1.633	TGCTGTTGACAGTGAGCGATAGCTGTCTTCTGCTCA ACAATAGTGAAGCCACAGATGTATTGTTGAGCAGAA GACAGCTAGTGCCTACTGCCTCGGA	Eurofins
Ctla4.1015	TGCTGTTGACAGTGAGCGATGGGATATTTCTGTTGT GTCATAGTGAAGCCACAGATGTATGACACAACAGAA ATATCCCAGTGCCTACTGCCTCGGA	Eurofins

Ctla4.1067	TGCTGTTGACAGTGAGCGACAGCATAAGGATATAGCATTATAGTGAAGCCACAGATGTATAATGCTATATCCTTATGCTGCTGCCTACTGCCTCGGA	Eurofins
Ctla4.1308	TGCTGTTGACAGTGAGCGCAAGGTTGTAGTGTTCCAAGATTAGTGAAGCCACAGATGTAATCTTGGAACACTACAACCTTTTGCCTACTGCCTCGGA	Eurofins
Ctla4.1545	TGCTGTTGACAGTGAGCGCACCGATGGTGATGGAGTGTAATAGTGAAGCCACAGATGTATTACACTCCATCACCATCGGTTTGCCTACTGCCTCGGA	Eurofins
Ctla4.405	TGCTGTTGACAGTGAGCGACACGACATTCACAGAGAAGAATAGTGAAGCCACAGATGTATTCTTCTCTGTGAATGTCGTGGTGCCTACTGCCTCGGA	Eurofins
Lag3.1066	TGCTGTTGACAGTGAGCGCCACCTACAGAGATGGCTTCAATAGTGAAGCCACAGATGTATTGAAGCCATCTCTGTAGGTGATGCCTACTGCCTCGGA	Eurofins
Lag3.1266	TGCTGTTGACAGTGAGCGACTGGAAAGAGTGGCAATTTTATAGTGAAGCCACAGATGTATAAAATTGCCACTCTTTCCAGCTGCCTACTGCCTCGGA	Eurofins
Lag3.1760	TGCTGTTGACAGTGAGCGCCAGTTGCTACTGAGAAGATTTTAGTGAAGCCACAGATGTAAAATCTTCTCAGTAGCAACTGTTGCCTACTGCCTCGGA	Eurofins
Lag3.41	TGCTGTTGACAGTGAGCGAGAGCAGCTCAAGTTCTAGCTATAGTGAAGCCACAGATGTATAGCTAGAACTTGAGCTGCTCCTGCCTACTGCCTCGGA	Eurofins
Lag3.503	TGCTGTTGACAGTGAGCGAAACCTGGATCCTAACTTTCTATAGTGAAGCCACAGATGTATAGAAAGTTAGGATCCAGGTTGTGCCTACTGCCTCGGA	Eurofins
Cd160.1113	TGCTGTTGACAGTGAGCGCAAGCACGTTTTTAAAAGATAATAGTGAAGCCACAGATGTATTATCTTTTAAAAACGTGCTTATGCCTACTGCCTCGGA	Eurofins
Cd160.1322	TGCTGTTGACAGTGAGCGATAGGTAGAGAACAAGAGGAAATAGTGAAGCCACAGATGTATTTCCTCTTGTTCTCTACCTACTGCCTACTGCCTCGGA	Eurofins
Cd160.1716	TGCTGTTGACAGTGAGCGCCAGTGTTTTTCTAAGAATCAATAGTGAAGCCACAGATGTATTGATTCTTAGAAAAACACTGTTGCCTACTGCCTCGGA	Eurofins
Cd160.1836	TGCTGTTGACAGTGAGCGCCCCGTTTGTAACTTTAAGTAATAGTGAAGCCACAGATGTATTACTTAAAGTTACAAACGGGTTGCCTACTGCCTCGGA	Eurofins
Cd160.1837	TGCTGTTGACAGTGAGCGACCGTTTGTAACTTTAAGTAAATAGTGAAGCCACAGATGTATTTACTTAAAGTTACAAACGGGTGCCTACTGCCTCGGA	Eurofins
Havcr2.1390	TGCTGTTGACAGTGAGCGATGGCATATGCTTAACACTATATAGTGAAGCCACAGATGTATATAGTGTTAAGCATATGCCACTGCCTACTGCCTCGGA	Eurofins
Havcr2.1788	TGCTGTTGACAGTGAGCGCCAGAAGAGACATGCACATGAATAGTGAAGCCACAGATGTATTCATGTGCATGTCTCTTCTGATGCCTACTGCCTCGGA	Eurofins
Havcr2.2049	TGCTGTTGACAGTGAGCGACAGACTCTTCTTACAGAAGAATAGTGAAGCCACAGATGTATTCTTCTGTAAGAAGAGTCTGGTGCCTACTGCCTCGGA	Eurofins
Havcr2.2249	TGCTGTTGACAGTGAGCGAAGCAATCTATATAGTCAGCAATAGTGAAGCCACAGATGTATTGCTGACTATATAGATTGCTGTGCCTACTGCCTCGGA	Eurofins

Havcr2.2431	TGCTGTTGACAGTGAGCGACAGAAAGGTAGCTTTGT TCAATAGTGAAGCCACAGATGTATTGAACAAAGCTAC CTTTCTGCTGCCTACTGCCTCGGA	Eurofins
Lgals9.1025	TGCTGTTGACAGTGAGCGACAGGATATCAACACTCT AGAATAGTGAAGCCACAGATGTATTCTAGAGTGTTGA TATCCTGCTGCCTACTGCCTCGGA	Eurofins
Lgals9.1165	TGCTGTTGACAGTGAGCGCCAGAAGAGATCATCACT TATATAGTGAAGCCACAGATGTATATAAGTGATGATC TCTTCTGATGCCTACTGCCTCGGA	Eurofins
Lgals9.439	TGCTGTTGACAGTGAGCGCCAAGGTGATGGTGAACA AGAATAGTGAAGCCACAGATGTATTCTTGTTCACCAT CACCTTGATGCCTACTGCCTCGGA	Eurofins
Lgals9.440	TGCTGTTGACAGTGAGCGAAAGGTGATGGTGAACAA GAAATAGTGAAGCCACAGATGTATTTCTTGTTCACCA TCACCTTGTGCCTACTGCCTCGGA	Eurofins
Lgals9.850	TGCTGTTGACAGTGAGCGCCCGAAACACTCAGATCA ACAATAGTGAAGCCACAGATGTATTGTTGATCTGAGT GTTTCGGATGCCTACTGCCTCGGA	Eurofins
Btla.1024	TGCTGTTGACAGTGAGCGAAACCTGGAAAGTTTCAA TTAATAGTGAAGCCACAGATGTATTAATTGAAACTTT CCAGGTTGTGCCTACTGCCTCGGA	Eurofins
Btla.1650	TGCTGTTGACAGTGAGCGATAGTGTTTGGATCAAGA TTAATAGTGAAGCCACAGATGTATTAATCTTGATCCA AACACTACTGCCTACTGCCTCGGA	Eurofins
Btla.1670	TGCTGTTGACAGTGAGCGCACGCACATGAATGTGAT TCAATAGTGAAGCCACAGATGTATTGAATCACATTCA TGTGCGTTTGCCTACTGCCTCGGA	Eurofins
Btla.2303	TGCTGTTGACAGTGAGCGAAAGGTGCAAAGTACTAG AGAATAGTGAAGCCACAGATGTATTCTCTAGTACTTT GCACCTTCTGCCTACTGCCTCGGA	Eurofins
Btla.2733	TGCTGTTGACAGTGAGCGCAACGTTGTGTGTACATG TATATAGTGAAGCCACAGATGTATATACATGTACACA CAACGTTTTGCCTACTGCCTCGGA	Eurofins
Cd244.1183	TGCTGTTGACAGTGAGCGACAGGAAGTCTGGATCCA AGAATAGTGAAGCCACAGATGTATTCTTGGATCCAG ACTTCCTGGTGCCTACTGCCTCGGA	Eurofins
Cd244.1434	TGCTGTTGACAGTGAGCGATGGAGAGAATGTAGTTT ATTATAGTGAAGCCACAGATGTATAATAAACTACATT CTCTCCAGTGCCTACTGCCTCGGA	Eurofins
Cd244.1562	TGCTGTTGACAGTGAGCGACAGCTTCTAGGCTGAAG AGAATAGTGAAGCCACAGATGTATTCTCTTCAGCCTA GAAGCTGGTGCCTACTGCCTCGGA	Eurofins
Cd244.1882	TGCTGTTGACAGTGAGCGCCCCAAGCTTATAGATCA GATATAGTGAAGCCACAGATGTATATCTGATCTATAA GCTTGGGTTGCCTACTGCCTCGGA	Eurofins
Cd244.269	TGCTGTTGACAGTGAGCGAACAAAAGATGTTTCTGTT CAATAGTGAAGCCACAGATGTATTGAACAGAAACATC TTTTGTCTGCCTACTGCCTCGGA	Eurofins
4632428N05Rik.19 23	TGCTGTTGACAGTGAGCGAACCGACCTCCACAATGT CCTATAGTGAAGCCACAGATGTATAGGACATTGTGG AGGTCGGTCTGCCTACTGCCTCGGA	Eurofins
4632428N05Rik.22 76	TGCTGTTGACAGTGAGCGCAAGGGTAGTTCTTGTGA GGTATAGTGAAGCCACAGATGTATACCTCACAAGAA CTACCCTTTTGCCTACTGCCTCGGA	Eurofins

4632428N05Rik.2753	TGCTGTTGACAGTGAGCGATAGATCTATGCAGTTACTATATAGTGAAGCCACAGATGTATATAGTAACTGCATAGATCTAGTGCCTACTGCCTCGGA	Eurofins
4632428N05Rik.3523	TGCTGTTGACAGTGAGCGACAGGAGGAAAGGTGTCTGATATAGTGAAGCCACAGATGTATATCAGACACCTTTCCTCCTGGTGCCTACTGCCTCGGA	Eurofins
4632428N05Rik.361	TGCTGTTGACAGTGAGCGCAGGGCACGATGTGACCATCTATAGTGAAGCCACAGATGTATAGATGGTCACATCGTGCCCTTTGCCTACTGCCTCGGA	Eurofins
Tigit.170	TGCTGTTGACAGTGAGCGAAGCAGGCACGATAGATACAAATAGTGAAGCCACAGATGTATTTGTATCTATCGTGCCTGCTGTGCCTACTGCCTCGGA	Eurofins
Tigit.172	TGCTGTTGACAGTGAGCGACAGGCACGATAGATACAAAGATAGTGAAGCCACAGATGTATCTTTGTATCTATCGTGCCTGCTGCCTACTGCCTCGGA	Eurofins
Tigit.176	TGCTGTTGACAGTGAGCGACACGATAGATACAAAGAGGAATAGTGAAGCCACAGATGTATTCCTCTTTGTATCTATCGTGCTGCCTACTGCCTCGGA	Eurofins
Tigit.577	TGCTGTTGACAGTGAGCGATACTGGCTAGAAAGAAGTCTATAGTGAAGCCACAGATGTATAGACTTCTTTCTAGCCAGTACTGCCTACTGCCTCGGA	Eurofins
Tigit.580	TGCTGTTGACAGTGAGCGATGGCTAGAAAGAAGTCTATTATAGTGAAGCCACAGATGTATAATAGACTTCTTTCTAGCCAGTGCCTACTGCCTCGGA	Eurofins
Fgl2.1783	TGCTGTTGACAGTGAGCGCAAGCATTTTAGTTCTCAAGAATAGTGAAGCCACAGATGTATTCTTGAGAACTAAAATGCTTTTGCCTACTGCCTCGGA	Eurofins
Gypa.1017	TGCTGTTGACAGTGAGCGACACAAACATAAATGTATGTAATAGTGAAGCCACAGATGTATTACATACATTTATGTTTGTGGTGCCTACTGCCTCGGA	Eurofins
Car2.1234	TGCTGTTGACAGTGAGCGCTCCGTTGTGCTTACTAACAAATAGTGAAGCCACAGATGTATTTGTTAGTAAGCACAACGGATTGCCTACTGCCTCGGA	Eurofins
Ces2g.1082	TGCTGTTGACAGTGAGCGACAGCATCATTGGTTTCAACAATAGTGAAGCCACAGATGTATTGTTGAAACCAATGATGCTGGTGCCTACTGCCTCGGA	Eurofins
Kel.1099	TGCTGTTGACAGTGAGCGCCATGTGGTTACTATTGACGAATAGTGAAGCCACAGATGTATTCGTCAATAGTAACCACATGATGCCTACTGCCTCGGA	Eurofins
Gypa.1017	TGCTGTTGACAGTGAGCGACACAAACATAAATGTATGTAATAGTGAAGCCACAGATGTATTACATACATTTATGTTTGTGGTGCCTACTGCCTCGGA	Eurofins
Fgl2.1783	TGCTGTTGACAGTGAGCGCAAGCATTTTAGTTCTCAAGAATAGTGAAGCCACAGATGTATTCTTGAGAACTAAAATGCTTTTGCCTACTGCCTCGGA	Eurofins
Ces2g.1082	TGCTGTTGACAGTGAGCGACAGCATCATTGGTTTCAACAATAGTGAAGCCACAGATGTATTGTTGAAACCAATGATGCTGGTGCCTACTGCCTCGGA	Eurofins
Car2.1234	TGCTGTTGACAGTGAGCGCTCCGTTGTGCTTACTAACAAATAGTGAAGCCACAGATGTATTTGTTAGTAAGCACAACGGATTGCCTACTGCCTCGGA	Eurofins
Spp1.1058	TGCTGTTGACAGTGAGCGCTAGTCTGTTGTTTCATGCAAATAGTGAAGCCACAGATGTATTTGCATGAAACAACAGACTAATGCCTACTGCCTCGGA	Eurofins

Spp1.213	TGCTGTTGACAGTGAGCGCAAGGATGACTTTAAGCA<br>AGAATAGTGAAGCCACAGATGTATTCTTGCTTAAAGT<br>CATCCTTTTGCCTACTGCCTCGGA	Eurofins
Spp1.273	TGCTGTTGACAGTGAGCGAGACGACGATGATGACGA<br>TGATTAGTGAAGCCACAGATGTAATCATCGTCATCAT<br>CGTCGTCCTGCCTACTGCCTCGGA	Eurofins
Spp1.381	TGCTGTTGACAGTGAGCGAACCGTCACTGCTAGTAC<br>ACAATAGTGAAGCCACAGATGTATTGTGTACTAGCA<br>GTGACGGTCTGCCTACTGCCTCGGA	Eurofins
Spp1.732	TGCTGTTGACAGTGAGCGATCGGATGTGATCGATAG<br>TCAATAGTGAAGCCACAGATGTATTGACTATCGATCA<br>CATCCGACTGCCTACTGCCTCGGA	Eurofins
Samd3.104	TGCTGTTGACAGTGAGCGATAGGAGAAGGTGTATTG<br>ACAATAGTGAAGCCACAGATGTATTGTCAATACACCT<br>TCTCCTACTGCCTACTGCCTCGGA	Eurofins
Samd3.498	TGCTGTTGACAGTGAGCGAAAGCAGAGAAGAAATGT<br>AAAATAGTGAAGCCACAGATGTATTTTACATTTCTTCT<br>CTGCTTCTGCCTACTGCCTCGGA	Eurofins
Samd3.656	TGCTGTTGACAGTGAGCGCCCAGGCAGATATGACTA<br>AGTATAGTGAAGCCACAGATGTATACTTAGTCATATC<br>TGCCTGGATGCCTACTGCCTCGGA	Eurofins
Samd3.825	TGCTGTTGACAGTGAGCGCCCCATAGAAGATGATGA<br>ACAATAGTGAAGCCACAGATGTATTGTTCATCATCTT<br>CTATGGGTTGCCTACTGCCTCGGA	Eurofins
Samd3.829	TGCTGTTGACAGTGAGCGCTAGAAGATGATGAACAA<br>GTAATAGTGAAGCCACAGATGTATTACTTGTTCATCA<br>TCTTCTATTGCCTACTGCCTCGGA	Eurofins
Kel.1099	TGCTGTTGACAGTGAGCGCCATGTGGTTACTATTGA<br>CGAATAGTGAAGCCACAGATGTATTCGTCAATAGTAA<br>CCACATGATGCCTACTGCCTCGGA	Eurofins
Kel.1272	TGCTGTTGACAGTGAGCGCCAGGGAGAGTATACAGA<br>GCTATAGTGAAGCCACAGATGTATAGCTCTGTATACT<br>CTCCCTGTTGCCTACTGCCTCGGA	Eurofins
Kel.1678	TGCTGTTGACAGTGAGCGCCAGGAATATAATGATAT<br>ACAATAGTGAAGCCACAGATGTATTGTATATCATTAT<br>ATTCCTGTTGCCTACTGCCTCGGA	Eurofins
Kel.1814	TGCTGTTGACAGTGAGCGAAGGTCAATGCTTACTATT<br>CAATAGTGAAGCCACAGATGTATTGAATAGTAAGCAT<br>TGACCTCTGCCTACTGCCTCGGA	Eurofins
Kel.2500	TGCTGTTGACAGTGAGCGCAAGCGTGTATTTAGCTT<br>TAAATAGTGAAGCCACAGATGTATTTAAAGCTAAATA<br>CACGCTTTTGCCTACTGCCTCGGA	Eurofins
Cldn13.380	TGCTGTTGACAGTGAGCGATGGATACAGTGTACACT<br>GTATTAGTGAAGCCACAGATGTAATACAGTGTACACT<br>GTATCCACTGCCTACTGCCTCGGA	Eurofins
Cldn13.384	TGCTGTTGACAGTGAGCGCTACAGTGTACACTGTAT<br>GACATAGTGAAGCCACAGATGTATGTCATACAGTGT<br>ACACTGTATTGCCTACTGCCTCGGA	Eurofins
Cldn13.508	TGCTGTTGACAGTGAGCGAGCGCATCAATTGTTTCA<br>TGAATAGTGAAGCCACAGATGTATTCATGAAACAATT<br>GATGCGCCTGCCTACTGCCTCGGA	Eurofins
Cldn13.655	TGCTGTTGACAGTGAGCGCTGGCTTCTCGAAGAAAG<br>TACATAGTGAAGCCACAGATGTATGTACTTTCTTCGA<br>GAAGCCAATGCCTACTGCCTCGGA	Eurofins

Cldn13.815	TGCTGTTGACAGTGAGCGAAACAACGATACCTTAGATGTTTAGTGAAGCCACAGATGTAAACATCTAAGGTATCGTTGTTGTGCCTACTGCCTCGGA	Eurofins
Gypa.1017	TGCTGTTGACAGTGAGCGACACAAACATAAATGTATGTAATAGTGAAGCCACAGATGTATTACATACATTTATGTTTGTGGTGCCTACTGCCTCGGA	Eurofins
Gypa.1057	TGCTGTTGACAGTGAGCGCTCAGTGTGTAAGTCAATACAATAGTGAAGCCACAGATGTATTGTATTGACTTACACACTGAATGCCTACTGCCTCGGA	Eurofins
Gypa.1058	TGCTGTTGACAGTGAGCGCCAGTGTGTAAGTCAATACAAATAGTGAAGCCACAGATGTATTTGTATTGACTTACACACTGATGCCTACTGCCTCGGA	Eurofins
Gypa.123	TGCTGTTGACAGTGAGCGCCAGATTCTCTTCTTCAAATAATAGTGAAGCCACAGATGTATTATTTGAAGAAGAGAATCTGATGCCTACTGCCTCGGA	Eurofins
Gypa.1527	TGCTGTTGACAGTGAGCGCAGGGATGAAAATGTTCAAAATTAGTGAAGCCACAGATGTAATTTTGAACATTTTCATCCCTTTGCCTACTGCCTCGGA	Eurofins
Car2.1234	TGCTGTTGACAGTGAGCGCTCCGTTGTGCTTACTAACAAATAGTGAAGCCACAGATGTATTTGTTAGTAAGCACAACGGATTGCCTACTGCCTCGGA	Eurofins
Car2.1556	TGCTGTTGACAGTGAGCGCCCCTTCCAAGATCTTATATTATAGTGAAGCCACAGATGTATAATATAAGATCTTGGAAGGGTTGCCTACTGCCTCGGA	Eurofins
Car2.1562	TGCTGTTGACAGTGAGCGACAAGATCTTATATTAAAGAAATAGTGAAGCCACAGATGTATTTCTTTAATATAAGATCTTGGTGCCTACTGCCTCGGA	Eurofins
Car2.1608	TGCTGTTGACAGTGAGCGATAGCAAAGTTATTCTTAAATATAGTGAAGCCACAGATGTATATTTAAGAATAACTTTGCTACTGCCTACTGCCTCGGA	Eurofins
Car2.1740	TGCTGTTGACAGTGAGCGCCACAATGGATAATGTATTTTATAGTGAAGCCACAGATGTATAAAATACATTATCCATTGTGTTGCCTACTGCCTCGGA	Eurofins
Ank1.174	TGCTGTTGACAGTGAGCGACACAAAGAGATCATTCTAGAATAGTGAAGCCACAGATGTATTCTAGAATGATCTCTTTGTGCTGCCTACTGCCTCGGA	Eurofins
Ank1.4463	TGCTGTTGACAGTGAGCGACAGGCAGAGCAGAAACCTCAATAGTGAAGCCACAGATGTATTGAGGTTTCTGCTCTGCCTGCTGCCTACTGCCTCGGA	Eurofins
Ank1.5832	TGCTGTTGACAGTGAGCGCCGGGTGCAGACACGAATAATATAGTGAAGCCACAGATGTATATTATTCGTGTCTGCACCCGTTGCCTACTGCCTCGGA	Eurofins
Ank1.6468	TGCTGTTGACAGTGAGCGCTAGTCTGTGTTCAAAGTGTAATAGTGAAGCCACAGATGTATTACACTTTGAACACAGACTATTGCCTACTGCCTCGGA	Eurofins
Ank1.7713	TGCTGTTGACAGTGAGCGCCACCAGCTAAACAAATGTCAATAGTGAAGCCACAGATGTATTGACATTTGTTTAGCTGGTGTTGCCTACTGCCTCGGA	Eurofins
Atp1b2.1349	TGCTGTTGACAGTGAGCGAAACGTGGAGGTGAATGTTGAATAGTGAAGCCACAGATGTATTCAACATTCACCTCCACGTTGTGCCTACTGCCTCGGA	Eurofins
Atp1b2.1804	TGCTGTTGACAGTGAGCGCCAGCTCCTGTTTCAGTTTCTATAGTGAAGCCACAGATGTATAGAAACTGAAACAGGAGCTGTTGCCTACTGCCTCGGA	Eurofins

Atp1b2.2500	TGCTGTTGACAGTGAGCGAAAGTTGCTAACTGGTCA CTAATAGTGAAGCCACAGATGTATTAGTGACCAGTTA GCAACTTGTGCCTACTGCCTCGGA	Eurofins
Atp1b2.2656	TGCTGTTGACAGTGAGCGCTCCCTAGAATGTTGCGA AAGATAGTGAAGCCACAGATGTATCTTTCGCAACATT CTAGGGATTGCCTACTGCCTCGGA	Eurofins
Atp1b2.901	TGCTGTTGACAGTGAGCGATCAGCATGTTCAGAAGC TCAATAGTGAAGCCACAGATGTATTGAGCTTCTGAAC ATGCTGACTGCCTACTGCCTCGGA	Eurofins
Aqp1.1783	TGCTGTTGACAGTGAGCGAAACGAAGGTCATTTTGT GGTATAGTGAAGCCACAGATGTATACCACAAAATGA CCTTCGTTCTGCCTACTGCCTCGGA	Eurofins
Aqp1.1968	TGCTGTTGACAGTGAGCGCGAGAAGCAGCTAGCTAT GCAATAGTGAAGCCACAGATGTATTGCATAGCTAGC TGCTTCTCTTGCCTACTGCCTCGGA	Eurofins
Aqp1.2035	TGCTGTTGACAGTGAGCGACCCTTAGTTCATCATGA GCAATAGTGAAGCCACAGATGTATTGCTCATGATGA ACTAAGGGCTGCCTACTGCCTCGGA	Eurofins
Aqp1.2329	TGCTGTTGACAGTGAGCGCCAGGTGCTTAGAAGCAG CAGATAGTGAAGCCACAGATGTATCTGCTGCTTCTAA GCACCTGATGCCTACTGCCTCGGA	Eurofins
Aqp1.2474	TGCTGTTGACAGTGAGCGCTGGATCAAGCTTAGCAG TCAATAGTGAAGCCACAGATGTATTGACTGCTAAGCT TGATCCAATGCCTACTGCCTCGGA	Eurofins
Tspo2.170	TGCTGTTGACAGTGAGCGAAGAGAGAAGGCTTCAGA GAAATAGTGAAGCCACAGATGTATTTCTCTGAAGCCT TCTCTCTCTGCCTACTGCCTCGGA	Eurofins
Tspo2.262	TGCTGTTGACAGTGAGCGACAGCTTCAAGGACCTGT CTTTTAGTGAAGCCACAGATGTAAAAGACAGGTCCTT GAAGCTGCTGCCTACTGCCTCGGA	Eurofins
Tspo2.342	TGCTGTTGACAGTGAGCGATCGGTGTGAGGATGAGA GAAATAGTGAAGCCACAGATGTATTTCTCTCATCCTC ACACCGACTGCCTACTGCCTCGGA	Eurofins
Tspo2.432	TGCTGTTGACAGTGAGCGCTGCCTCCTACCTGGTAT GGAATAGTGAAGCCACAGATGTATTCCATACCAGGT AGGAGGCATTGCCTACTGCCTCGGA	Eurofins
Tspo2.53	TGCTGTTGACAGTGAGCGACAGGCTGTTGTGTCCTT GAGATAGTGAAGCCACAGATGTATCTCAAGGACACA ACAGCCTGGTGCCTACTGCCTCGGA	Eurofins
Myh10.2158	TGCTGTTGACAGTGAGCGAAAGGACGTGGACCGAAT TGTATAGTGAAGCCACAGATGTATACAATTCGGTCCA CGTCCTTCTGCCTACTGCCTCGGA	Eurofins
Myh10.3613	TGCTGTTGACAGTGAGCGACACAAGAATAATGCACT TAAATAGTGAAGCCACAGATGTATTTAAGTGCATTAT TCTTGTGCTGCCTACTGCCTCGGA	Eurofins
Myh10.495	TGCTGTTGACAGTGAGCGCTGGGAAGAAAGCAATGG TCAATAGTGAAGCCACAGATGTATTGACCATTGCTTT CTTCCCATTGCCTACTGCCTCGGA	Eurofins
Myh10.7338	TGCTGTTGACAGTGAGCGATACAGTACTAAGACAAT GTTATAGTGAAGCCACAGATGTATAACATTGTCTTAG TACTGTAGTGCCTACTGCCTCGGA	Eurofins
Myh10.7340	TGCTGTTGACAGTGAGCGCCAGTACTAAGACAATGT TAAATAGTGAAGCCACAGATGTATTTAACATTGTCTT AGTACTGTTGCCTACTGCCTCGGA	Eurofins

Ptpla.360	TGCTGTTGACAGTGAGCGCCACACAGAGGTTTATATAAAATAGTGAAGCCACAGATGTATTTTATATAAACCTCTGTGTGTTGCCTACTGCCTCGGA	Eurofins
Ptpla.509	TGCTGTTGACAGTGAGCGATGGCTCATTACTCACAGTATATAGTGAAGCCACAGATGTATATACTGTGAGTAATGAGCCACTGCCTACTGCCTCGGA	Eurofins
Ptpla.713	TGCTGTTGACAGTGAGCGCACCGTTGTTTCCTCAGCTCTATAGTGAAGCCACAGATGTATAGAGCTGAGGAAACAACGGTATGCCTACTGCCTCGGA	Eurofins
Ptpla.726	TGCTGTTGACAGTGAGCGCCAGCTCTATTTTCATATGTTATAGTGAAGCCACAGATGTATAACATATGAAAATAGAGCTGATGCCTACTGCCTCGGA	Eurofins
Ptpla.835	TGCTGTTGACAGTGAGCGCAACCGGATTACTTGAGTCCAATAGTGAAGCCACAGATGTATTGGACTCAAGTAATCCGGTTTTGCCTACTGCCTCGGA	Eurofins
1300017J02Rik.1337	TGCTGTTGACAGTGAGCGATGGCAGAGAACTACTTGTCTATAGTGAAGCCACAGATGTATAGACAAGTAGTTCTCTGCCAGTGCCTACTGCCTCGGA	Eurofins
1300017J02Rik.1526	TGCTGTTGACAGTGAGCGCTGGGTTTAATATACAACCAAATAGTGAAGCCACAGATGTATTTGGTTGTATATTAAACCCATTGCCTACTGCCTCGGA	Eurofins
1300017J02Rik.1918	TGCTGTTGACAGTGAGCGCAAGGCTGACTTTGTTCGAAGATAGTGAAGCCACAGATGTATCTTCGAACAAAGTCAGCCTTATGCCTACTGCCTCGGA	Eurofins
1300017J02Rik.1954	TGCTGTTGACAGTGAGCGCCAGGAGCTCTTTGGAAGAAATTAGTGAAGCCACAGATGTAATTTCTTCCAAAGAGCTCCTGTTGCCTACTGCCTCGGA	Eurofins
1300017J02Rik.374	TGCTGTTGACAGTGAGCGCTGGCAGAATACTATGGATCAATAGTGAAGCCACAGATGTATTGATCCATAGTATTCTGCCATTGCCTACTGCCTCGGA	Eurofins
Gstm5.110	TGCTGTTGACAGTGAGCGACAAGTCTATGGTTCTGGGTTATAGTGAAGCCACAGATGTATAACCCAGAACCATAGACTTGGTGCCTACTGCCTCGGA	Eurofins
Gstm5.220	TGCTGTTGACAGTGAGCGAAAGCTCCTGACTATGATAGAATAGTGAAGCCACAGATGTATTCTATCATAGTCAGGAGCTTCTGCCTACTGCCTCGGA	Eurofins
Gstm5.225	TGCTGTTGACAGTGAGCGCCCTGACTATGATAGAAGCCAATAGTGAAGCCACAGATGTATTGGCTTCTATCATAGTCAGGATGCCTACTGCCTCGGA	Eurofins
Gstm5.395	TGCTGTTGACAGTGAGCGCACGAGTAGACATCATGGAGAATAGTGAAGCCACAGATGTATTCTCCATGATGTCTACTCGTATGCCTACTGCCTCGGA	Eurofins
Gstm5.446	TGCTGTTGACAGTGAGCGCTCGCCTCTGCTACAATTCTAATAGTGAAGCCACAGATGTATTAGAATTGTAGCAGAGGCGAATGCCTACTGCCTCGGA	Eurofins
Trim10.1060	TGCTGTTGACAGTGAGCGAACGGATATCAGAAGCACTCTATAGTGAAGCCACAGATGTATAGAGTGCTTCTGATATCCGTCTGCCTACTGCCTCGGA	Eurofins
Trim10.2050	TGCTGTTGACAGTGAGCGACAGGTGAAGCCCTGTAATCAATAGTGAAGCCACAGATGTATTGATTACAGGGCTTCACCTGGTGCCTACTGCCTCGGA	Eurofins
Trim10.2069	TGCTGTTGACAGTGAGCGAAACCAGCTGTGTGATTTCAAATAGTGAAGCCACAGATGTATTTGAAATCACACAGCTGGTTGTGCCTACTGCCTCGGA	Eurofins

Trim10.2072	TGCTGTTGACAGTGAGCGACAGCTGTGTGATTTCAAACAATAGTGAAGCCACAGATGTATTGTTTGAAATCACACAGCTGGTGCCTACTGCCTCGGA	Eurofins
Trim10.2183	TGCTGTTGACAGTGAGCGAAAAGTACACTATATACATTAATAGTGAAGCCACAGATGTATTAATGTATATAGTGTACTTTGTGCCTACTGCCTCGGA	Eurofins
Tmem56.1306	TGCTGTTGACAGTGAGCGCAAGGTTTAAACAAAAGTTGTATAGTGAAGCCACAGATGTATACAACTTTTGTTTAAACCTTTTGCCTACTGCCTCGGA	Eurofins
Tmem56.1973	TGCTGTTGACAGTGAGCGCCAAGTCAGAGTTTGTGCTTTATAGTGAAGCCACAGATGTATAAAGCACAAACTCTGACTTGTTGCCTACTGCCTCGGA	Eurofins
Tmem56.2076	TGCTGTTGACAGTGAGCGACCCAAGTATTCTGATTATCAATAGTGAAGCCACAGATGTATTGATAATCAGAATACTTGGGGTGCCTACTGCCTCGGA	Eurofins
Tmem56.3884	TGCTGTTGACAGTGAGCGCTAAGTCCAAGTTTCTGTTCAATAGTGAAGCCACAGATGTATTGAACAGAAACTTGGACTTAATGCCTACTGCCTCGGA	Eurofins
Tmem56.5215	TGCTGTTGACAGTGAGCGACACTAGATAGACTGTGTCATATAGTGAAGCCACAGATGTATATGACACAGTCTATCTAGTGGTGCCTACTGCCTCGGA	Eurofins
Slc25a21.1101	TGCTGTTGACAGTGAGCGCCAGCCTTTTAAGAATGTTTAATAGTGAAGCCACAGATGTATTAAACATTCTTAAAAGGCTGTTGCCTACTGCCTCGGA	Eurofins
Slc25a21.1448	TGCTGTTGACAGTGAGCGCAAGTCACATGTTGACATATAATAGTGAAGCCACAGATGTATTATATGTCAACATGTGACTTATGCCTACTGCCTCGGA	Eurofins
Slc25a21.675	TGCTGTTGACAGTGAGCGCACCGAAGCTGCTTCAAAACAATAGTGAAGCCACAGATGTATTGTTTTGAAGCAGCTTCGGTATGCCTACTGCCTCGGA	Eurofins
Slc25a21.83	TGCTGTTGACAGTGAGCGACCACAAAGTTATAGAACTGTATAGTGAAGCCACAGATGTATACAGTTCTATAACTTTGTGGGTGCCTACTGCCTCGGA	Eurofins
Slc25a21.84	TGCTGTTGACAGTGAGCGACACAAAGTTATAGAACTGTAATAGTGAAGCCACAGATGTATTACAGTTCTATAACTTTGTGGTGCCTACTGCCTCGGA	Eurofins
Hemgn.1011	TGCTGTTGACAGTGAGCGCCAAGAGATGGCTGTTCCAAAATAGTGAAGCCACAGATGTATTTTGGAACAGCCATCTCTTGATGCCTACTGCCTCGGA	Eurofins
Hemgn.143	TGCTGTTGACAGTGAGCGCTAGGCTGCTTGTGAAGTCAAATAGTGAAGCCACAGATGTATTTGACTTCACAAGCAGCCTAATGCCTACTGCCTCGGA	Eurofins
Hemgn.1866	TGCTGTTGACAGTGAGCGCAAGGCTCTAACACATAGACAATAGTGAAGCCACAGATGTATTGTCTATGTGTTAGAGCCTTATGCCTACTGCCTCGGA	Eurofins
Hemgn.2035	TGCTGTTGACAGTGAGCGCGAGGAGAAGATAGCAATTCAATAGTGAAGCCACAGATGTATTGAATTGCTATCTTCTCCTCATGCCTACTGCCTCGGA	Eurofins
Hemgn.2129	TGCTGTTGACAGTGAGCGCCAGAATTTTATTCGAGCTTTATAGTGAAGCCACAGATGTATAAAGCTCGAATAAAATTCTGATGCCTACTGCCTCGGA	Eurofins
Sgol1.1985	TGCTGTTGACAGTGAGCGACAGGAGAATTGCAGAGTACAATAGTGAAGCCACAGATGTATTGTACTCTGCAATTCTCCTGCTGCCTACTGCCTCGGA	Eurofins

Sgol1.2030	TGCTGTTGACAGTGAGCGCTAGGATGTAGATCTTTCATAATAGTGAAGCCACAGATGTATTATGAAAGATCTACATCCTATTGCCTACTGCCTCGGA	Eurofins
Sgol1.2254	TGCTGTTGACAGTGAGCGATCAGTCTACAGTGTAAATAAATAGTGAAGCCACAGATGTATTTATTTACACTGTAGACTGACTGCCTACTGCCTCGGA	Eurofins
Sgol1.382	TGCTGTTGACAGTGAGCGCCCCACTAACACTGCTACACTATAGTGAAGCCACAGATGTATAGTGTAGCAGTGTTAGTGGGTTGCCTACTGCCTCGGA	Eurofins
Sgol1.557	TGCTGTTGACAGTGAGCGCCCCGACAAAGTGAAGAAACTATAGTGAAGCCACAGATGTATAGTTTCTTCACTTTGTCGGGATGCCTACTGCCTCGGA	Eurofins
Slc22a23.1303	TGCTGTTGACAGTGAGCGACAGGAAAACGGTTCATAATTATAGTGAAGCCACAGATGTATAATTATGAACCGTTTTCCTGGTGCCTACTGCCTCGGA	Eurofins
Slc22a23.1814	TGCTGTTGACAGTGAGCGCAGCCATGTGTCTGGTAGTCAATAGTGAAGCCACAGATGTATTGACTACCAGACACATGGCTATGCCTACTGCCTCGGA	Eurofins
Slc22a23.3234	TGCTGTTGACAGTGAGCGACCGCCCGGTTTGCTTAGTTAATAGTGAAGCCACAGATGTATTAACTAAGCAAACCGGGCGGCTGCCTACTGCCTCGGA	Eurofins
Slc22a23.3948	TGCTGTTGACAGTGAGCGCAGGCAACAAAGTGACTTCTAATAGTGAAGCCACAGATGTATTAGAAGTCACTTTGTTGCCTATGCCTACTGCCTCGGA	Eurofins
Slc22a23.5070	TGCTGTTGACAGTGAGCGATACACCTGTTTTCATAAGTTATAGTGAAGCCACAGATGTATAACTTATGAAAACAGGTGTAGTGCCTACTGCCTCGGA	Eurofins
Cox6b2.138	TGCTGTTGACAGTGAGCGAACCGGTGTGTGAAGACCATGATAGTGAAGCCACAGATGTATCATGGTCTTCACACACCGGTGTGCCTACTGCCTCGGA	Eurofins
Cox6b2.139	TGCTGTTGACAGTGAGCGCCCGGTGTGTGAAGACCATGAATAGTGAAGCCACAGATGTATTCATGGTCTTCACACACCGGTTGCCTACTGCCTCGGA	Eurofins
Cox6b2.185	TGCTGTTGACAGTGAGCGATGCGAGTACTATTTCCGTGTATAGTGAAGCCACAGATGTATACACGGAAATAGTACTCGCAGTGCCTACTGCCTCGGA	Eurofins
Cox6b2.238	TGCTGTTGACAGTGAGCGCGCGCTGGAATGAGCAGATCAATAGTGAAGCCACAGATGTATTGATCTGCTCATTCCAGCGCTTGCCTACTGCCTCGGA	Eurofins
Cox6b2.327	TGCTGTTGACAGTGAGCGATAGTGGCTGTCCTTTCATCAATAGTGAAGCCACAGATGTATTGATGAAAGGACAGCCACTAGTGCCTACTGCCTCGGA	Eurofins
Sparc.1114	TGCTGTTGACAGTGAGCGATAGTGGCTGTCCTTTCATCAATAGTGAAGCCACAGATGTATTGATGAAAGGACAGCCACTAGTGCCTACTGCCTCGGA	Eurofins
Sparc.1195	TGCTGTTGACAGTGAGCGCTGAGACCTGTGACCTAGACAATAGTGAAGCCACAGATGTATTGTCTAGGTCACAGGTCTCAATGCCTACTGCCTCGGA	Eurofins
Sparc.1324	TGCTGTTGACAGTGAGCGCCAACAAGGATCTGGTGATCTATAGTGAAGCCACAGATGTATAGATCACCAGATCCTTGTTGATGCCTACTGCCTCGGA	Eurofins
Sparc.1529	TGCTGTTGACAGTGAGCGCAAGGTGCTAACATAGATTTAATAGTGAAGCCACAGATGTATTAAATCTATGTTAGCACCTTATGCCTACTGCCTCGGA	Eurofins

Sparc.1898	TGCTGTTGACAGTGAGCGAAAGCACGAGGAGATATCTCTATAGTGAAGCCACAGATGTATAGAGATATCTCCTCGTGCTTGTGCCTACTGCCTCGGA	Eurofins
Serpina3f.1121	TGCTGTTGACAGTGAGCGACAGAACATCACTCAACTGCAATAGTGAAGCCACAGATGTATTGCAGTTGAGTGATGTTCTGGTGCCTACTGCCTCGGA	Eurofins
Serpina3f.1122	TGCTGTTGACAGTGAGCGCCTCCAATGTTGTCAAGGTGTATAGTGAAGCCACAGATGTATACACCTTGACAACATTGGAGATGCCTACTGCCTCGGA	Eurofins
Serpina3f.1134	TGCTGTTGACAGTGAGCGATCCAATGTTGTCAAGGTGTAATAGTGAAGCCACAGATGTATTACACCTTGACAACATTGGAGTGCCTACTGCCTCGGA	Eurofins
Serpina3f.1801	TGCTGTTGACAGTGAGCGAAAGGTGTAATCTACTCTATGATAGTGAAGCCACAGATGTATCATAGAGTAGATTACACCTTGTGCCTACTGCCTCGGA	Eurofins
Serpina3f.432	TGCTGTTGACAGTGAGCGATGGGTCAGGGTAGTTCTTCTATAGTGAAGCCACAGATGTATAGAAGAACTACCCTGACCCACTGCCTACTGCCTCGGA	Eurofins
Il21.1509	TGCTGTTGACAGTGAGCGACAGAGTTCAAGGAGAAAGCAATAGTGAAGCCACAGATGTATTGCTTTCTCCTTGAACTCTGCTGCCTACTGCCTCGGA	Eurofins
Il21.154	TGCTGTTGACAGTGAGCGAAACCATCTATTTAAAATCAAATAGTGAAGCCACAGATGTATTTGATTTTAAATAGATGGTTGTGCCTACTGCCTCGGA	Eurofins
Il21.1656	TGCTGTTGACAGTGAGCGCCACCTTATTGACATTGTTGAATAGTGAAGCCACAGATGTATTCAACAATGTCAATAAGGTGATGCCTACTGCCTCGGA	Eurofins
Il21.1944	TGCTGTTGACAGTGAGCGAAACAGTATGTTTCAATGTCAATAGTGAAGCCACAGATGTATTGACATTGAAACATACTGTTGTGCCTACTGCCTCGGA	Eurofins
Il21.2242	TGCTGTTGACAGTGAGCGCAAGAACTTGTGTAACTGTAAATAGTGAAGCCACAGATGTATTTACAGTTACACAAGTTCTTTTGCCTACTGCCTCGGA	Eurofins
Fgg.1037	TGCTGTTGACAGTGAGCGCCAAGAAGTACAAATAATTCAATAGTGAAGCCACAGATGTATTGAATTATTTGTACTTCTTGATGCCTACTGCCTCGGA	Eurofins
Fgg.1365	TGCTGTTGACAGTGAGCGAAACGACAACGATAAGTTTGAATAGTGAAGCCACAGATGTATTCAAACTTATCGTTGTCGTTGTGCCTACTGCCTCGGA	Eurofins
Fgg.417	TGCTGTTGACAGTGAGCGCTCCAAGCAGAGAACTATACTATAGTGAAGCCACAGATGTATAGTATAGTTCTCTGCTTGGATTGCCTACTGCCTCGGA	Eurofins
Fgg.440	TGCTGTTGACAGTGAGCGCTACAGGAAATCTATAATTCAATAGTGAAGCCACAGATGTATTGAATTATAGATTTCCTGTAATGCCTACTGCCTCGGA	Eurofins
Fgg.442	TGCTGTTGACAGTGAGCGCAACCAGAAGATCACAAACCTATAGTGAAGCCACAGATGTATAGGTTTGTGATCTTCTGGTTATGCCTACTGCCTCGGA	Eurofins
Hp.1306	TGCTGTTGACAGTGAGCGCCCAGAAGATCACAAACCTAAATAGTGAAGCCACAGATGTATTTAGGTTTGTGATCTTCTGGTTGCCTACTGCCTCGGA	Eurofins
Hp.267	TGCTGTTGACAGTGAGCGACACTGCTCAGTCAACAATAAATAGTGAAGCCACAGATGTATTTATTGTTGACTGAGCAGTGCTGCCTACTGCCTCGGA	Eurofins

Hp.313	TGCTGTTGACAGTGAGCGACCCAGAGATTGCAAACGGCTATAGTGAAGCCACAGATGTATAGCCGTTTGCAATCTCTGGGGTGCCTACTGCCTCGGA	Eurofins
Hp.745	TGCTGTTGACAGTGAGCGATGCCGACAGTTCTACAGACTATAGTGAAGCCACAGATGTATAGTCTGTAGAACTGTCGGCAGTGCCTACTGCCTCGGA	Eurofins
Hp.756	TGCTGTTGACAGTGAGCGAAGGGTGCTTGTAACCGAGAGATAGTGAAGCCACAGATGTATCTCTCGGTTACAAGCACCCTCTGCCTACTGCCTCGGA	Eurofins
Mup20.128	TGCTGTTGACAGTGAGCGCAACCGAGAGAGTCATGCCTATTAGTGAAGCCACAGATGTAATAGGCATGACTCTCTCGGTTATGCCTACTGCCTCGGA	Eurofins
Mup20.234	TGCTGTTGACAGTGAGCGAAAGAAGCTAGTTCTATGGAAATAGTGAAGCCACAGATGTATTTCCATAGAACTAGCTTCTTCTGCCTACTGCCTCGGA	Eurofins
Mup20.281	TGCTGTTGACAGTGAGCGACAGCATGAGAGTTTTTGTCGATAGTGAAGCCACAGATGTATCGACAAAAACTCTCATGCTGCTGCCTACTGCCTCGGA	Eurofins
Mup20.353	TGCTGTTGACAGTGAGCGCTAGCTCTTAAATTCCATATTATAGTGAAGCCACAGATGTATAATATGGAATTTAAGAGCTAATGCCTACTGCCTCGGA	Eurofins
Mup20.758	TGCTGTTGACAGTGAGCGCAGGCTGGTGAATATTCTGTAATAGTGAAGCCACAGATGTATTACAGAATATTCACCAGCCTTTGCCTACTGCCTCGGA	Eurofins
Igsf9b.1045	TGCTGTTGACAGTGAGCGATCCAGCATGTTACTAGGATAATAGTGAAGCCACAGATGTATTATCCTAGTAACATGCTGGAGTGCCTACTGCCTCGGA	Eurofins
Igsf9b.1114	TGCTGTTGACAGTGAGCGCTCCAGAATGACCTGAAGCTAATAGTGAAGCCACAGATGTATTAGCTTCAGGTCATTCTGGAATGCCTACTGCCTCGGA	Eurofins
Igsf9b.177	TGCTGTTGACAGTGAGCGACAGAGGATGCTGGGAAGTATATAGTGAAGCCACAGATGTATATACTTCCCAGCATCCTCTGGTGCCTACTGCCTCGGA	Eurofins
Igsf9b.398	TGCTGTTGACAGTGAGCGAACGCAGGAGCACTTTGCGATATAGTGAAGCCACAGATGTATATCGCAAAGTGCTCCTGCGTCTGCCTACTGCCTCGGA	Eurofins
Igsf9b.494	TGCTGTTGACAGTGAGCGAACCCTATGTTGTAGAGTGGTTTAGTGAAGCCACAGATGTAAACCACTCTACAACATAGGGTGTGCCTACTGCCTCGGA	Eurofins
Rad51.1690	TGCTGTTGACAGTGAGCGACCGGGCCAGTCTTCATGATAATAGTGAAGCCACAGATGTATTATCATGAAGACTGGCCCGGCTGCCTACTGCCTCGGA	Eurofins
Rad51.1866	TGCTGTTGACAGTGAGCGCTACATGCAGCATGCTAGACTATAGTGAAGCCACAGATGTATAGTCTAGCATGCTGCATGTAATGCCTACTGCCTCGGA	Eurofins
Rad51.386	TGCTGTTGACAGTGAGCGCTCGGTACTGTGTTGTTCGTTATAGTGAAGCCACAGATGTATAACGAACAACACAGTACCGAATGCCTACTGCCTCGGA	Eurofins
Rad51.506	TGCTGTTGACAGTGAGCGAACCGAAGAAGGAACTAATAAATAGTGAAGCCACAGATGTATTTATTAGTTCCTTCTTCGGTGTGCCTACTGCCTCGGA	Eurofins
Rad51.764	TGCTGTTGACAGTGAGCGACCGGTCAGAGATCATACAGATTAGTGAAGCCACAGATGTAATCTGTATGATCTCTGACCGGCTGCCTACTGCCTCGGA	Eurofins

Phactr2.2036	TGCTGTTGACAGTGAGCGATGGCAGCGATGTCCTAGATAATAGTGAAGCCACAGATGTATTATCTAGGACATCGCTGCCAGTGCCTACTGCCTCGGA	Eurofins
Phactr2.2439	TGCTGTTGACAGTGAGCGCCAGGGTGAGAGCTAAAACTAATAGTGAAGCCACAGATGTATTAGTTTTAGCTCTCACCCTGTTGCCTACTGCCTCGGA	Eurofins
Phactr2.4266	TGCTGTTGACAGTGAGCGCCAGGAATACGGTCAGATGTAATAGTGAAGCCACAGATGTATTACATCTGACCGTATTCCTGTTGCCTACTGCCTCGGA	Eurofins
Phactr2.4267	TGCTGTTGACAGTGAGCGACAAGCTGTAGTTCTCAGAATATAGTGAAGCCACAGATGTATATTCTGAGAACTACAGCTTGGTGCCTACTGCCTCGGA	Eurofins
Phactr2.5877	TGCTGTTGACAGTGAGCGAAAGCTGTAGTTCTCAGAATAATAGTGAAGCCACAGATGTATTATTCTGAGAACTACAGCTTGTGCCTACTGCCTCGGA	Eurofins
Cd200r4.1007	TGCTGTTGACAGTGAGCGACAGGAAGATGCAGAAGCTTAATAGTGAAGCCACAGATGTATTAAGCTTCTGCATCTTCCTGCTGCCTACTGCCTCGGA	Eurofins
Cd200r4.1012	TGCTGTTGACAGTGAGCGCTAGTCTGAGGACACAATACAATAGTGAAGCCACAGATGTATTGTATTGTGTCCTCAGACTAATGCCTACTGCCTCGGA	Eurofins
Cd200r4.1242	TGCTGTTGACAGTGAGCGATGAGGACACAATACAACAAAATAGTGAAGCCACAGATGTATTTTGTTGTATTGTGTCCTCAGTGCCTACTGCCTCGGA	Eurofins
Cd200r4.409	TGCTGTTGACAGTGAGCGCTGGGAGATATGTCTAGCAGTTTAGTGAAGCCACAGATGTAAACTGCTAGACATATCTCCCAATGCCTACTGCCTCGGA	Eurofins
Cd200r4.55	TGCTGTTGACAGTGAGCGATTCCTGCACAATAGCATACAATAGTGAAGCCACAGATGTATTGTATGCTATTGTGCAGGAAGTGCCTACTGCCTCGGA	Eurofins
Sytl2.1219	TGCTGTTGACAGTGAGCGCGAGGAAGAAAGTCACTCCTAATAGTGAAGCCACAGATGTATTAGGAGTGACTTTCTTCCTCATGCCTACTGCCTCGGA	Eurofins
Sytl2.1290	TGCTGTTGACAGTGAGCGCCCCGTTGTCACTAATGACTTTTAGTGAAGCCACAGATGTAAAAGTCATTAGTGACAACGGGATGCCTACTGCCTCGGA	Eurofins
Sytl2.1333	TGCTGTTGACAGTGAGCGAAAGGGAGTTAAACGATGACAATAGTGAAGCCACAGATGTATTGTCATCGTTTAACTCCCTTGTGCCTACTGCCTCGGA	Eurofins
Sytl2.1556	TGCTGTTGACAGTGAGCGATGGCGTGATGCCGACATTCAATAGTGAAGCCACAGATGTATTGAATGTCGGCATCACGCCACTGCCTACTGCCTCGGA	Eurofins
Sytl2.1882	TGCTGTTGACAGTGAGCGAAGCGGTGAAGACAATGACCTATAGTGAAGCCACAGATGTATAGGTCATTGTCTTCACCGCTGTGCCTACTGCCTCGGA	Eurofins
Coro2a.1531	TGCTGTTGACAGTGAGCGCCAGCATTAAAGTATTCACATATAGTGAAGCCACAGATGTATATGTGAATACTTTAATGCTGTTGCCTACTGCCTCGGA	Eurofins
Coro2a.252	TGCTGTTGACAGTGAGCGACAGCTGGAACTAGAGATCAAATAGTGAAGCCACAGATGTATTTGATCTCTAGTTCCAGCTGCTGCCTACTGCCTCGGA	Eurofins
Coro2a.2590	TGCTGTTGACAGTGAGCGAAGGGAACGTCTTGGACATCAATAGTGAAGCCACAGATGTATTGATGTCCAAGACGTTCCCTCTGCCTACTGCCTCGGA	Eurofins

Coro2a.3389	TGCTGTTGACAGTGAGCGAACGCTTTTAGCTAGCAT CCTATAGTGAAGCCACAGATGTATAGGATGCTAGCT AAAAGCGTCTGCCTACTGCCTCGGA	Eurofins
Coro2a.516	TGCTGTTGACAGTGAGCGCCCCACCTTAGCTCTACA ATAATAGTGAAGCCACAGATGTATTATTGTAGAGCTA AGGTGGGTTGCCTACTGCCTCGGA	Eurofins
Ms4a3.140	TGCTGTTGACAGTGAGCGATGGGCCAGTGAAGACAA TCAATAGTGAAGCCACAGATGTATTGATTGTCTTCAC TGGCCCAGTGCCTACTGCCTCGGA	Eurofins
Ms4a3.262	TGCTGTTGACAGTGAGCGAAAGGACTTGTCAAATCC TGAATAGTGAAGCCACAGATGTATTCAGGATTTGACA AGTCCTTCTGCCTACTGCCTCGGA	Eurofins
Ms4a3.29	TGCTGTTGACAGTGAGCGCCGCCATGTTCAACGAGG TGTATAGTGAAGCCACAGATGTATACACCTCGTTGAA CATGGCGTTGCCTACTGCCTCGGA	Eurofins
Ms4a3.480	TGCTGTTGACAGTGAGCGACACAAAGAAGAGATTCG TTAATAGTGAAGCCACAGATGTATTAACGAATCTCTT CTTTGTGCTGCCTACTGCCTCGGA	Eurofins
Ms4a3.728	TGCTGTTGACAGTGAGCGACACACGAATGCTGATGC AAAATAGTGAAGCCACAGATGTATTTTGCATCAGCAT TCGTGTGGTGCCTACTGCCTCGGA	Eurofins
Gc.1250	TGCTGTTGACAGTGAGCGCTGGGAAATGTTTGTGGT TTAATAGTGAAGCCACAGATGTATTAAACCACAAACA TTTCCCAATGCCTACTGCCTCGGA	Eurofins
Gc.1574	TGCTGTTGACAGTGAGCGCCCAGAAGTGTTCCTCAG CAAATAGTGAAGCCACAGATGTATTTGCTGAGGAAC ACTTCTGGATGCCTACTGCCTCGGA	Eurofins
Gc.1702	TGCTGTTGACAGTGAGCGCCAGATTGATGCAGAAAT GATATAGTGAAGCCACAGATGTATATCATTTCTGCAT CAATCTGTTGCCTACTGCCTCGGA	Eurofins
Gc.1740	TGCTGTTGACAGTGAGCGCTCCAAGAAGATCACCAA GATATAGTGAAGCCACAGATGTATATCTTGGTGATCT TCTTGGAATGCCTACTGCCTCGGA	Eurofins
Gc.241	TGCTGTTGACAGTGAGCGATACAATGTTTTTGGAGAA TTATAGTGAAGCCACAGATGTATAATTCTCCAAAAAC ATTGTAGTGCCTACTGCCTCGGA	Eurofins
Serpina1c.1172	TGCTGTTGACAGTGAGCGACCGAGACTATGAGAAGG ATAATAGTGAAGCCACAGATGTATTATCCTTCTCATA GTCTCGGCTGCCTACTGCCTCGGA	Eurofins
Serpina1c.17	TGCTGTTGACAGTGAGCGAATGGATGAGACAGGAAC AGAATAGTGAAGCCACAGATGTATTCTGTTCCTGTCT CATCCATGTGCCTACTGCCTCGGA	Eurofins
Serpina1c.217	TGCTGTTGACAGTGAGCGACCACTACTTAAATACAG ACTATAGTGAAGCCACAGATGTATAGTCTGTATTTAA GTAGTGGGTGCCTACTGCCTCGGA	Eurofins
Serpina1c.390	TGCTGTTGACAGTGAGCGACTCCCATGAGATCGCTA CAAATAGTGAAGCCACAGATGTATTTGTAGCGATCTC ATGGGAGGTGCCTACTGCCTCGGA	Eurofins
Serpina1c.952	TGCTGTTGACAGTGAGCGATGCAGTTCAACCTCACA CAAATAGTGAAGCCACAGATGTATTTGTGTGAGGTT GAACTGCAGTGCCTACTGCCTCGGA	Eurofins
Serpina1b.1408	TGCTGTTGACAGTGAGCGCCAGCAAGGAGCTCATCT CCAATAGTGAAGCCACAGATGTATTGGAGATGAGCT CCTTGCTGATGCCTACTGCCTCGGA	Eurofins

Serpina1b.18	TGCTGTTGACAGTGAGCGAGTGCCTGATGCTACAGCAAAATAGTGAAGCCACAGATGTATTTTGCTGTAGCATCAGGCACGTGCCTACTGCCTCGGA	Eurofins
Serpina1b.21	TGCTGTTGACAGTGAGCGACCACTGCTTAAATACAGACTATAGTGAAGCCACAGATGTATAGTCTGTATTTAAGCAGTGGGTGCCTACTGCCTCGGA	Eurofins
Serpina1b.711	TGCTGTTGACAGTGAGCGCCTGCTTAAATACAGACTAGGATAGTGAAGCCACAGATGTATCCTAGTCTGTATTTAAGCAGTTGCCTACTGCCTCGGA	Eurofins
Serpina1b.965	TGCTGTTGACAGTGAGCGCTACATTCTTTTTAAAGGCAAATAGTGAAGCCACAGATGTATTTGCCTTTAAAAAGAATGTAATGCCTACTGCCTCGGA	Eurofins
S100a9.162	TGCTGTTGACAGTGAGCGCCATCTCTAAGATCCTGCTAAATAGTGAAGCCACAGATGTATTTAGCAGGATCTTAGAGATGATGCCTACTGCCTCGGA	Eurofins
S100a9.175	TGCTGTTGACAGTGAGCGAAAGCACAGTTGGCAACCTTTATAGTGAAGCCACAGATGTATAAAGGTTGCCAACTGTGCTTCTGCCTACTGCCTCGGA	Eurofins
S100a9.185	TGCTGTTGACAGTGAGCGAAACCTTTATGAAGAAAGAGAATAGTGAAGCCACAGATGTATTCTCTTTCTTCATAAAGGTTGTGCCTACTGCCTCGGA	Eurofins
S100a9.25	TGCTGTTGACAGTGAGCGAAAGAAAGAGAAGAGAAATGAATAGTGAAGCCACAGATGTATTCATTTCTCTTCTCTTTCTTCTGCCTACTGCCTCGGA	Eurofins
S100a9.360	TGCTGTTGACAGTGAGCGCGAGCAAGAAGATGGCCAACAATAGTGAAGCCACAGATGTATTGTTGGCCATCTTCTTGCTCATGCCTACTGCCTCGGA	Eurofins
Hba-a1.122	TGCTGTTGACAGTGAGCGCAAGGCTGTGGGAAGTAATTAATAGTGAAGCCACAGATGTATTAATTACTTCCCACAGCCTTTTGCCTACTGCCTCGGA	Eurofins
Hba-a1.14	TGCTGTTGACAGTGAGCGACCCTGGAAAGGATGTTTGCTATAGTGAAGCCACAGATGTATAGCAAACATCCTTTCCAGGGCTGCCTACTGCCTCGGA	Eurofins
Hba-a1.156	TGCTGTTGACAGTGAGCGCTCTGACAGACTCAGGAAGAAATAGTGAAGCCACAGATGTATTTCTTCCTGAGTCTGTCAGAATGCCTACTGCCTCGGA	Eurofins
Hba-a1.523	TGCTGTTGACAGTGAGCGACAAGACCTACTTCCCTCACTTTAGTGAAGCCACAGATGTAAAGTGAGGGAAGTAGGTCTTGGTGCCTACTGCCTCGGA	Eurofins
Hba-a1.541	TGCTGTTGACAGTGAGCGATACCTCTTGGTCTTTGAATAATAGTGAAGCCACAGATGTATTATTCAAAGACCAAGAGGTACTGCCTACTGCCTCGGA	Eurofins
Hba-a2.10	TGCTGTTGACAGTGAGCGCTAAAGCCTGAGTAGGAAGAAATAGTGAAGCCACAGATGTATTTCTTCCTACTCAGGCTTTATTGCCTACTGCCTCGGA	Eurofins
Hba-a2.11	TGCTGTTGACAGTGAGCGCTTCTGACAGACTCAGGAAGAATAGTGAAGCCACAGATGTATTCTTCCTGAGTCTGTCAGAATTGCCTACTGCCTCGGA	Eurofins
Hba-a2.119	TGCTGTTGACAGTGAGCGCTCTGACAGACTCAGGAAGAAATAGTGAAGCCACAGATGTATTTCTTCCTGAGTCTGTCAGAATGCCTACTGCCTCGGA	Eurofins
Hba-a2.153	TGCTGTTGACAGTGAGCGACCCTGGAAAGGATGTTTGCTATAGTGAAGCCACAGATGTATAGCAAACATCCTTTCCAGGGCTGCCTACTGCCTCGGA	Eurofins

Hba-a2.517	TGCTGTTGACAGTGAGCGACAAGACCTACTTCCCTCACTTTAGTGAAGCCACAGATGTAAAGTGAGGGAAGTAGGTCTTGGTGCCTACTGCCTCGGA	Eurofins
Lcn2.230	TGCTGTTGACAGTGAGCGACTGTACCTCTTGGTCTTTGAATAGTGAAGCCACAGATGTATTCAAAGACCAAGAGGTACAGGTGCCTACTGCCTCGGA	Eurofins
Lcn2.242	TGCTGTTGACAGTGAGCGCTACGATGTACAGCACCATCTATAGTGAAGCCACAGATGTATAGATGGTGCTGTACATCGTAATGCCTACTGCCTCGGA	Eurofins
Lcn2.258	TGCTGTTGACAGTGAGCGACACCATCTATGAGCTACAAGATAGTGAAGCCACAGATGTATCTTGTAGCTCATAGATGGTGCTGCCTACTGCCTCGGA	Eurofins
Lcn2.269	TGCTGTTGACAGTGAGCGCCAAGAGAACAATAGCTACAATTAGTGAAGCCACAGATGTAATTGTAGCTATTGTTCTCTTGTTGCCTACTGCCTCGGA	Eurofins
Lcn2.458	TGCTGTTGACAGTGAGCGCTAGCTACAATGTCACCTCCATTAGTGAAGCCACAGATGTAATGGAGGTGACATTGTAGCTATTGCCTACTGCCTCGGA	Eurofins
Ngp.140	TGCTGTTGACAGTGAGCGAGAGATTGTTGATAGAGCCATATAGTGAAGCCACAGATGTATATGGCTCTATCAACAATCTCCTGCCTACTGCCTCGGA	Eurofins
Ngp.270	TGCTGTTGACAGTGAGCGCAAGAGACAGAGTGTACTTCCATAGTGAAGCCACAGATGTATGGAAGTACACTCTGTCTCTTTTGCCTACTGCCTCGGA	Eurofins
Ngp.434	TGCTGTTGACAGTGAGCGCACCAGTTTTAATGATAAGCAATAGTGAAGCCACAGATGTATTGCTTATCATTAAAACTGGTTTGCCTACTGCCTCGGA	Eurofins
Ngp.452	TGCTGTTGACAGTGAGCGACAAGACGTCTCTGAAAAGGAATAGTGAAGCCACAGATGTATTCCTTTTCAGAGACGTCTTGCTGCCTACTGCCTCGGA	Eurofins
Ngp.859	TGCTGTTGACAGTGAGCGCCAGTTCACTCATAATCTTCAATAGTGAAGCCACAGATGTATTGAAGATTATGAGTGAACTGATGCCTACTGCCTCGGA	Eurofins
Penk.1057	TGCTGTTGACAGTGAGCGATCGAAAGAAGTTCCTGAGATATAGTGAAGCCACAGATGTATATCTCAGGAACTTCTTTCGAGTGCCTACTGCCTCGGA	Eurofins
Penk.1194	TGCTGTTGACAGTGAGCGCCACATGCTGCTTTGTGCTGTATAGTGAAGCCACAGATGTATACAGCACAAAGCAGCATGTGATGCCTACTGCCTCGGA	Eurofins
Penk.1223	TGCTGTTGACAGTGAGCGACCCGTGGTCTAGATAACTACATAGTGAAGCCACAGATGTATGTAGTTATCTAGACCACGGGGTGCCTACTGCCTCGGA	Eurofins
Penk.1290	TGCTGTTGACAGTGAGCGAAAGCTCAGTATTGGTCTCTTATAGTGAAGCCACAGATGTATAAGAGACCAATACTGAGCTTCTGCCTACTGCCTCGGA	Eurofins
Penk.763	TGCTGTTGACAGTGAGCGATCCGATCTGCTGAAAGAGCTATAGTGAAGCCACAGATGTATAGCTCTTTCAGCAGATCGGAGTGCCTACTGCCTCGGA	Eurofins
S100a8.102	TGCTGTTGACAGTGAGCGATACCACAATTATTCCAATATATAGTGAAGCCACAGATGTATATATTGGAATAATTGTGGTAGTGCCTACTGCCTCGGA	Eurofins
S100a8.105	TGCTGTTGACAGTGAGCGACACAATTATTCCAATATACAATAGTGAAGCCACAGATGTATTGTATATTGGAATAATTGTGGTGCCTACTGCCTCGGA	Eurofins

S100a8.144	TGCTGTTGACAGTGAGCGATACAAGAATGACTTCAAGAAATAGTGAAGCCACAGATGTATTTCTTGAAGTCATTCTTGTAGTGCCTACTGCCTCGGA	Eurofins
S100a8.185	TGCTGTTGACAGTGAGCGATCAGTTTGTGCAGAATATAAATAGTGAAGCCACAGATGTATTTATATTCTGCACAAACTGAGTGCCTACTGCCTCGGA	Eurofins
S100a8.351	TGCTGTTGACAGTGAGCGATCCCTGGATATGTCTACAGAATAGTGAAGCCACAGATGTATTCTGTAGACATATCCAGGGACTGCCTACTGCCTCGGA	Eurofins
Alb.1055	TGCTGTTGACAGTGAGCGCCCAGGAAGTGTGCAAGAACTATAGTGAAGCCACAGATGTATAGTTCTTGCACACTTCCTGGTTGCCTACTGCCTCGGA	Eurofins
Alb.1056	TGCTGTTGACAGTGAGCGACAGGAAGTGTGCAAGAACTATTAGTGAAGCCACAGATGTAATAGTTCTTGCACACTTCCTGGTGCCTACTGCCTCGGA	Eurofins
Alb.1674	TGCTGTTGACAGTGAGCGAAAGGAGAAGCAGATTAAGAAATAGTGAAGCCACAGATGTATTTCTTAATCTGCTTCTCCTTCTGCCTACTGCCTCGGA	Eurofins
Alb.1839	TGCTGTTGACAGTGAGCGCAACCTTGTCACTAGATGCAAATAGTGAAGCCACAGATGTATTTGCATCTAGTGACAAGGTTTTGCCTACTGCCTCGGA	Eurofins
Alb.225	TGCTGTTGACAGTGAGCGCTACGATGAGCATGCCAAATTATAGTGAAGCCACAGATGTATAATTTGGCATGCTCATCGTATTGCCTACTGCCTCGGA	Eurofins
Mup3.144	TGCTGTTGACAGTGAGCGAATGCAGAAGAATCTAGTTCTATAGTGAAGCCACAGATGTATAGAACTAGATTCTTCTGCATGTGCCTACTGCCTCGGA	Eurofins
Mup3.150	TGCTGTTGACAGTGAGCGAAAGAATCTAGTTCTATGGAAATAGTGAAGCCACAGATGTATTTCCATAGAACTAGATTCTTCTGCCTACTGCCTCGGA	Eurofins
Mup3.314	TGCTGTTGACAGTGAGCGCTTCCATTTAATTGTAAATGAATAGTGAAGCCACAGATGTATTCATTTACAATTAAATGGAATTGCCTACTGCCTCGGA	Eurofins
Mup3.347	TGCTGTTGACAGTGAGCGCATGACTGCGATTGGTGAACAATAGTGAAGCCACAGATGTATTGTTCACCAATCGCAGTCATTTGCCTACTGCCTCGGA	Eurofins
Mup3.742	TGCTGTTGACAGTGAGCGCCTCCACCTGTCTCACAGAGAATAGTGAAGCCACAGATGTATTCTCTGTGAGACAGGTGGAGATGCCTACTGCCTCGGA	Eurofins
Rhag.1325	TGCTGTTGACAGTGAGCGACACGTGGAACATGAAGTCTAATAGTGAAGCCACAGATGTATTAGACTTCATGTTCCACGTGGTGCCTACTGCCTCGGA	Eurofins
Rhag.1630	TGCTGTTGACAGTGAGCGCGAGATGAATTATAGAAATCAATAGTGAAGCCACAGATGTATTGATTTCTATAATTCATCTCTTGCCTACTGCCTCGGA	Eurofins
Rhag.2179	TGCTGTTGACAGTGAGCGCAACAGTCATAGTAATGTTATATAGTGAAGCCACAGATGTATATAACATTACTATGACTGTTTTGCCTACTGCCTCGGA	Eurofins
Rhag.221	TGCTGTTGACAGTGAGCGACAAGATGTGCATGTCATGATATAGTGAAGCCACAGATGTATATCATGACATGCACATCTTGGTGCCTACTGCCTCGGA	Eurofins
Rhag.521	TGCTGTTGACAGTGAGCGAAACGAATATCTTGTTACTGAATAGTGAAGCCACAGATGTATTCAGTAACAAGATATTCGTTGTGCCTACTGCCTCGGA	Eurofins

Fam55b.1720	TGCTGTTGACAGTGAGCGCTAGAGAGACTGTTCTTACGAATAGTGAAGCCACAGATGTATTCGTAAGAACAGTCTCTCTATTGCCTACTGCCTCGGA	Eurofins
Fam55b.1745	TGCTGTTGACAGTGAGCGACGAGACCAAGGTGATAATTAATAGTGAAGCCACAGATGTATTAATTATCACCTTGGTCTCGGTGCCTACTGCCTCGGA	Eurofins
Fam55b.2557	TGCTGTTGACAGTGAGCGATGGAGAGAGTTACACAGTTAATAGTGAAGCCACAGATGTATTAACTGTGTAACTCTCTCCACTGCCTACTGCCTCGGA	Eurofins
Fam55b.2596	TGCTGTTGACAGTGAGCGCAAGCTTGATGTTTAAATAGTATAGTGAAGCCACAGATGTATACTATTTAAACATCAAGCTTATGCCTACTGCCTCGGA	Eurofins
Fam55b.3320	TGCTGTTGACAGTGAGCGCGAGCTTTAGATTAGACTCTAATAGTGAAGCCACAGATGTATTAGAGTCTAATCTAAAGCTCTTGCCTACTGCCTCGGA	Eurofins
Ahsp.105	TGCTGTTGACAGTGAGCGACACAGGGATAAAGGAGTTTAATAGTGAAGCCACAGATGTATTAAACTCCTTTATCCCTGTGGTGCCTACTGCCTCGGA	Eurofins
Ahsp.303	TGCTGTTGACAGTGAGCGACAGCCAGTTTCTAGCCAAATATAGTGAAGCCACAGATGTATATTTGGCTAGAAACTGGCTGCTGCCTACTGCCTCGGA	Eurofins
Ahsp.324	TGCTGTTGACAGTGAGCGCCAGGACCTTTCTGAAGTCCAATAGTGAAGCCACAGATGTATTGGACTTCAGAAAGGTCCTGTTGCCTACTGCCTCGGA	Eurofins
Ahsp.449	TGCTGTTGACAGTGAGCGATCCCAAGATGTTGCTGTACTTTAGTGAAGCCACAGATGTAAAGTACAGCAACATCTTGGGAGTGCCTACTGCCTCGGA	Eurofins
Ahsp.452	TGCTGTTGACAGTGAGCGACAAGATGTTGCTGTACTTTGATAGTGAAGCCACAGATGTATCAAAGTACAGCAACATCTTGGTGCCTACTGCCTCGGA	Eurofins
Car1.113	TGCTGTTGACAGTGAGCGACAGTCTCCTATTGATATTAAATAGTGAAGCCACAGATGTATTTAATATCAATAGGAGACTGGTGCCTACTGCCTCGGA	Eurofins
Car1.216	TGCTGTTGACAGTGAGCGATGGGACATTCTTTCCATGTAATAGTGAAGCCACAGATGTATTACATGGAAAGAATGTCCCACTGCCTACTGCCTCGGA	Eurofins
Car1.503	TGCTGTTGACAGTGAGCGACAGAAAGTACTTGATGCTCTATAGTGAAGCCACAGATGTATAGAGCATCAAGTACTTTCTGCTGCCTACTGCCTCGGA	Eurofins
Car1.819	TGCTGTTGACAGTGAGCGACAGCAAGGAATGAGTCCTCAATAGTGAAGCCACAGATGTATTGAGGACTCATTCCTTGCTGGTGCCTACTGCCTCGGA	Eurofins
Car1.882	TGCTGTTGACAGTGAGCGCCACGATTAAGAAACAGAATTATAGTGAAGCCACAGATGTATAATTCTGTTTCTTAATCGTGATGCCTACTGCCTCGGA	Eurofins
Ear12.24	TGCTGTTGACAGTGAGCGCCCCGACTTTGTCTCCTGTTGTTAGTGAAGCCACAGATGTAACAACAGGAGACAAAGTCGGGATGCCTACTGCCTCGGA	Eurofins
Ear12.25	TGCTGTTGACAGTGAGCGACCGACTTTGTCTCCTGTTGTTTAGTGAAGCCACAGATGTAAACAACAGGAGACAAAGTCGGGTGCCTACTGCCTCGGA	Eurofins
Ear12.328	TGCTGTTGACAGTGAGCGACAGTCGGAGAAGAACACCTTATAGTGAAGCCACAGATGTATAAGGTGTTCTTCTCCGACTGGTGCCTACTGCCTCGGA	Eurofins

Ear12.330	TGCTGTTGACAGTGAGCGCGTCGGAGAAGAACACCTTATATAGTGAAGCCACAGATGTATATAAGGTGTTCTTCTCCGACTTGCCTACTGCCTCGGA	Eurofins
Ear12.35	TGCTGTTGACAGTGAGCGCCTCCTGTTGTTGCTAGGACTTTAGTGAAGCCACAGATGTAAAGTCCTAGCAACAACAGGAGATGCCTACTGCCTCGGA	Eurofins
Ear2.202	TGCTGTTGACAGTGAGCGAAACCTCCAATGTAATGTTGAATAGTGAAGCCACAGATGTATTCAACATTACATTGGAGGTTGTGCCTACTGCCTCGGA	Eurofins
Ear2.237	TGCTGTTGACAGTGAGCGCCAGGTTTAGAAGAACATGTAATAGTGAAGCCACAGATGTATTACATGTTCTTCTAAACCTGTTGCCTACTGCCTCGGA	Eurofins
Ear2.328	TGCTGTTGACAGTGAGCGATGCAGTGACAATATAAGTAGATAGTGAAGCCACAGATGTATCTACTTATATTGTCACTGCACTGCCTACTGCCTCGGA	Eurofins
Ear2.341	TGCTGTTGACAGTGAGCGCTAAGTAGAAACTGTCATAATATAGTGAAGCCACAGATGTATATTATGACAGTTTCTACTTATTGCCTACTGCCTCGGA	Eurofins
Ear2.422	TGCTGTTGACAGTGAGCGCCCCAATGCAGATACCAACCAATAGTGAAGCCACAGATGTATTGGTTGGTATCTGCATTGGGTTGCCTACTGCCTCGGA	Eurofins
Ear3.131	TGCTGTTGACAGTGAGCGAAACCTCCGATGTAATGTTGAATAGTGAAGCCACAGATGTATTCAACATTACATCGGAGGTTGTGCCTACTGCCTCGGA	Eurofins
Ear3.136	TGCTGTTGACAGTGAGCGCCCGATGTAATGTTGAAATGCTTAGTGAAGCCACAGATGTAAGCATTTCAACATTACATCGGATGCCTACTGCCTCGGA	Eurofins
Ear3.144	TGCTGTTGACAGTGAGCGCATGTTGAAATGCTGCGTATTATAGTGAAGCCACAGATGTATAATACGCAGCATTTCAACATTTGCCTACTGCCTCGGA	Eurofins
Ear3.328	TGCTGTTGACAGTGAGCGACAGTCGGAGGAGAATACCTTATAGTGAAGCCACAGATGTATAAGGTATTCTCCTCCGACTGGTGCCTACTGCCTCGGA	Eurofins
Ear3.335	TGCTGTTGACAGTGAGCGAAGGAGAATACCTTATACCCAATAGTGAAGCCACAGATGTATTGGGTATAAGGTATTCTCCTCTGCCTACTGCCTCGGA	Eurofins
Ccl1.198	TGCTGTTGACAGTGAGCGACCTGAAGTTTATCCAGTGTTATAGTGAAGCCACAGATGTATAACACTGGATAAACTTCAGGGTGCCTACTGCCTCGGA	Eurofins
Ccl1.265	TGCTGTTGACAGTGAGCGAAGGCTGAACAAAGGTAGAGAATAGTGAAGCCACAGATGTATTCTCTACCTTTGTTCAGCCTGTGCCTACTGCCTCGGA	Eurofins
Ccl1.44	TGCTGTTGACAGTGAGCGCGAGGCTTGAGACAGAAACTTATAGTGAAGCCACAGATGTATAAGTTTCTGTCTCAAGCCTCTTGCCTACTGCCTCGGA	Eurofins
Ccl1.450	TGCTGTTGACAGTGAGCGAACAGACCTCAGTCATGTGATATAGTGAAGCCACAGATGTATATCACATGACTGAGGTCTGTGTGCCTACTGCCTCGGA	Eurofins
Ccl1.470	TGCTGTTGACAGTGAGCGCAAGCTATGTTATGGTTCTTTATAGTGAAGCCACAGATGTATAAAGAACCATAACATAGCTTATGCCTACTGCCTCGGA	Eurofins
Mpo.1116	TGCTGTTGACAGTGAGCGAACCCTCGAATCAAGAACCAAATAGTGAAGCCACAGATGTATTTGGTTCTTGATTCGAGGGTCTGCCTACTGCCTCGGA	Eurofins

Mpo.1922	TGCTGTTGACAGTGAGCGACGGGAGCGACTATTTGAGCAATAGTGAAGCCACAGATGTATTGCTCAAATAGTCGCTCCCGGTGCCTACTGCCTCGGA	Eurofins
Mpo.2098	TGCTGTTGACAGTGAGCGAACGGAAGCTGATGGCACAATATAGTGAAGCCACAGATGTATATTGTGCCATCAGCTTCCGTGTGCCTACTGCCTCGGA	Eurofins
Mpo.2344	TGCTGTTGACAGTGAGCGCCACCACCGTGTCGAAGAACAATAGTGAAGCCACAGATGTATTGTTCTTCGACACGGTGGTGATGCCTACTGCCTCGGA	Eurofins
Mpo.2388	TGCTGTTGACAGTGAGCGACCCGAGACTTTGTCAGCTGTATAGTGAAGCCACAGATGTATACAGCTGACAAAGTCTCGGGGTGCCTACTGCCTCGGA	Eurofins
Ctsg.106	TGCTGTTGACAGTGAGCGACGGCAGCAACTGACTAAGCAATAGTGAAGCCACAGATGTATTGCTTAGTCAGTTGCTGCCGGTGCCTACTGCCTCGGA	Eurofins
Ctsg.300	TGCTGTTGACAGTGAGCGATGCGAGAAGACTTCGTCCTAATAGTGAAGCCACAGATGTATTAGGACGAAGTCTTCTCGCACTGCCTACTGCCTCGGA	Eurofins
Ctsg.321	TGCTGTTGACAGTGAGCGCCAGCAGCTCATTGCTTGGGAATAGTGAAGCCACAGATGTATTCCCAAGCAATGAGCTGCTGTTGCCTACTGCCTCGGA	Eurofins
Ctsg.336	TGCTGTTGACAGTGAGCGCTGGGAAGCTCCATAAATGTTATAGTGAAGCCACAGATGTATAACATTTATGGAGCTTCCCAATGCCTACTGCCTCGGA	Eurofins
Ctsg.341	TGCTGTTGACAGTGAGCGCAGCTCCATAAATGTTACTCTATAGTGAAGCCACAGATGTATAGAGTAACATTTATGGAGCTTTGCCTACTGCCTCGGA	Eurofins
Cacna1d.2728	TGCTGTTGACAGTGAGCGCTACGGTTGAAATCCTGTTAAATAGTGAAGCCACAGATGTATTTAACAGGATTTCAACCGTAATGCCTACTGCCTCGGA	Eurofins
Cacna1d.4611	TGCTGTTGACAGTGAGCGATCCGAGCTGTGATCAAGAAAATAGTGAAGCCACAGATGTATTTTCTTGATCACAGCTCGGAGTGCCTACTGCCTCGGA	Eurofins
Cacna1d.4914	TGCTGTTGACAGTGAGCGAAAGAAGATGTATTCAAAAGAATAGTGAAGCCACAGATGTATTCTTTTGAATACATCTTCTTCTGCCTACTGCCTCGGA	Eurofins
Cacna1d.6892	TGCTGTTGACAGTGAGCGCCCGGATCGTTTTGTTAAGCGATAGTGAAGCCACAGATGTATCGCTTAACAAAACGATCCGGTTGCCTACTGCCTCGGA	Eurofins
Cacna1d.6943	TGCTGTTGACAGTGAGCGAACGGTCAGTTCTGTCACATTATAGTGAAGCCACAGATGTATAATGTGACAGAACTGACCGTCTGCCTACTGCCTCGGA	Eurofins
Elane.519	TGCTGTTGACAGTGAGCGACAGTGTGCTACAAGAGCTCAATAGTGAAGCCACAGATGTATTGAGCTCTTGTAGCACACTGGTGCCTACTGCCTCGGA	Eurofins
Elane.537	TGCTGTTGACAGTGAGCGCCAATGTGACAGTGGTGACTAATAGTGAAGCCACAGATGTATTAGTCACCACTGTCACATTGATGCCTACTGCCTCGGA	Eurofins
Elane.740	TGCTGTTGACAGTGAGCGACAGATTGGATCAATTCCATTATAGTGAAGCCACAGATGTATAATGGAATTGATCCAATCTGCTGCCTACTGCCTCGGA	Eurofins
Elane.778	TGCTGTTGACAGTGAGCGACACCTTCTTACCCATCCCAAATAGTGAAGCCACAGATGTATTTGGGATGGGTAAGAAGGTGGTGCCTACTGCCTCGGA	Eurofins

Elane.874	TGCTGTTGACAGTGAGCGCACCAGAGACCTCTCCAT<br>TTTATAGTGAAGCCACAGATGTATAAAATGGAGAGGT<br>CTCTGGTATGCCTACTGCCTCGGA	Eurofins
Alas2.219	TGCTGTTGACAGTGAGCGAAAGGACCAACCTGTTCT<br>CAAATAGTGAAGCCACAGATGTATTTGAGAACAGGTT<br>GGTCCTTGTGCCTACTGCCTCGGA	Eurofins
Alas2.467	TGCTGTTGACAGTGAGCGACAGGCTTTCGGTTATGA<br>CCAATAGTGAAGCCACAGATGTATTGGTCATAACCG<br>AAAGCCTGGTGCCTACTGCCTCGGA	Eurofins
Alas2.493	TGCTGTTGACAGTGAGCGCCAGAGACAAGATCATGG<br>AGAATAGTGAAGCCACAGATGTATTCTCCATGATCTT<br>GTCTCTGATGCCTACTGCCTCGGA	Eurofins
Alas2.531	TGCTGTTGACAGTGAGCGCACCGTGTGTTCAAGACT<br>GTGATAGTGAAGCCACAGATGTATCACAGTCTTGAA<br>CACACGGTATGCCTACTGCCTCGGA	Eurofins
Alas2.532	TGCTGTTGACAGTGAGCGCCCGTGTGTTCAAGACTG<br>TGAATAGTGAAGCCACAGATGTATTCACAGTCTTGAA<br>CACACGGTTGCCTACTGCCTCGGA	Eurofins
Fn1.2539	TGCTGTTGACAGTGAGCGAAAAGTACATTGTCAATGT<br>CTATAGTGAAGCCACAGATGTATAGACATTGACAATG<br>TACTTTCTGCCTACTGCCTCGGA	Eurofins
Fn1.2802	TGCTGTTGACAGTGAGCGACCGGTGTTCAGTACAAC<br>ATCATAGTGAAGCCACAGATGTATGATGTTGTACTGA<br>ACACCGGGTGCCTACTGCCTCGGA	Eurofins
Fn1.4143	TGCTGTTGACAGTGAGCGCCAGTAGGATACTACACA<br>GTTATAGTGAAGCCACAGATGTATAACTGTGTAGTAT<br>CCTACTGATGCCTACTGCCTCGGA	Eurofins
Fn1.8128	TGCTGTTGACAGTGAGCGCTACCTGTTTGTTATTTAT<br>CAATAGTGAAGCCACAGATGTATTGATAAATAACAAA<br>CAGGTAATGCCTACTGCCTCGGA	Eurofins
Fn1.8279	TGCTGTTGACAGTGAGCGAAAAGACAAGTGTTTTAAT<br>AAATAGTGAAGCCACAGATGTATTTATTAAAACACTT<br>GTCTTTCTGCCTACTGCCTCGGA	Eurofins
Tnfaip2.2040	TGCTGTTGACAGTGAGCGCCCGGAACATACTGGACA<br>TCAATAGTGAAGCCACAGATGTATTGATGTCCAGTAT<br>GTTCCGGATGCCTACTGCCTCGGA	Eurofins
Tnfaip2.228	TGCTGTTGACAGTGAGCGATGCGAAGAAGAAGGAGA<br>AGAATAGTGAAGCCACAGATGTATTCTTCTCCTTCTT<br>CTTCGCAGTGCCTACTGCCTCGGA	Eurofins
Tnfaip2.2489	TGCTGTTGACAGTGAGCGACAGGGATGTCATGAGAT<br>AGAATAGTGAAGCCACAGATGTATTCTATCTCATGAC<br>ATCCCTGGTGCCTACTGCCTCGGA	Eurofins
Tnfaip2.2728	TGCTGTTGACAGTGAGCGCCCCGGGTTCTCTCAGAT<br>CAAATAGTGAAGCCACAGATGTATTTGATCTGAGAGA<br>ACCCGGGATGCCTACTGCCTCGGA	Eurofins
Tnfaip2.3443	TGCTGTTGACAGTGAGCGAACCGCTTGTTGATGACA<br>TTAATAGTGAAGCCACAGATGTATTAATGTCATCAAC<br>AAGCGGTCTGCCTACTGCCTCGGA	Eurofins
Lyz2.181	TGCTGTTGACAGTGAGCGATCAGCACGAGAGCAATT<br>ATAATAGTGAAGCCACAGATGTATTATAATTGCTCTC<br>GTGCTGAGTGCCTACTGCCTCGGA	Eurofins
Lyz2.185	TGCTGTTGACAGTGAGCGACACGAGAGCAATTATAA<br>CACATAGTGAAGCCACAGATGTATGTGTTATAATTGC<br>TCTCGTGCTGCCTACTGCCTCGGA	Eurofins

Lyz2.505	TGCTGTTGACAGTGAGCGCTTCGGTCTCTTTCTCACT GTATAGTGAAGCCACAGATGTATACAGTGAGAAAGA GACCGAATTGCCTACTGCCTCGGA	Eurofins
Lyz2.790	TGCTGTTGACAGTGAGCGAACCTGTGTGAATAAAAA TACATAGTGAAGCCACAGATGTATGTATTTTTATTCA CACAGGTCTGCCTACTGCCTCGGA	Eurofins
Lyz2.937	TGCTGTTGACAGTGAGCGAAGGAGTCTCAGTGGATG AGAATAGTGAAGCCACAGATGTATTCTCATCCACTGA GACTCCTGTGCCTACTGCCTCGGA	Eurofins
Beta-s.479	TGCTGTTGACAGTGAGCGATGGCTCACAAGTACCAC TAAATAGTGAAGCCACAGATGTATTTAGTGGTACTTG TGAGCCAGTGCCTACTGCCTCGGA	Eurofins
Beta-s.525	TGCTGTTGACAGTGAGCGCACAATGGTTAATTGTTC CCAATAGTGAAGCCACAGATGTATTGGGAACAATTAA CCATTGTTTGCCTACTGCCTCGGA	Eurofins
Beta-s.557	TGCTGTTGACAGTGAGCGATCAGTTGTTGGCAAAAT GATATAGTGAAGCCACAGATGTATATCATTTTGCCAA CAACTGACTGCCTACTGCCTCGGA	Eurofins
Beta-s.558	TGCTGTTGACAGTGAGCGCCAGTTGTTGGCAAAATG ATAATAGTGAAGCCACAGATGTATTATCATTTTGCCA ACAACTGATGCCTACTGCCTCGGA	Eurofins
Beta-s.565	TGCTGTTGACAGTGAGCGCTGGCAAAATGATAAAGA CATTTAGTGAAGCCACAGATGTAAATGTCTTTATCAT TTTGCCAATGCCTACTGCCTCGGA	Eurofins
Hbb-b1.18	TGCTGTTGACAGTGAGCGCTTCTGTTGTGTTGACTT GCAATAGTGAAGCCACAGATGTATTGCAAGTCAACA CAACAGAATTGCCTACTGCCTCGGA	Eurofins
Hbb-b1.37	TGCTGTTGACAGTGAGCGAAACCTCAGAAACAGACA TCATTAGTGAAGCCACAGATGTAATGATGTCTGTTTC TGAGGTTGTGCCTACTGCCTCGGA	Eurofins
Hbb-b1.527	TGCTGTTGACAGTGAGCGAAAAGGTTATATGTCCCC TAGATAGTGAAGCCACAGATGTATCTAGGGGACATA TAACCTTTGTGCCTACTGCCTCGGA	Eurofins
Hbb-b1.539	TGCTGTTGACAGTGAGCGATCCCCTAGAGAAAAACT GTCATAGTGAAGCCACAGATGTATGACAGTTTTTCTC TAGGGGACTGCCTACTGCCTCGGA	Eurofins
Hbb-b1.541	TGCTGTTGACAGTGAGCGACCCTAGAGAAAAACTGT CAATTAGTGAAGCCACAGATGTAATTGACAGTTTTTC TCTAGGGGTGCCTACTGCCTCGGA	Eurofins
Hbb-b2.13	TGCTGTTGACAGTGAGCGATTGCAACTTCAGAAACA GACATAGTGAAGCCACAGATGTATGTCTGTTTCTGAA GTTGCAAGTGCCTACTGCCTCGGA	Eurofins
Hbb-b2.233	TGCTGTTGACAGTGAGCGCAAAGGTGATAACTGCCT TTAATAGTGAAGCCACAGATGTATTAAAGGCAGTTAT CACCTTTTTGCCTACTGCCTCGGA	Eurofins
Hbb-b2.3	TGCTGTTGACAGTGAGCGATTGTGTTGACTTGCAAC TTCATAGTGAAGCCACAGATGTATGAAGTTGCAAGTC AACACAACTGCCTACTGCCTCGGA	Eurofins
Hbb-b2.524	TGCTGTTGACAGTGAGCGATAGAGAAAAACTGTCAA GTGTTAGTGAAGCCACAGATGTAACACTTGACAGTTT TTCTCTAGTGCCTACTGCCTCGGA	Eurofins
Hbb-b2.590	TGCTGTTGACAGTGAGCGCATGATATTTACTGTCATC TCATAGTGAAGCCACAGATGTATGAGATGACAGTAA ATATCATTTGCCTACTGCCTCGGA	Eurofins

Spire1.1316	TGCTGTTGACAGTGAGCGCAAGCTGTTCGAGGTCAGAAAATAGTGAAGCCACAGATGTATTTTCTGACCTCGAACAGCTTTTGCCTACTGCCTCGGA	Eurofins
Spire1.1556	TGCTGTTGACAGTGAGCGCAAGGAAGACACAGTCTTTCTATAGTGAAGCCACAGATGTATAGAAAGACTGTGTCTTCCTTTTGCCTACTGCCTCGGA	Eurofins
Spire1.1979	TGCTGTTGACAGTGAGCGCTAGCACAGTTGTAAGCAGTAATAGTGAAGCCACAGATGTATTACTGCTTACAACTGTGCTAATGCCTACTGCCTCGGA	Eurofins
Spire1.3129	TGCTGTTGACAGTGAGCGATCCAGCTGTTTTTAAGACTAATAGTGAAGCCACAGATGTATTAGTCTTAAAAACAGCTGGACTGCCTACTGCCTCGGA	Eurofins
Spire1.3570	TGCTGTTGACAGTGAGCGACCAAGTGTGTTAAGAGTTCAATAGTGAAGCCACAGATGTATTGAACTCTTAACACACTTGGCTGCCTACTGCCTCGGA	Eurofins
Epb4.2.2149	TGCTGTTGACAGTGAGCGCAAGGCTCACTGTGGAAGTAGATAGTGAAGCCACAGATGTATCTACTTCCACAGTGAGCCTTTTGCCTACTGCCTCGGA	Eurofins
Epb4.2.2582	TGCTGTTGACAGTGAGCGCAACACTATTTGTCTTAGACTATAGTGAAGCCACAGATGTATAGTCTAAGACAAATAGTGTTTTGCCTACTGCCTCGGA	Eurofins
Epb4.2.2713	TGCTGTTGACAGTGAGCGCCAGATGTTTCATACCATTTTATAGTGAAGCCACAGATGTATAAAATGGTATGAAACATCTGATGCCTACTGCCTCGGA	Eurofins
Epb4.2.2761	TGCTGTTGACAGTGAGCGCCAGGAATGAAAGACAACCTAATAGTGAAGCCACAGATGTATTAGGTTGTCTTTCATTCCTGTTGCCTACTGCCTCGGA	Eurofins
Epb4.2.2908	TGCTGTTGACAGTGAGCGCTAGAGCTATTATAACACATTATAGTGAAGCCACAGATGTATAATGTGTTATAATAGCTCTATTGCCTACTGCCTCGGA	Eurofins
Igj.1401	TGCTGTTGACAGTGAGCGCCAGTTTGTAAGTATGATACAATAGTGAAGCCACAGATGTATTGTATCATACTTACAAACTGTTGCCTACTGCCTCGGA	Eurofins
Igj.1561	TGCTGTTGACAGTGAGCGAGAGCTTGTTTATCATGTTCAATAGTGAAGCCACAGATGTATTGAACATGATAAACAAGCTCCTGCCTACTGCCTCGGA	Eurofins
Igj.1739	TGCTGTTGACAGTGAGCGAAAGTAGAAAGATCTCACTTAATAGTGAAGCCACAGATGTATTAAGTGAGATCTTTCTACTTGTGCCTACTGCCTCGGA	Eurofins
Igj.198	TGCTGTTGACAGTGAGCGAACCATTCTTGCTGACAACAAATAGTGAAGCCACAGATGTATTTGTTGTCAGCAAGAATGGTCTGCCTACTGCCTCGGA	Eurofins
Igj.653	TGCTGTTGACAGTGAGCGACAGAAGTTATACTCACTGCTATAGTGAAGCCACAGATGTATAGCAGTGAGTATAACTTCTGGTGCCTACTGCCTCGGA	Eurofins
Fam132a.1093	TGCTGTTGACAGTGAGCGATGGACAGTATGTCTCTGTGTTTAGTGAAGCCACAGATGTAAACACAGAGACATACTGTCCAGTGCCTACTGCCTCGGA	Eurofins
Fam132a.1238	TGCTGTTGACAGTGAGCGCTAGAGGAGCTGAAAAGGACTATAGTGAAGCCACAGATGTATAGTCCTTTTCAGCTCCTCTAATGCCTACTGCCTCGGA	Eurofins
Fam132a.30	TGCTGTTGACAGTGAGCGATAGGGAGAAAGTAGTTCTCTTTAGTGAAGCCACAGATGTAAAGAGAACTACTTTCTCCCTAGTGCCTACTGCCTCGGA	Eurofins

Fam132a.31	TGCTGTTGACAGTGAGCGCAGGGAGAAAGTAGTTCTCTTTTAGTGAAGCCACAGATGTAAAAGAGAACTACTTTCTCCCTATGCCTACTGCCTCGGA	Eurofins
Fam132a.632	TGCTGTTGACAGTGAGCGCCTGCAGGAATTTCAGGAGATATAGTGAAGCCACAGATGTATATCTCCTGAAATTCCTGCAGTTGCCTACTGCCTCGGA	Eurofins
Cd200r1.1362	TGCTGTTGACAGTGAGCGCAAGCACAAATGTTCTAATGAATAGTGAAGCCACAGATGTATTCATTAGAACATTTGTGCTTTTGCCTACTGCCTCGGA	Eurofins
Cd200r1.1363	TGCTGTTGACAGTGAGCGCAGCACAAATGTTCTAATGAAATAGTGAAGCCACAGATGTATTTCATTAGAACATTTGTGCTTTGCCTACTGCCTCGGA	Eurofins
Cd200r1.337	TGCTGTTGACAGTGAGCGATGGGTCAAGTTGTACTGATAATAGTGAAGCCACAGATGTATTATCAGTACAACTTGACCCAGTGCCTACTGCCTCGGA	Eurofins
Cd200r1.526	TGCTGTTGACAGTGAGCGCAGCATACAAAGTAGATACAAATAGTGAAGCCACAGATGTATTTGTATCTACTTTGTATGCTATGCCTACTGCCTCGGA	Eurofins
Cd200r1.537	TGCTGTTGACAGTGAGCGATAGATACAAAGACCAATGAAATAGTGAAGCCACAGATGTATTTCATTGGTCTTTGTATCTACTGCCTACTGCCTCGGA	Eurofins
Camp.133	TGCTGTTGACAGTGAGCGATCCGAGCTGTGGATGACTTCATAGTGAAGCCACAGATGTATGAAGTCATCCACAGCTCGGAGTGCCTACTGCCTCGGA	Eurofins
Camp.134	TGCTGTTGACAGTGAGCGCCCGAGCTGTGGATGACTTCAATAGTGAAGCCACAGATGTATTGAAGTCATCCACAGCTCGGATGCCTACTGCCTCGGA	Eurofins
Camp.250	TGCTGTTGACAGTGAGCGCTCCGAGTGAAGGAGACTGTATTAGTGAAGCCACAGATGTAATACAGTCTCCTTCACTCGGAATGCCTACTGCCTCGGA	Eurofins
Camp.405	TGCTGTTGACAGTGAGCGCCAGCCCTTTCGGTTCAAGAAATAGTGAAGCCACAGATGTATTTCTTGAACCGAAAGGGCTGTTGCCTACTGCCTCGGA	Eurofins
Camp.406	TGCTGTTGACAGTGAGCGAAGCCCTTTCGGTTCAAGAAAATAGTGAAGCCACAGATGTATTTTCTTGAACCGAAAGGGCTGTGCCTACTGCCTCGGA	Eurofins
Pbk.1255	TGCTGTTGACAGTGAGCGCTAACTAGATATATGTAGTTAATAGTGAAGCCACAGATGTATTAACTACATATATCTAGTTAATGCCTACTGCCTCGGA	Eurofins
Pbk.1292	TGCTGTTGACAGTGAGCGATAGATTCTAGAAGTAGCTTTATAGTGAAGCCACAGATGTATAAAGCTACTTCTAGAATCTAGTGCCTACTGCCTCGGA	Eurofins
Pbk.1420	TGCTGTTGACAGTGAGCGATACGTACATGTGGTACAGATATAGTGAAGCCACAGATGTATATCTGTACCACATGTACGTAGTGCCTACTGCCTCGGA	Eurofins
Pbk.231	TGCTGTTGACAGTGAGCGCAACGTGATTGCTACAATGGAATAGTGAAGCCACAGATGTATTCCATTGTAGCAATCACGTTATGCCTACTGCCTCGGA	Eurofins
Pbk.726	TGCTGTTGACAGTGAGCGAAAGCTGCTTCATGGAGACATATAGTGAAGCCACAGATGTATATGTCTCCATGAAGCAGCTTCTGCCTACTGCCTCGGA	Eurofins

Supplementary Figures

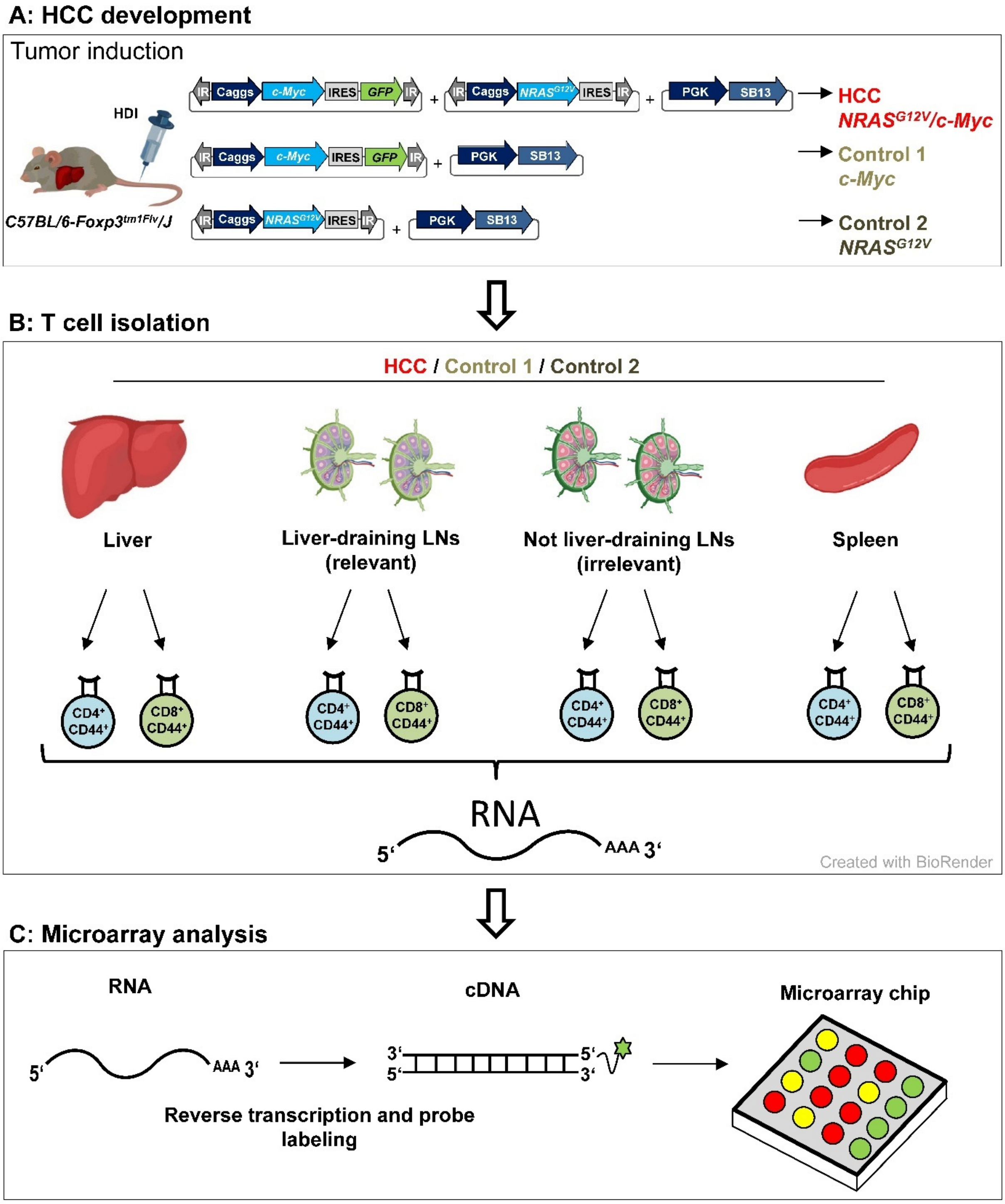

Fig. S1. Experimental setup for microarray analysis. A, HCC tumor induction in C57BL/6-Foxp3^{tm1Flv}/J mice via HDI-mediated delivery of two oncogenes (*NRAS*^{G12}*/c-Myc*) or respective controls (control 1: *c-Myc*, control 2: *NRAS*^{G12V}). **B,** RNA isolation from $CD4^+$ $CD44^+$ T cells and $CD8^+$ $CD44^+$ T cells sorted from liver, liver-draining LNs, not liver-draining LNs, and spleen of

HCC-bearing and both tumor-free control mice. **C,** Sample preparation for microarray analysis comprising reverse transcription, probe labeling, and hybridization on a microarray chip. HCC – hepatocellular carcinoma, HDI – hydrodynamic tail vein injection, LN – lymph nodes.

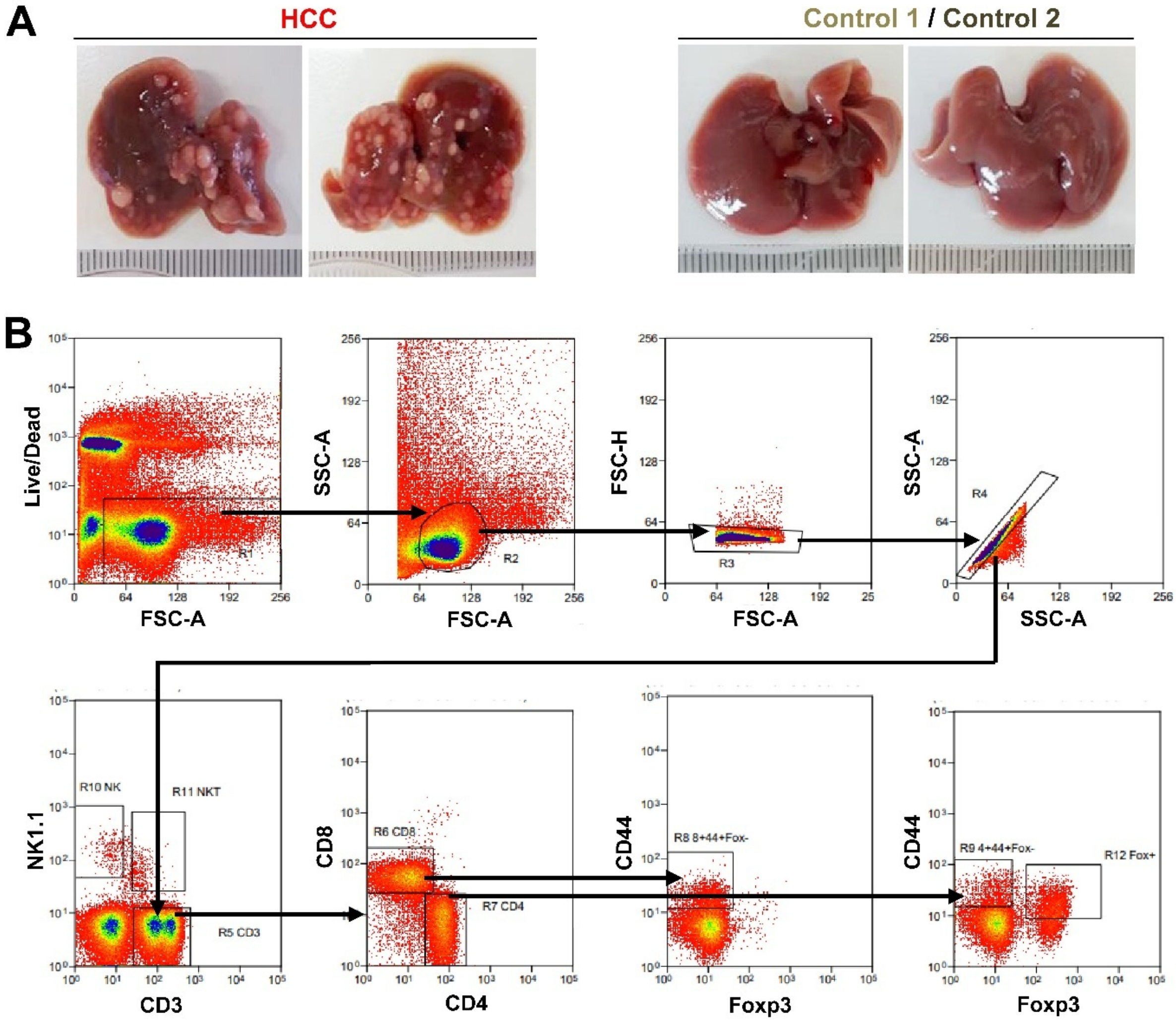

Fig. S2. Sample isolation for microarray analysis. A, Depicted are explanted livers of HCC-bearing mice (C57BL/6-Foxp3^{tm1Flv}/J) and HCC-free controls (Control 1, C1/Control 2, C2) 5-8 weeks after HDI. **B,** Gating strategy to define CD3$^+$ NK1.1$^-$ CD4$^+$ Foxp3$^-$ CD44$^+$ T cells and CD3$^+$ NK1.1$^-$ CD8$^+$ Foxp3$^-$ CD44$^+$ T cells in HCC-bearing and HCC-free animals. HCC – hepatocellular carcinoma, HDI – hydrodynamic tail vein injection.

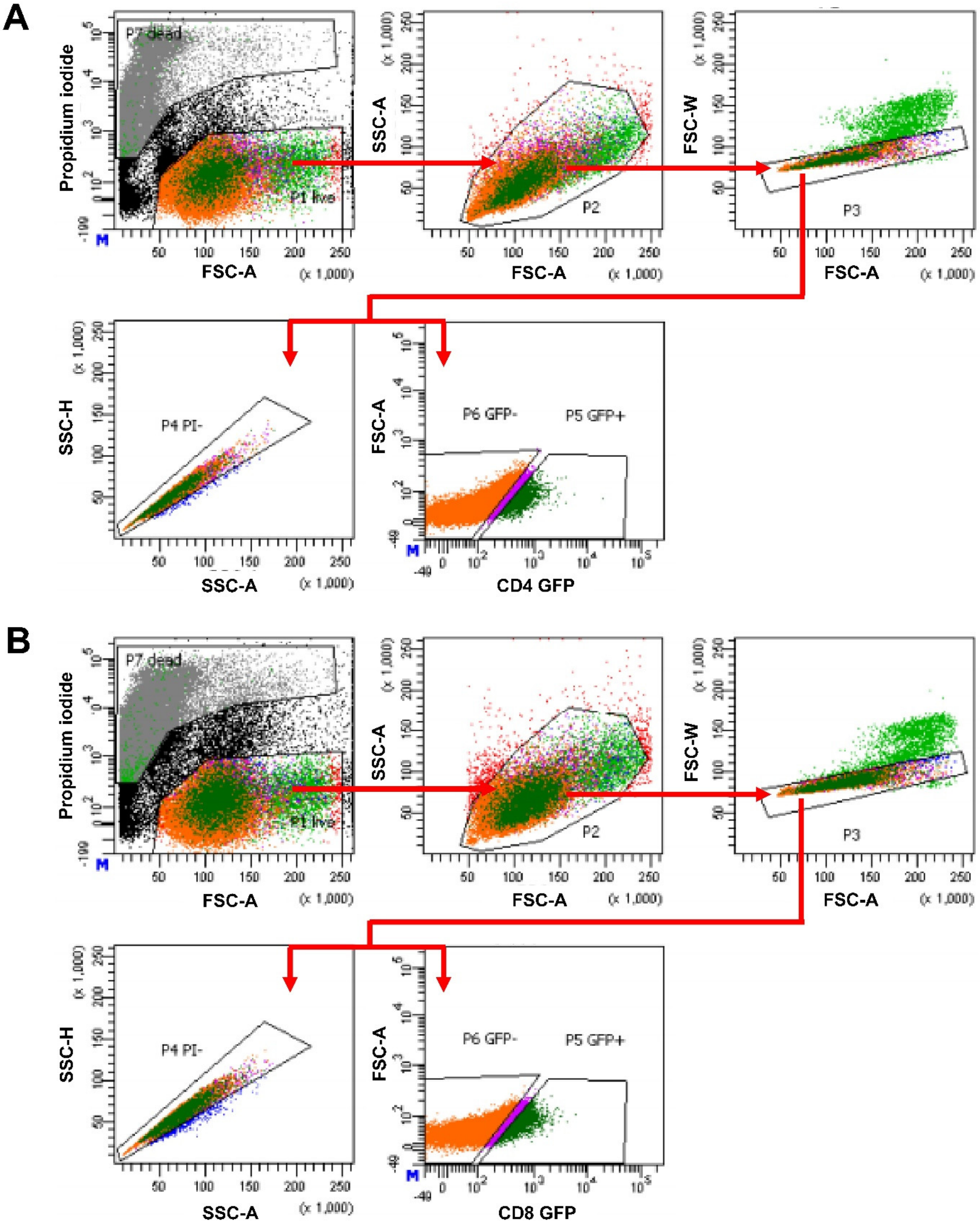

Fig. S3. Gating strategy for CD4 and CD8 T cells sorted after transduction and prior to the adoptive transfer. Transduced **(A)** CD4 T cells and **(B)** CD8 T cells were sorted after three days of *in vitro* stimulation for GFP^+ expression according to the depicted gating strategy. GFP – green fluorescent protein.

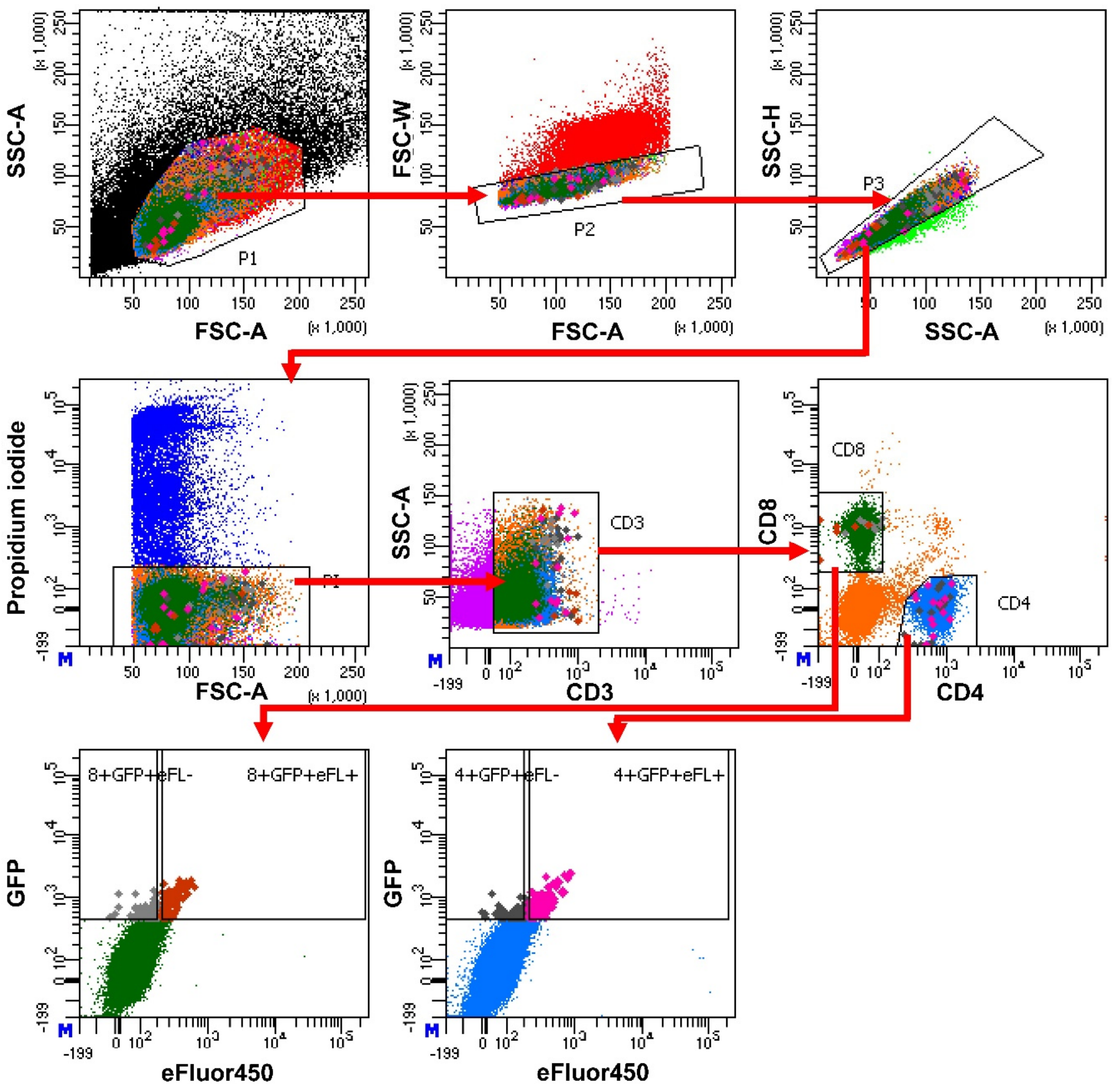

Fig. S4. Gating strategy for CD4 and CD8 T cells after adoptive transfer prior to the screen. Isolated CD4 and CD8 T cells were sorted five days post-transfer from liver, spleen, relevant LNs, irrelevant LNs, and blood of recipient mice according to the depicted gating strategy. LNs – lymph nodes, GFP – green fluorescent protein.

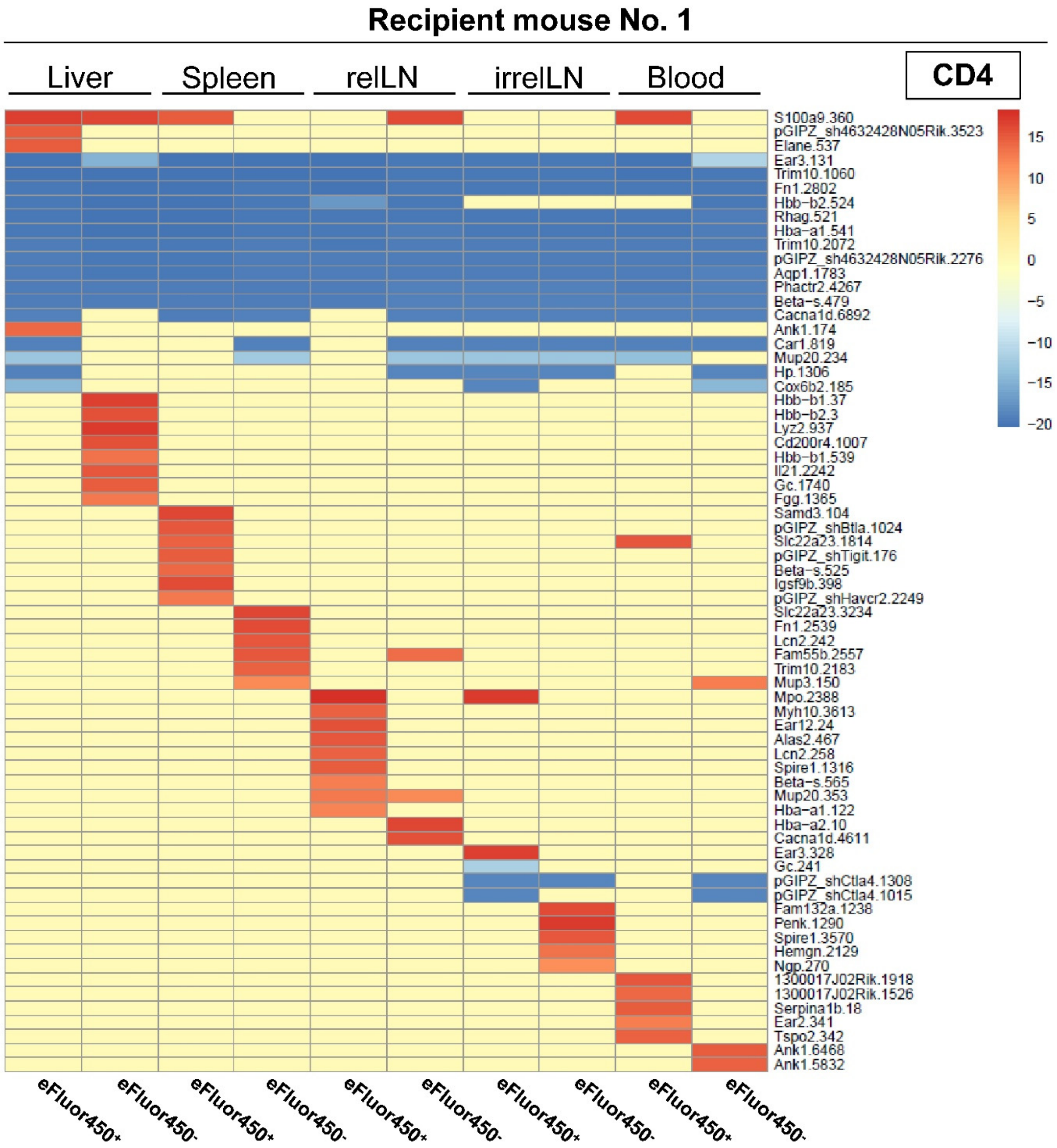

Fig. S5. *In vivo* RNAi screen identified enriched shRNAs in CD4 T cells during HCC development. Screen analysis on eFluor450⁻ and eFluor450⁺ CD4⁺ T cells, which were isolated five days after adoptive transfer from the liver, spleen, relLN, irrelLN, and blood (out-probes) of HCC-bearing mice ($NRAS^{G12V}$/*c-Myc* genotype). Transferred CD4⁺ T cells (out-probes) were compared to CD4 T cells before the adoptive transfer (in-probes). ShRNA enrichments (log2-fold changes) are shown in a heatmap with upregulated (>0, shown in red) and downregulated shRNAs (<0, shown in blue). Data are shown for the recipient mouse No. 1. relLN - relevant lymph nodes, irrelLN - irrelevant lymph nodes.

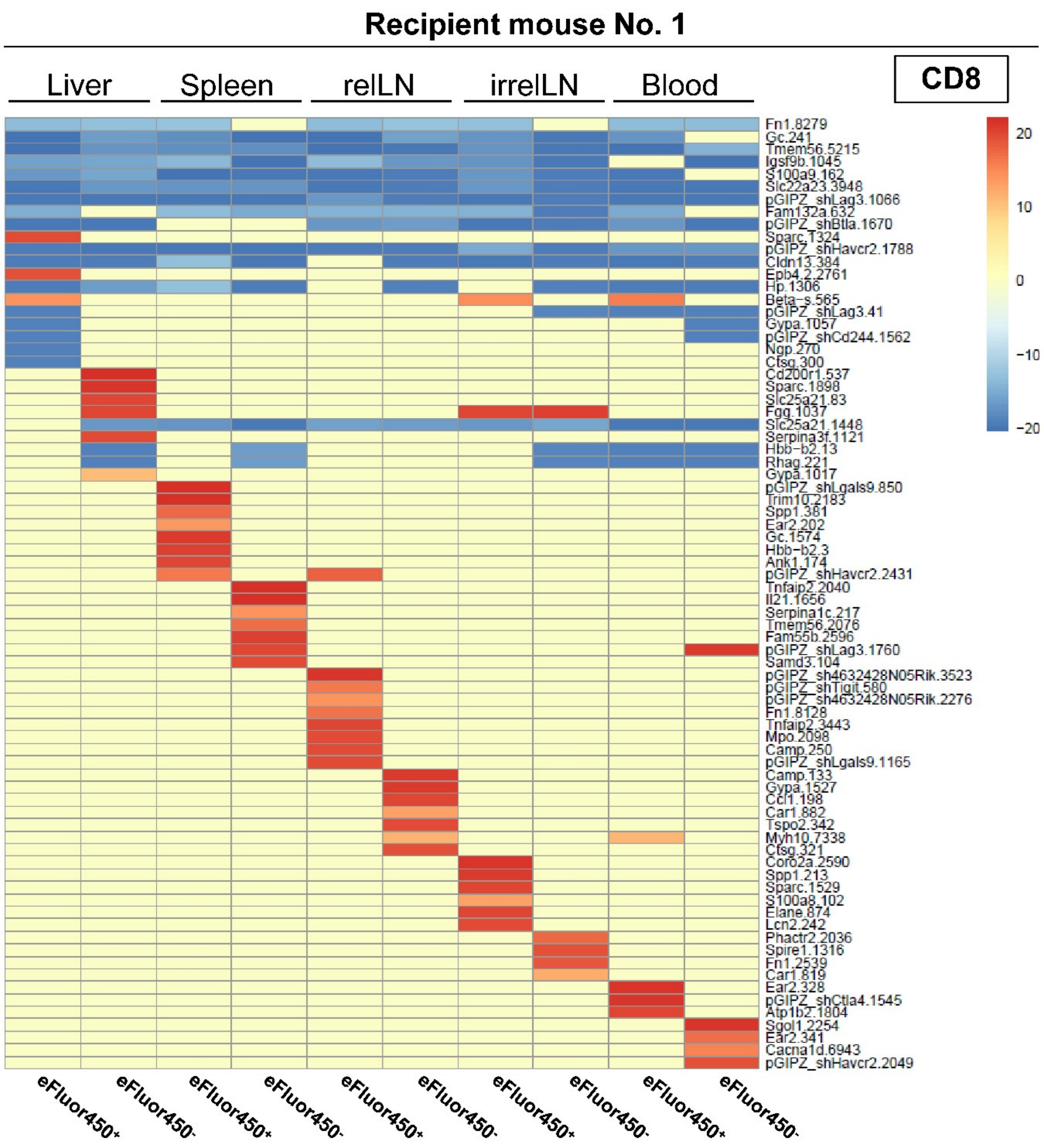

Fig. S6. *In vivo* RNAi screen identified enriched shRNAs in CD8 T cells during HCC development. Screen analysis on eFluor450⁻ and eFluor450⁺ CD8⁺ T cells, which were isolated five days after the adoptive transfer from the liver, spleen, relLN, irrelLN, and blood (out-probes) of HCC-bearing mice (*NRAS*^{G12V}*/c-Myc* genotype). Transferred CD8⁺ T cells (out-probes) were compared to CD8⁺ T cells before the adoptive transfer (in-probes). ShRNA enrichments (log2-fold changes) are shown in a heatmap with upregulated (>0, marked in red) and downregulated shRNAs (<0, marked in blue). Data are shown for the recipient mouse No. 1. relLN - relevant lymph nodes, irrelLN - irrelevant lymph nodes.

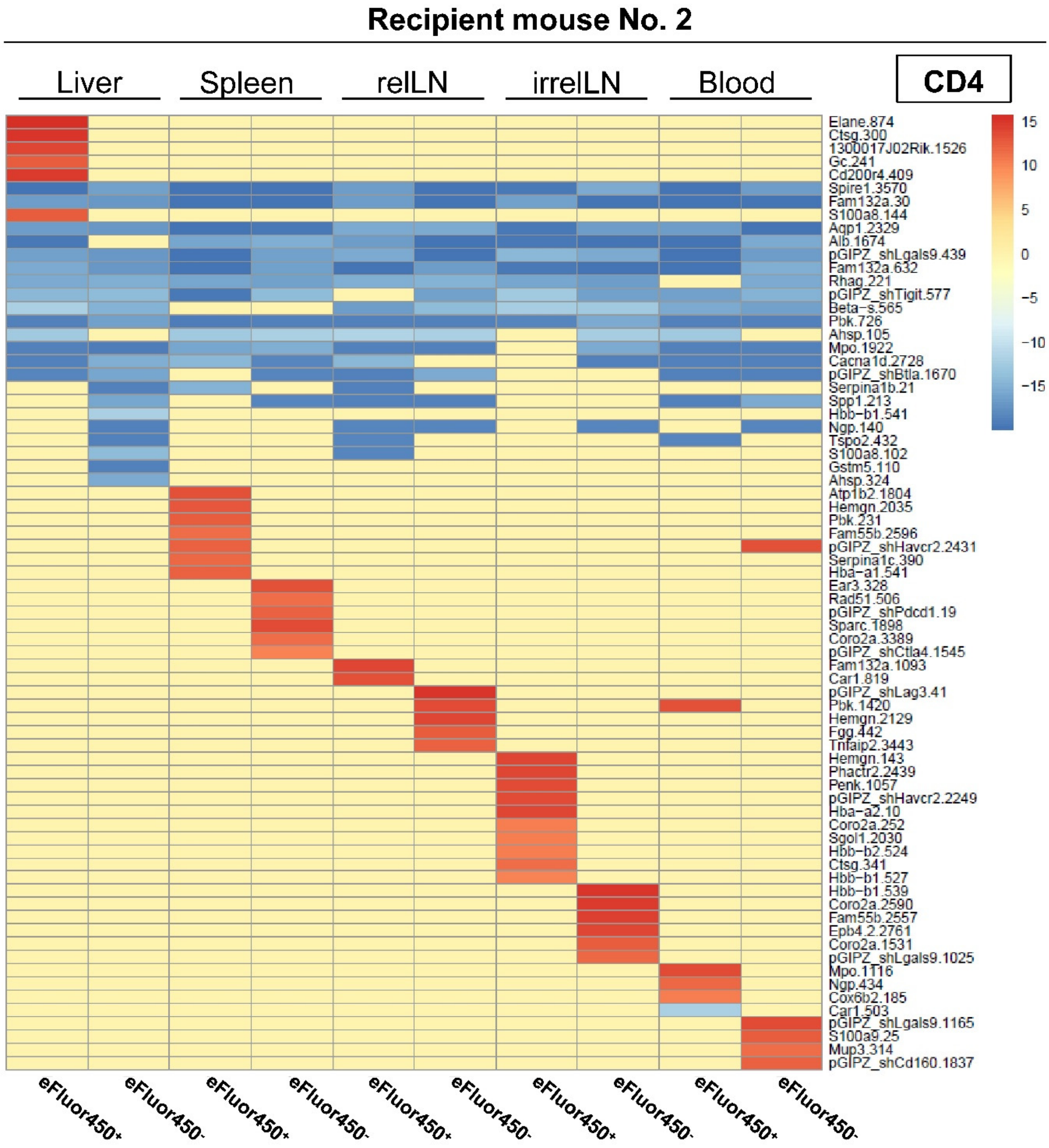

Fig. S7. *In vivo* RNAi screen identified enriched shRNAs in CD4 T cells during HCC development. Screen analysis on eFluor450$^-$ and eFluor450$^+$ CD4$^+$ T cells, which were isolated five days after the adoptive transfer from the liver, spleen, relLN, irrelLN, and blood (out-probes) of HCC-bearing mice (*NRAS^{G12V}/c-Myc* genotype). Transferred CD4$^+$ T cells (out-probes) were compared to CD4 T cells before the adoptive transfer (in-probes). ShRNA enrichments (log2-fold changes) are shown in a heatmap with upregulated (>0, marked in red) and downregulated shRNAs (<0, marked in blue). Data are shown for the recipient mouse No. 2. relLN - relevant lymph nodes, irrelLN - irrelevant lymph nodes.

Recipient mouse No. 2

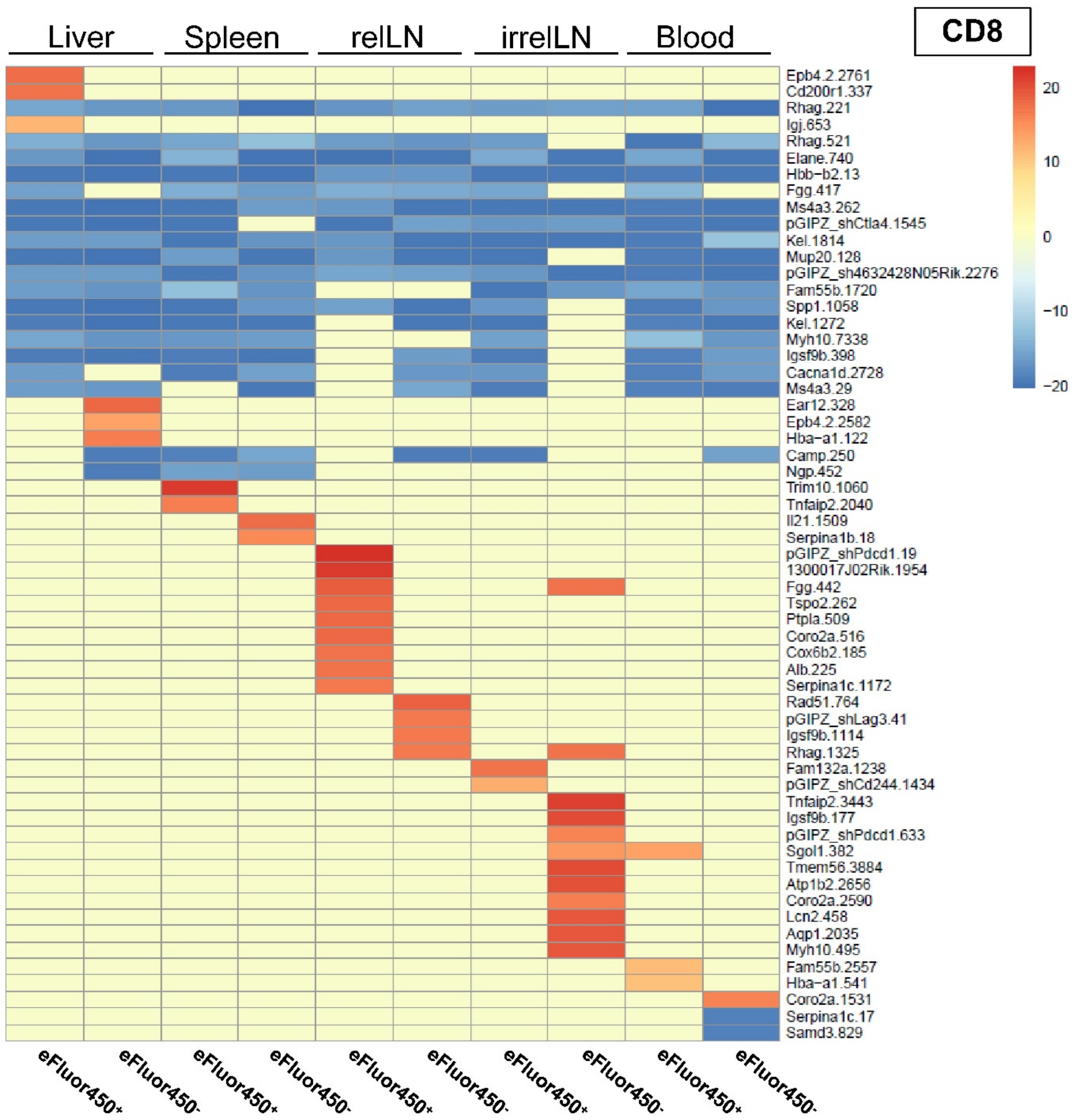

Fig. S8. *In vivo* RNAi screen identified enriched shRNAs in CD8 T cells during HCC development. Screen analysis on eFluor450⁻ and eFluor450⁺ CD8⁺ T cells, which were isolated five days after the adoptive transfer from the liver, spleen, relLN, irrelLN, and blood (out-probes) of HCC-bearing mice (*NRAS*^{G12V}*/c-Myc* genotype). Transferred CD8⁺ T cells (out-probes) were compared to CD8 T cells before the adoptive transfer (in-probes). ShRNA enrichments (log2-fold changes) are shown in a heatmap with upregulated (>0, marked in red) and downregulated shRNAs (<0, marked in blue). Data are shown for the recipient mouse No. 2. relLN - relevant lymph nodes, irrelLN - irrelevant lymph nodes.

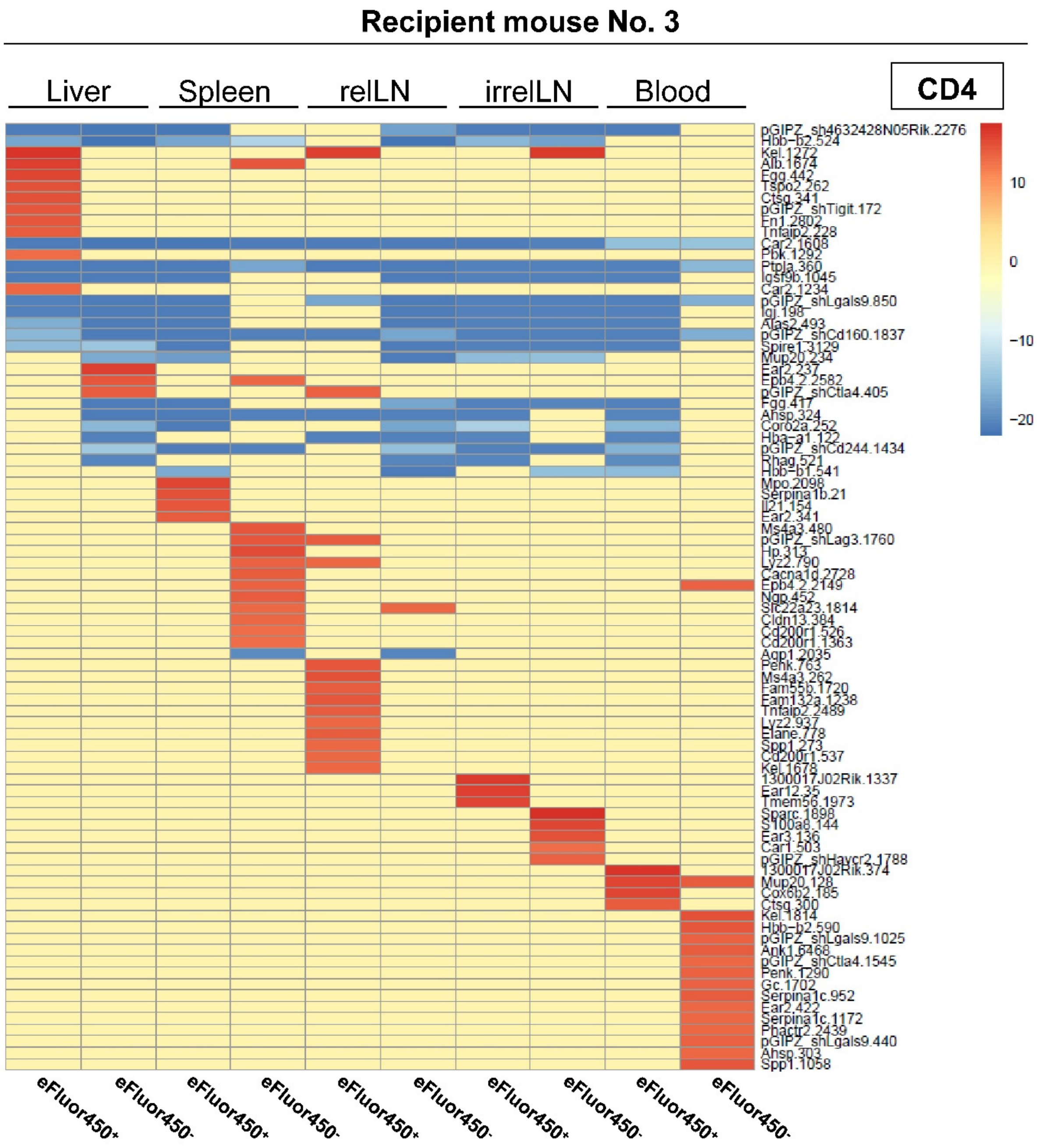

Fig. S9. *In vivo* RNAi screen identified enriched shRNAs in CD4 T cells during HCC development. Screen analysis on eFluor450$^-$ and eFluor450$^+$ CD4$^+$ T cells, which were isolated five days after the adoptive transfer from the liver, spleen, relLN, irrelLN, and blood (out-probes) of HCC-bearing mice (*NRAS^{G12V}/c-Myc* genotype). Transferred CD4$^+$ T cells (out-probes) were compared to CD4 T cells before the adoptive transfer (in-probes). ShRNA enrichments (log2-fold changes) are shown in a heatmap with upregulated (>0, marked in red) and downregulated shRNAs (<0, marked in blue). Data are shown for the recipient mouse No. 3. relLN - relevant lymph nodes, irrelLN - irrelevant lymph nodes.

Recipient mouse No. 3

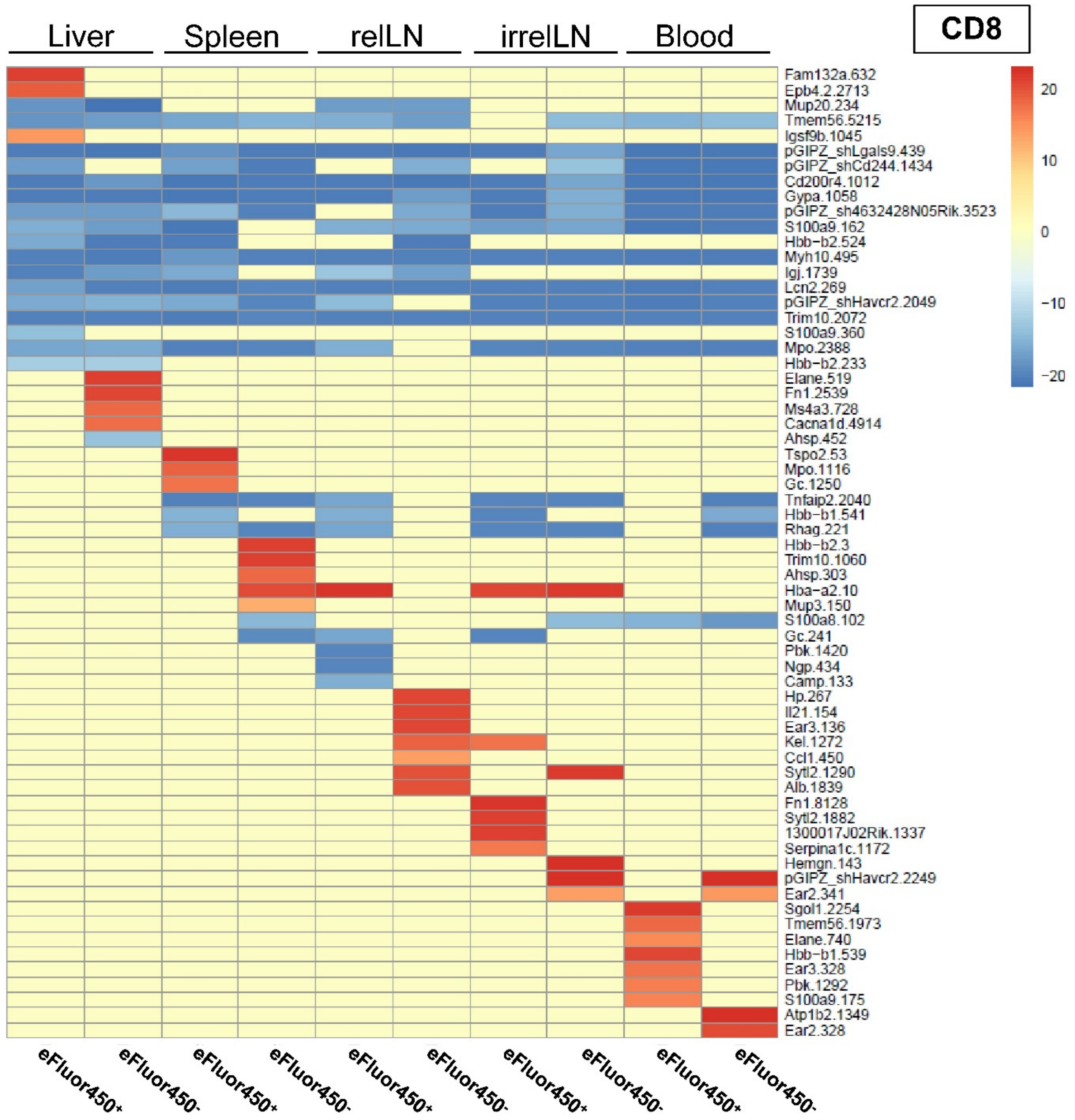

Fig. S10. *In vivo* RNAi screen identified enriched shRNAs in CD8 T cells during HCC development. Screen analysis on eFluor450$^-$ and eFluor450$^+$ CD8$^+$ T cells, which were isolated five days after the adoptive transfer from the liver, spleen, relLN, irrelLN, and blood (out-probes) of HCC-bearing mice (*NRAS^{G12V}/c-Myc* genotype). Transferred CD8$^+$ T cells (out-probes) were compared to CD8 T cells before the adoptive transfer (in-probes). ShRNA enrichments (log2-fold changes) are shown in a heatmap with upregulated (>0, marked in red) and downregulated shRNAs (<0, marked in blue). Data are shown for the recipient mouse No. 3. relLN - relevant lymph nodes, irrelLN - irrelevant lymph nodes.

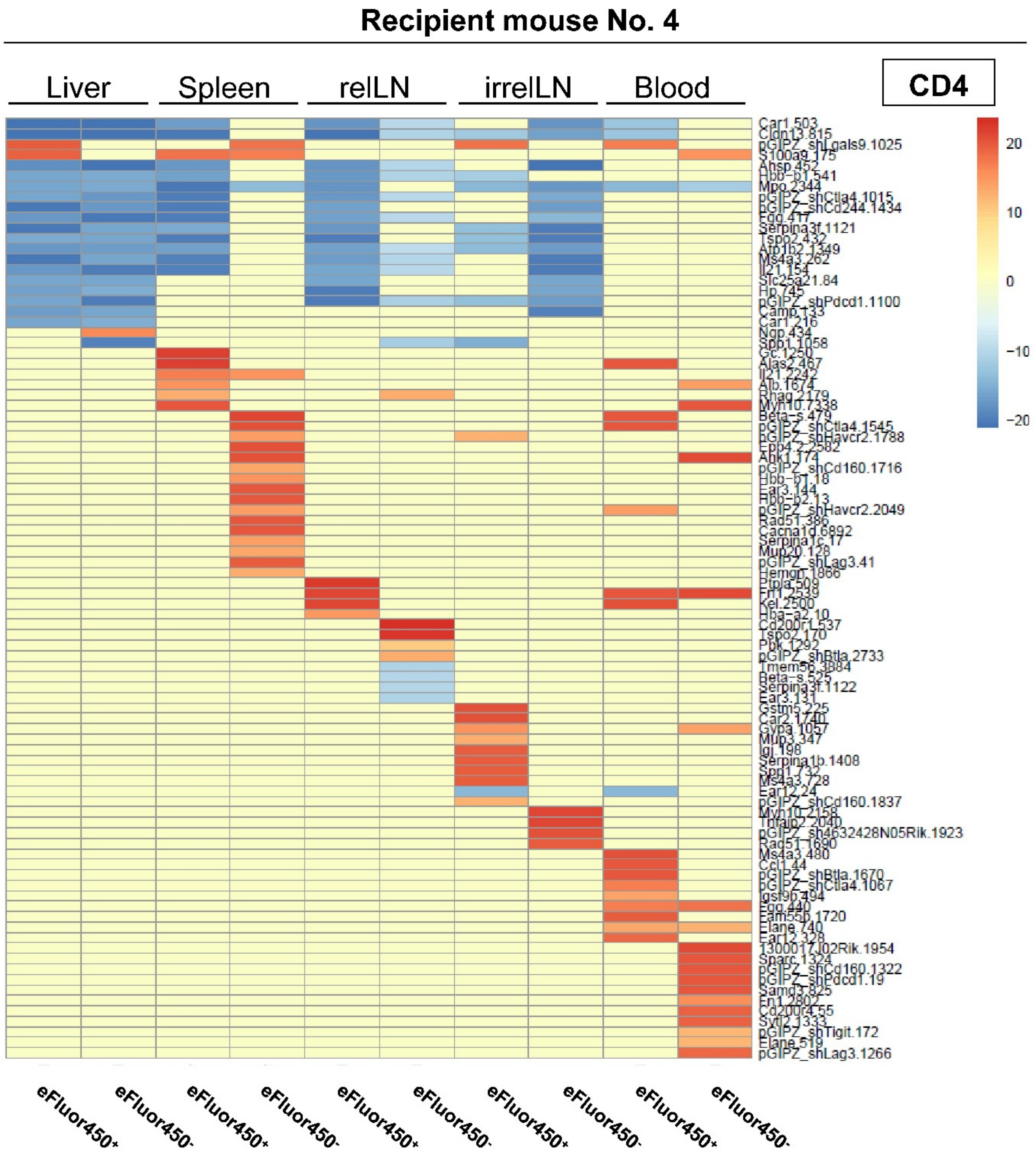

Fig. S11. *In vivo* RNAi screen identified enriched shRNAs in CD4 T cells during HCC development. Screen analysis on eFluor450$^-$ and eFluor450$^+$ CD4$^+$ T cells, which were isolated five days after the adoptive transfer from the liver, spleen, relLN, irrelLN, and blood (out-probes) of HCC-bearing mice (*NRAS^{G12V}/c-Myc* genotype). Transferred CD4$^+$ T cells (out-probes) were compared to CD4 T cells before the adoptive transfer (in-probes). ShRNA enrichments (log2-fold changes) are shown in a heatmap with upregulated (>0, marked in red) and downregulated shRNAs (<0, marked in blue). Data are shown for the recipient mouse No. 4. relLN - relevant lymph nodes, irrelLN - irrelevant lymph nodes.

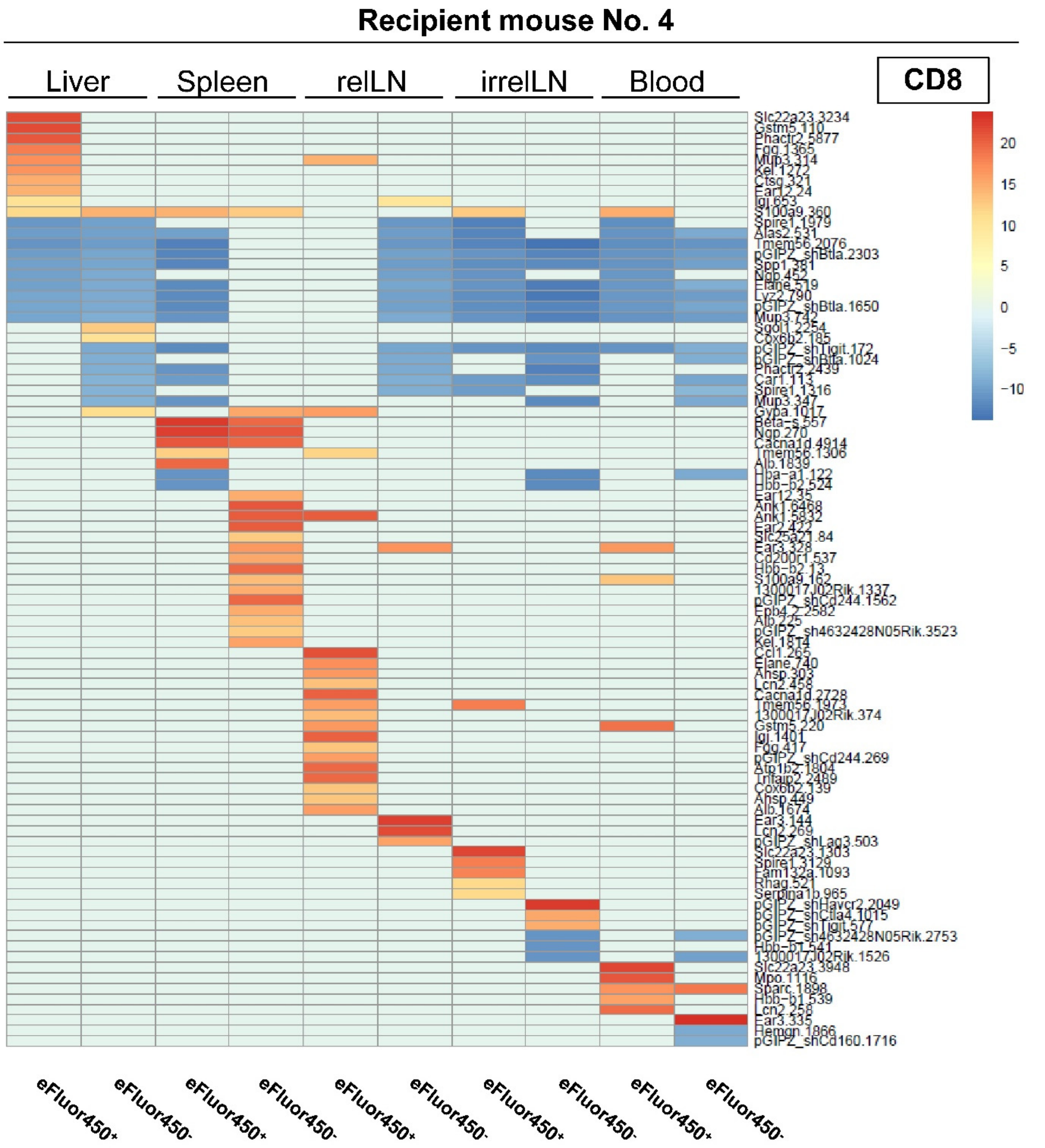

Fig. S12. *In vivo* RNAi screen identified enriched shRNAs in CD8 T cells during HCC development. Screen analysis on eFluor450$^-$ and eFluor450$^+$ CD8$^+$ T cells, which were isolated five days after the adoptive transfer from the liver, spleen, relLN, irrelLN, and blood (out-probes) of HCC-bearing mice (*NRAS^{G12V}/c-Myc* genotype). Transferred CD8$^+$ T cells (out-probes) were compared to CD8 T cells before the adoptive transfer (in-probes). ShRNA enrichments (log2-fold changes) are shown in a heatmap with upregulated (>0, marked in red) and downregulated shRNAs (<0, marked in blue). Data are shown for the recipient mouse No. 4. relLN - relevant lymph nodes, irrelLN - irrelevant lymph nodes.

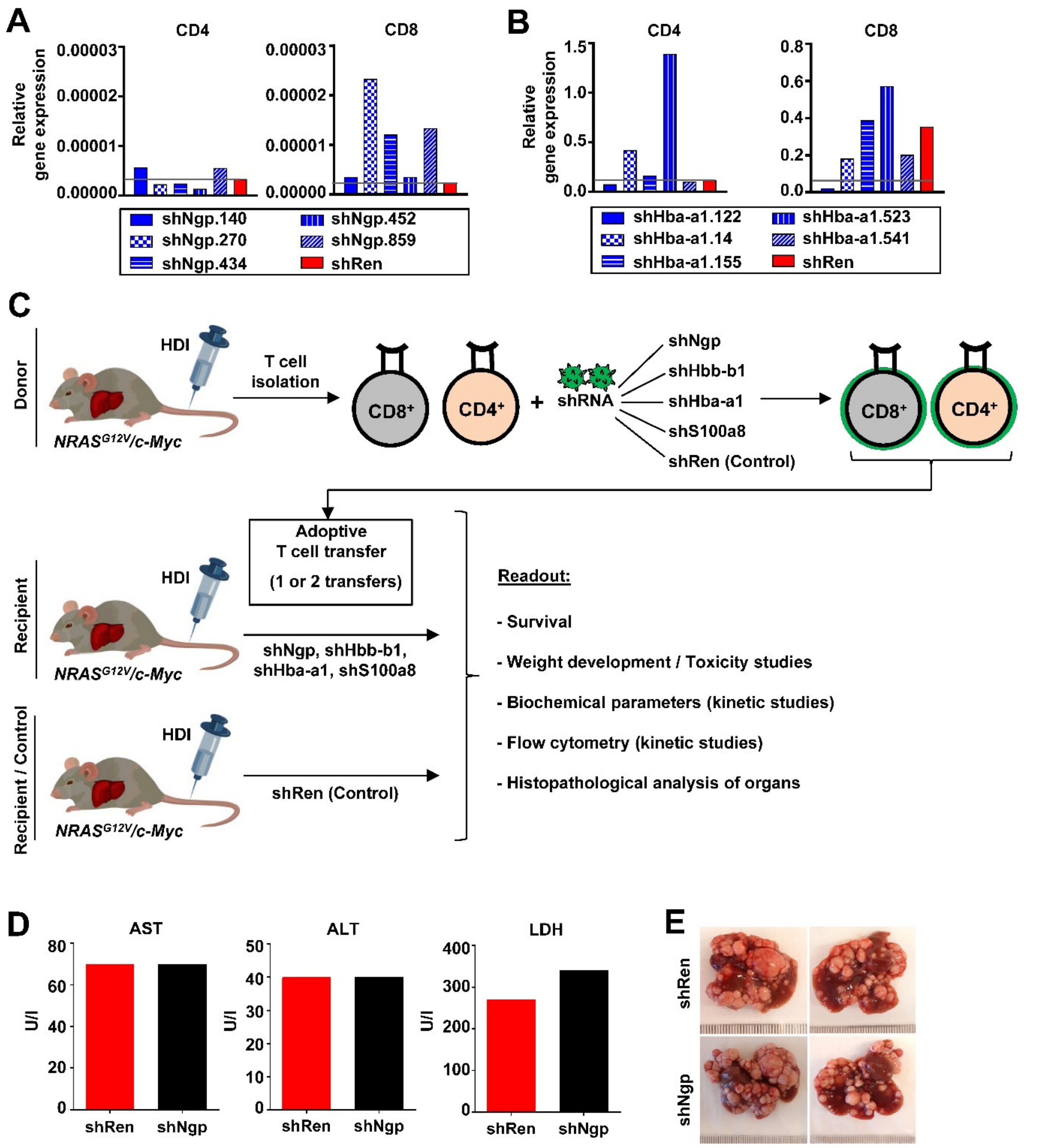

Fig. S13. qPCR analysis identified the most efficacious shRNAs which were selected for the validation studies *in vivo*. A-B, qPCR analysis of five shRNAs targeting **(A)** *Ngp* and **(B)** *Hba-a1* was performed to assess the knockdown efficiency in CD4 and CD8 T cells *in vitro*. Data are shown from one representative experiment. **C,** Experimental setup for the *in vivo* validation studies. CD4 and CD8 T cells were isolated from HCC-bearing donor mice (*NRAS*^{G12V}/*c-Myc* genotype) and transduced with lentiviral shRNA targeting *Ngp, Hbb-b1, Hba-a1*, *S100a8*, or control *Ren* (pGIPZ-GFP). Successfully transduced GFP^{+} CD4^{+} and GFP^{+} CD8^{+} T cells were sorted, labeled with proliferation dye eFluor450 and adoptively transferred into HCC-bearing recipient mice. During the entire survival study, we monitored weight

development, biochemical parameters in plasma over time (kinetic studies), and expression of several classical ICIs molecules and activation markers on innate and adaptive immune cells in recipient mice via flow cytometry. At the end of the experiment, histopathological examination of organs was performed to exclude cytotoxic side effects upon T cell therapy. **D,** Baseline AST, ALT, and LDH levels in plasma of recipient mice before the adoptive cell transfer. Data are shown from one representative mouse per group. **E,** Representative images of explanted livers from recipient mice treated with shRen (control) and shNgp.140 upon HCC development on sampling day. HCC – hepatocellular carcinoma, shRNA – short hairpin RNA, ALT – alanine aminotransferase, AST – aspartate aminotransferase, LDH – lactate dehydrogenase, GFP – green fluorescent protein, *Ngp* – neutrophil granule protein, *Hbb* – hemoglobin subunit beta*, Hba* – hemoglobin subunit alpha, *S100a8* – S100 calcium-binding protein A8, *Ren* – renilla.

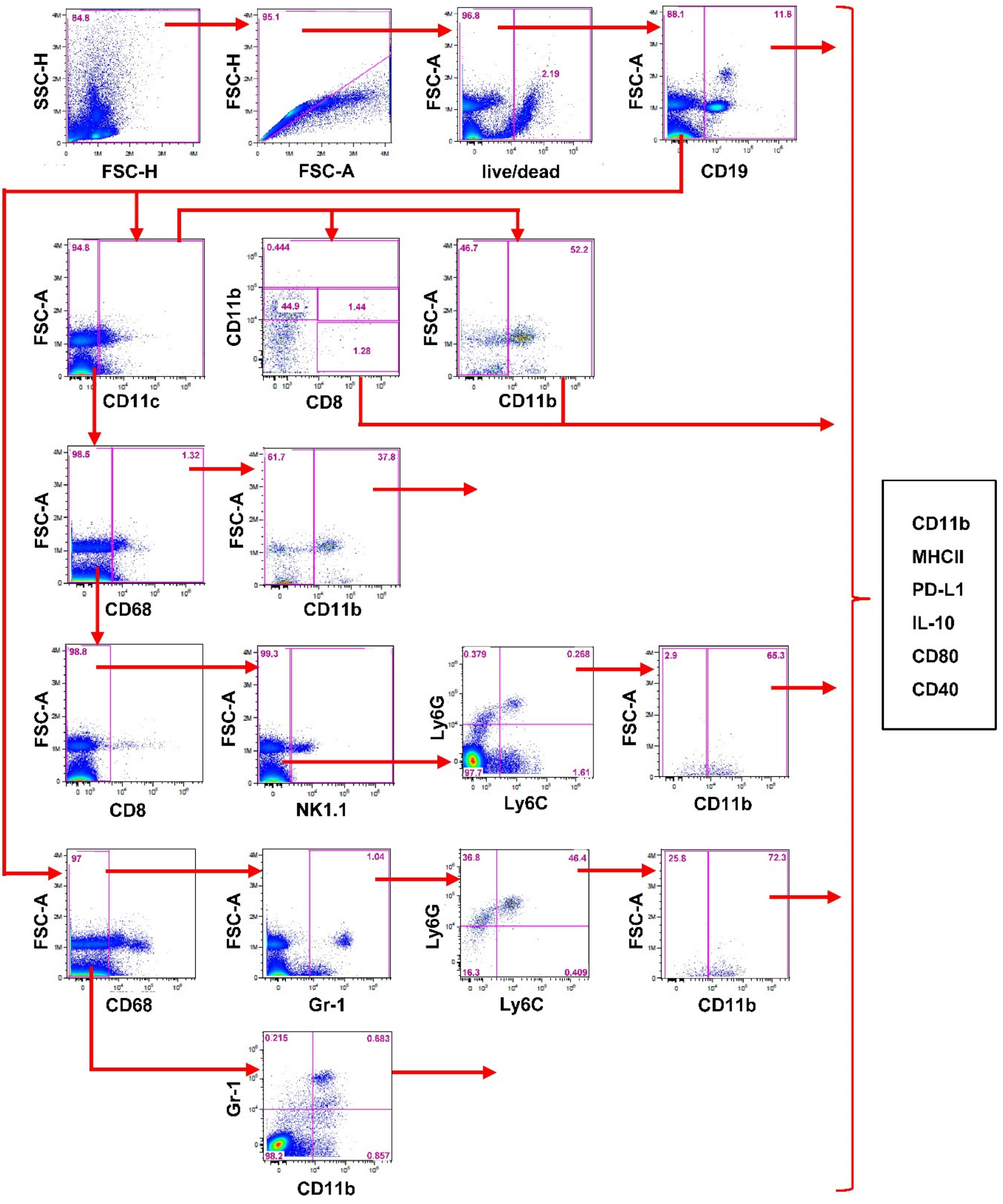

Fig. S14. Gating strategy to define B cells and innate immune cell populations in recipient mice upon adoptive T cell transfer. Single-cell suspensions were obtained from the blood of recipient mice and stained with respective antibodies for indicated surface and intracellular markers. Samples were analyzed using multicolored FACS analysis. Immune cell populations were defined according to the specific markers, as depicted in the figure.

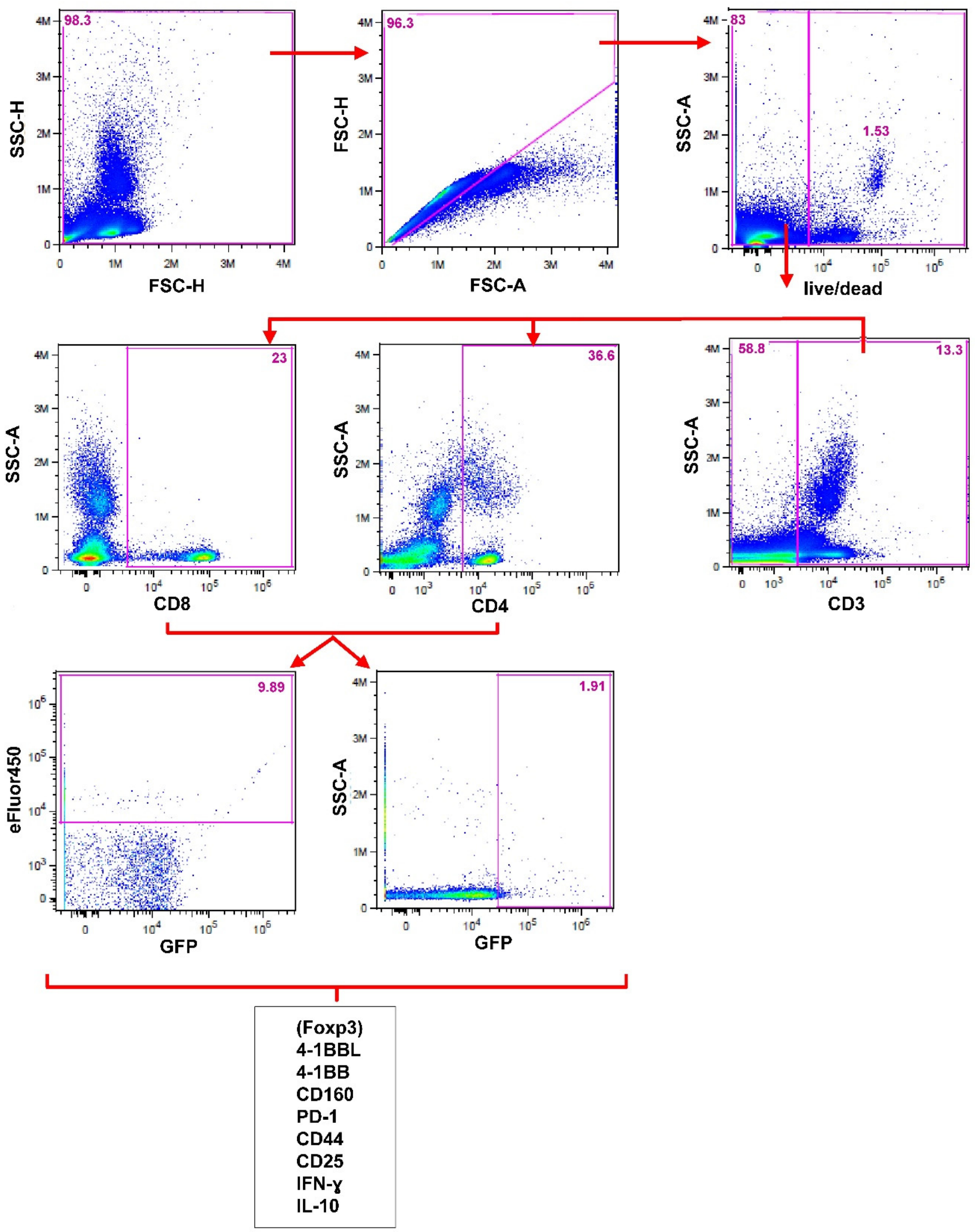

Fig. S15. Gating strategy to define adaptive immune cell populations in recipient mice upon adoptive T cell transfer. Single-cell suspensions were obtained from the blood of recipient mice and stained accordingly for the indicated surface and intracellular markers. Samples were analyzed using multicolored FACS analysis. The adoptively transferred (exogenous) T cells were identified based on their GFP expression, distinguishing them from the endogenous T cell populations. Furthermore, ICIs receptors were analyzed on both endogenous and exogenous CD4 and CD8 T cells, as depicted in the figure.

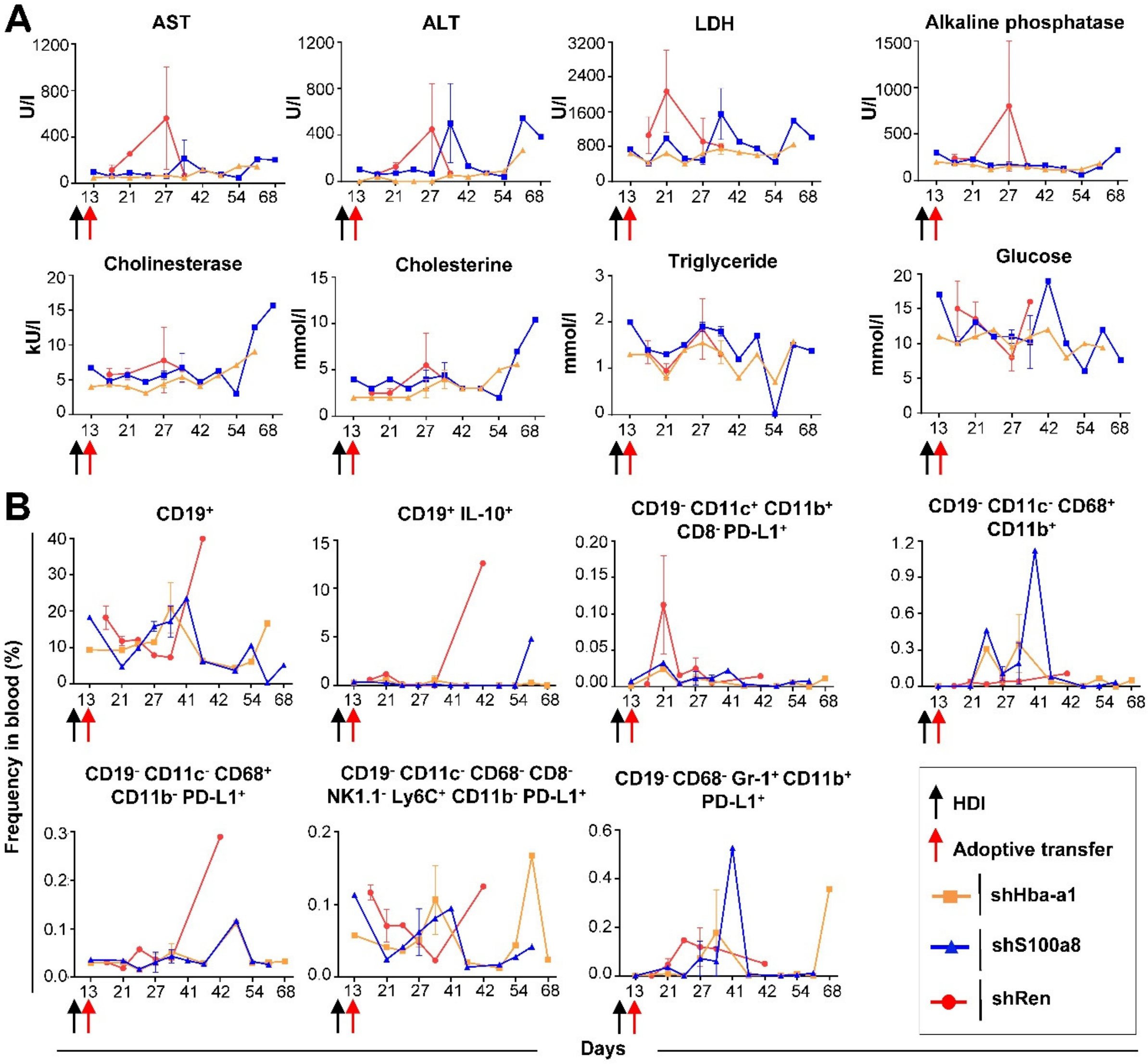

Fig. S16. *In vivo* knockdown of *Hba-a1* and *S100a8* on T cells kept under control liver biochemical parameters and PD-L1-expressing immune cell populations. A, Biochemical parameters in plasma of recipient mice following the adoptive transfer of shHba-a1, shS100a8, or control shRen-transduced T cells. **B,** Flow cytometry analysis of innate immune cells and B lymphocytes in the blood of shHba-a, shS100a8*,* and shRen recipient mice. Data represent two independent experiments, shown are the mean ± SEM, n=2. SEM - standard error of the mean.

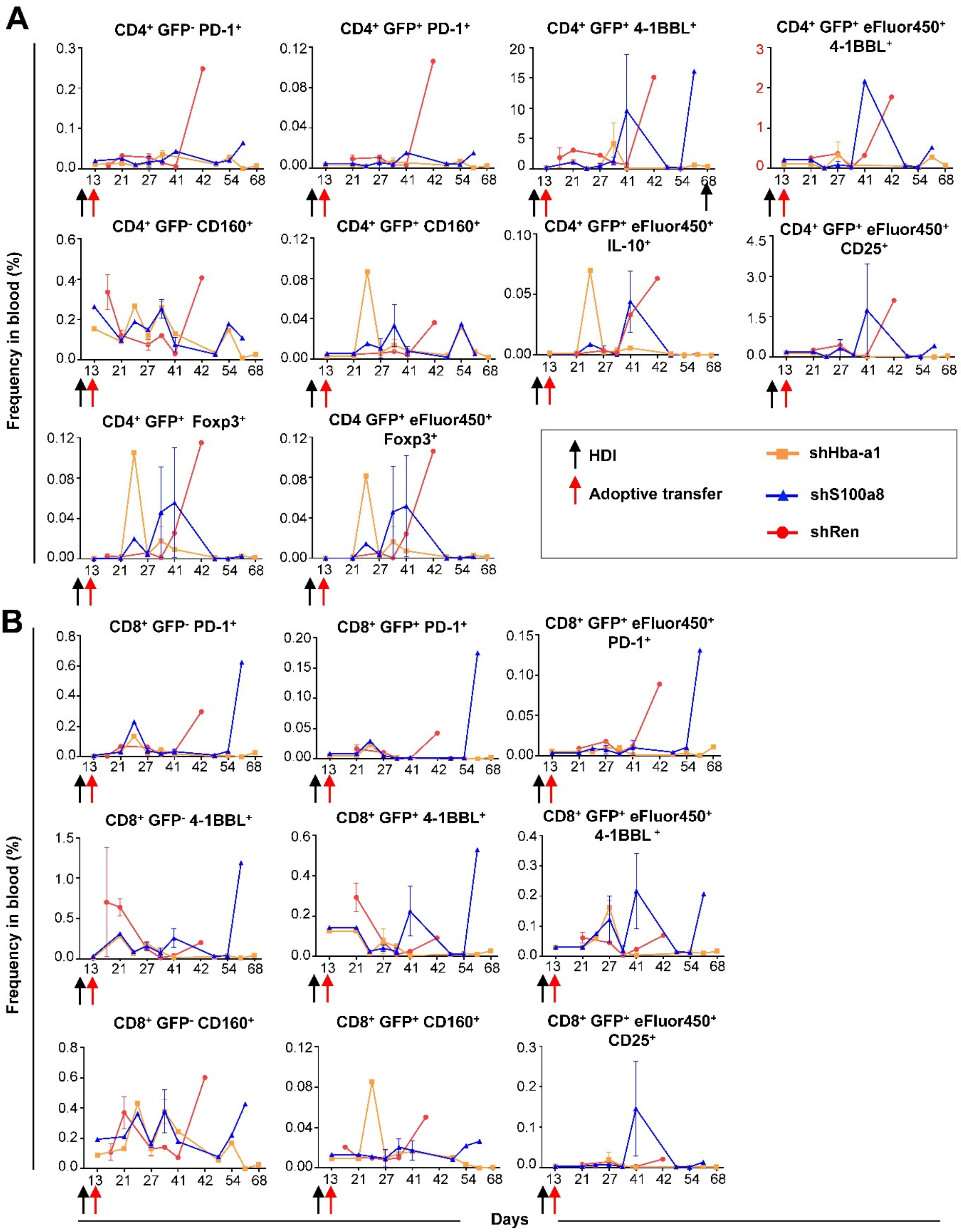

Fig. S17. *In vivo* knockdown of *Hba-a1* and *S100a8* regulated several ICI markers on T cells. A-B, Flow cytometry kinetic analysis of endogenous and exogenous T cells in the blood of shHba-a, shS100a8, and shRen recipient mice. The expression of activation and inhibitory markers was monitored over time on **(A)** CD4 and **(B)** CD8 T cells upon adoptive transfer. Data represent two independent experiments, shown are the mean ± SEM, n=2. SEM - standard error of the mean.

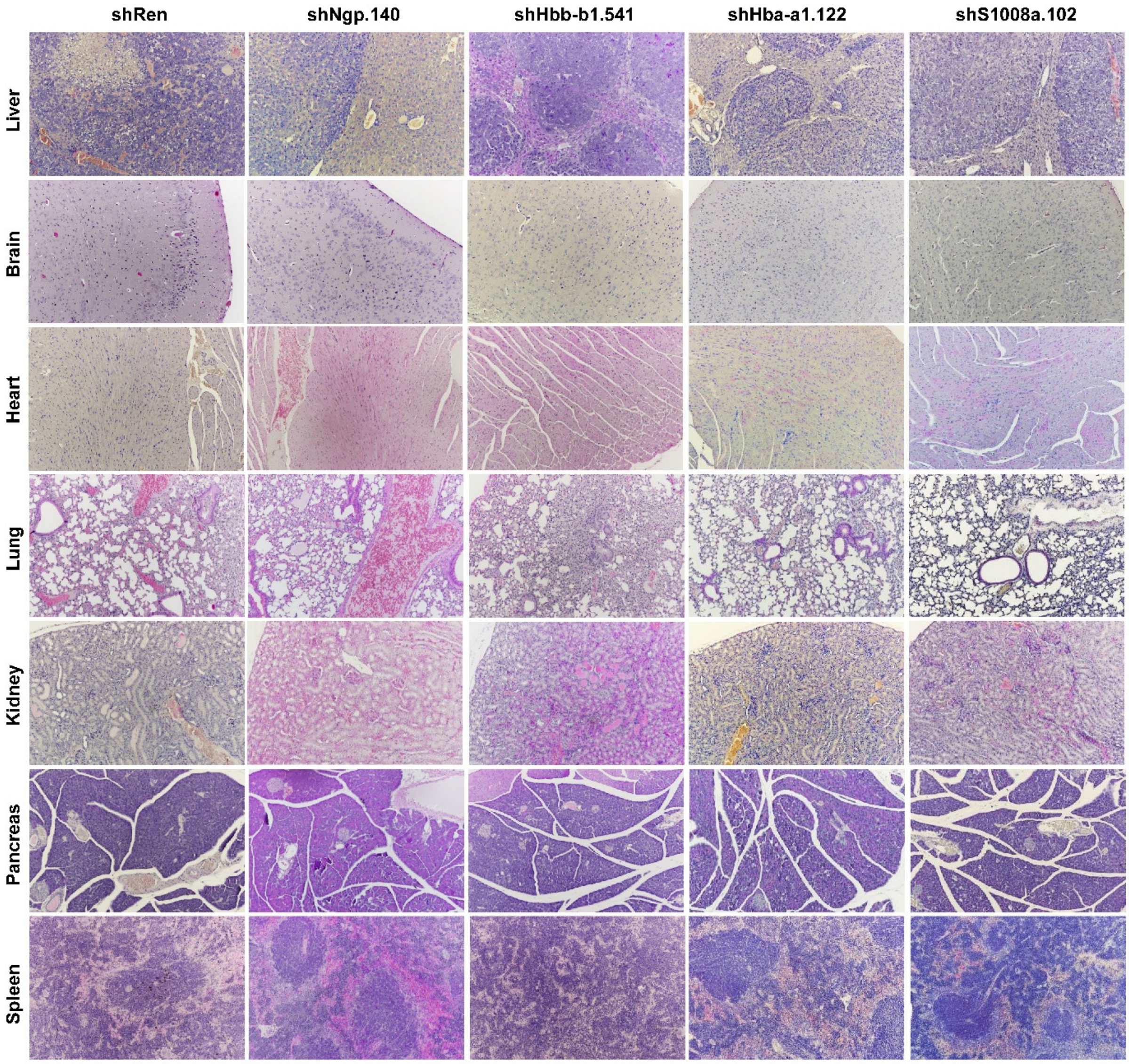

Fig. S18. T cell therapy is safe and did not induce any significant histopathological changes in organs of recipient mice. Shown are representative H&E histological sections of major organs (brain, heart, lung, kidney, pancreas, spleen) isolated from HCC-bearing recipient mice that received adoptive T cell transfer (*Ngp*, *Hbb-b1, Hba-a1,* and *S100a8* or *Ren* knockdown). Magnification ×100.

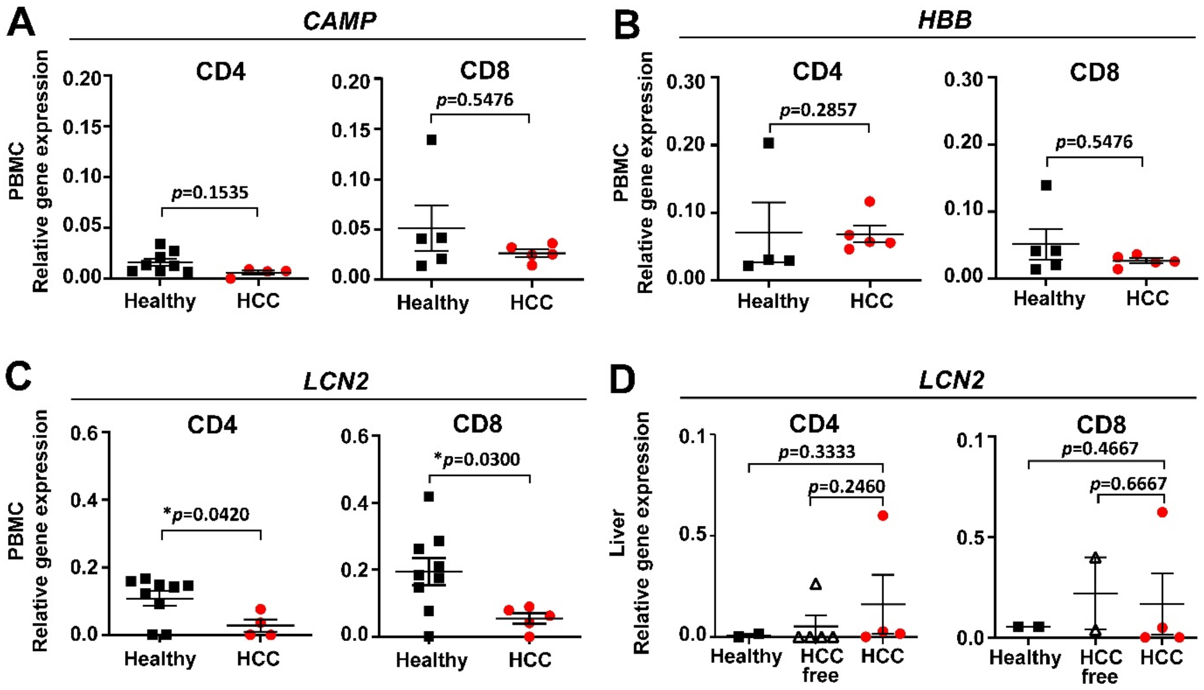

Fig. S19. Detection of new targets on CD4 and CD8 T cells in HCC patients and healthy donors using qPCR. A-D, qPCR analysis of target gene expression in CD4 T cells and CD8 T cells isolated from PBMCs and liver tissues of HCC patients and healthy donors. The expression levels of **(A)** *CAMP,* **(B)** *HBB*, and **(C-D)** *LCN2* were assessed. The data were analyzed using the Mann–Whitney nonparametric test. The data are shown as the mean ± SEM, n=2-9. HCC – hepatocellular carcinoma, PBMCs – peripheral blood mononuclear cells, *CAMP* – cathelicidin, *HBB* – hemoglobin subunit beta, *LCN2* – lipocalin 2, SEM - standard error of the mean.

Supplementary References

1. Stolk J, Aggarwal N, Hochnadel I, Wrenger S, Martinez-Delgado B, Welte T, et al. Blood Monocyte Profiles in Copd Patients with Pimm and Pizz Alpha1-Antitrypsin. *Respir Med* (2019) 148:60-2. Epub 2019/03/05. doi: 10.1016/j.rmed.2019.02.001.
2. Kang TW, Yevsa T, Woller N, Hoenicke L, Wuestefeld T, Dauch D, et al. Senescence Surveillance of Pre-Malignant Hepatocytes Limits Liver Cancer Development. *Nature* (2011) 479(7374):547-51. doi: 10.1038/nature10599.
3. Eggert T, Wolter K, Ji J, Ma C, Yevsa T, Klotz S, et al. Distinct Functions of Senescence-Associated Immune Responses in Liver Tumor Surveillance and Tumor Progression. *Cancer cell* (2016) 30(4):533-47. doi: 10.1016/j.ccell.2016.09.003.

4. Petriv N, Neubert L, Vatashchuk M, Timrott K, Suo H, Hochnadel I, et al. Increase of Alpha-Dicarbonyls in Liver and Receptor for Advanced Glycation End Products on Immune Cells Are Linked to Nonalcoholic Fatty Liver Disease and Liver Cancer. *Oncoimmunology* (2021) 10(1):1874159. doi: 10.1080/2162402X.2021.1874159.
5. Petriv N, Suo H, Hochnadel I, Timrott K, Bondarenko N, Neubert L, et al. Essential Roles of B-Cell Subsets in the Progression of MASLD and HCC. *JHEP Reports* (2024) 11(15). doi: 10.1016/j.jhepr.2024.101189.
6. Dauch D, Rudalska R, Cossa G, Nault JC, Kang TW, Wuestefeld T, et al. A Myc-Aurora Kinase a Protein Complex Represents an Actionable Drug Target in P53-Altered Liver Cancer. *Nature medicine* (2016) 22(7):744-53. doi: 10.1038/nm.4107.
7. Carlson CM, Frandsen JL, Kirchhof N, McIvor RS, Largaespada DA. Somatic Integration of an Oncogene-Harboring Sleeping Beauty Transposon Models Liver Tumor Development in the Mouse. *Proceedings of the National Academy of Sciences of the United States of America* (2005) 102(47):17059-64. doi: 10.1073/pnas.0502974102.
8. Hochnadel I, Hoenicke L, Petriv N, Neubert L, Reinhard E, Hirsch T, et al. Safety and Efficacy of Prophylactic and Therapeutic Vaccine Based on Live-Attenuated Listeria Monocytogenes in Hepatobiliary Cancers. *Oncogene* (2022). doi: 10.1038/s41388-022-02222-z.
9. Seehawer M, Heinzmann F, D'Artista L, Harbig J, Roux PF, Hoenicke L, et al. Necroptosis Microenvironment Directs Lineage Commitment in Liver Cancer. *Nature* (2018) 562(7725):69-75. doi: 10.1038/s41586-018-0519-y.
10. Wan YY, Flavell RA. Identifying Foxp3-Expressing Suppressor T Cells with a Bicistronic Reporter. *Proc Natl Acad Sci U S A* (2005) 102(14):5126-31. doi: 10.1073/pnas.0501701102.
11. Smyth GK. Linear Models and Empirical Bayes Methods for Assessing Differential Expression in Microarray Experiments. *Stat Appl Genet Mol Biol* (2004) 3:Article3. doi: 10.2202/1544-6115.1027.
12. Addgene. General Transfection.
13. Nanbakhsh A, Best B, Riese M, Rao S, Wang L, Medin J, et al. Dextran Enhances the Lentiviral Transduction Efficiency of Murine and Human Primary Nk Cells. *J Vis Exp* (2018) (131). Epub 2018/01/25. doi: 10.3791/55063.
14. Kerkar SP, Sanchez-Perez L, Yang S, Borman ZA, Muranski P, Ji Y, et al. Genetic Engineering of Murine Cd8+ and Cd4+ T Cells for Preclinical Adoptive Immunotherapy Studies. *J Immunother* (2011) 34(4):343-52. Epub 2011/04/19. doi: 10.1097/CJI.0b013e3182187600.
15. Delville M, Soheili T, Bellier F, Durand A, Denis A, Lagresle-Peyrou C, et al. A Nontoxic Transduction Enhancer Enables Highly Efficient Lentiviral Transduction of Primary Murine

T Cells and Hematopoietic Stem Cells. *Mol Ther Methods Clin Dev* (2018) 10:341-7. Epub 2018/09/08. doi: 10.1016/j.omtm.2018.08.002.

16. Rudalska R, Dauch D, Longerich T, McJunkin K, Wuestefeld T, Kang TW, et al. In Vivo Rnai Screening Identifies a Mechanism of Sorafenib Resistance in Liver Cancer. *Nature medicine* (2014) 20(10):1138-46. doi: 10.1038/nm.3679.
17. Wuestefeld T, Pesic M, Rudalska R, Dauch D, Longerich T, Kang TW, et al. A Direct in Vivo Rnai Screen Identifies Mkk4 as a Key Regulator of Liver Regeneration. *Cell* (2013) 153(2):389-401. Epub 2013/04/16. doi: 10.1016/j.cell.2013.03.026.
18. Papadopoulos D, Ade CP, Eilers M. Generation of a Pooled Shrna Library for Functional Genomics Screens. *STAR Protoc* (2022) 3(1):101183. Epub 2022/03/05. doi: 10.1016/j.xpro.2022.101183.
19. Robinson MD, McCarthy DJ, Smyth GK. Edger: A Bioconductor Package for Differential Expression Analysis of Digital Gene Expression Data. *Bioinformatics* (2010) 26(1):139-40. Epub 2009/11/17. doi: 10.1093/bioinformatics/btp616.
